# Madagascar ground gecko genome analysis characterizes asymmetric fates of duplicated genes

**DOI:** 10.1186/s12915-018-0509-4

**Published:** 2018-04-16

**Authors:** Yuichiro Hara, Miki Takeuchi, Yuka Kageyama, Kaori Tatsumi, Masahiko Hibi, Hiroshi Kiyonari, Shigehiro Kuraku

**Affiliations:** 1 Phyloinformatics Unit, RIKEN Center for Life Science Technologies, Kobe, Hyogo 650-0047 Japan; 2 Laboratory for Phyloinformatics, RIKEN Center for Biosystems Dynamics Research, Kobe, Hyogo 650-0047 Japan; 3 Laboratory of Organogenesis and Organ Function, Bioscience and Biotechnology Center, Nagoya University, Nagoya, Aichi 464-8601 Japan; 4 Graduate School of Science and Technology, Kwansei Gakuin University, Sanda, Hyogo 669-1337 Japan; 5 Division of Biological Science, Graduate School of Science, Nagoya University, Nagoya, Aichi 464-8602 Japan; 6 Animal Resource Development Unit, RIKEN Center for Life Science Technologies, Kobe, Hyogo 650-0047 Japan; 7 Genetic Engineering Team, RIKEN Center for Life Science Technologies, Kobe, Hyogo 650-0047 Japan; 8 Laboratory for Animal Resource Development, RIKEN Center for Biosystems Dynamics Research, Kobe, Hyogo 650-0047 Japan; 9 Laboratory for Genetic Engineering, RIKEN Center for Biosystems Dynamics Research, Kobe, Hyogo 650-0047 Japan

**Keywords:** gecko, phylome, gene repertoire evolution, gene loss, gene duplication, disparity of genomic fields

## Abstract

**Background:**

Conventionally, comparison among amniotes – birds, mammals, and reptiles – has often been approached through analyses of mammals and, for comparison, birds. However, birds are morphologically and physiologically derived and, moreover, some parts of their genomes are recognized as difficult to sequence and/or assemble and are thus missing in genome assemblies. Therefore, sequencing the genomes of reptiles would aid comparative studies on amniotes by providing more comprehensive coverage to help understand the molecular mechanisms underpinning evolutionary changes.

**Results:**

Herein, we present the whole genome sequences of the Madagascar ground gecko (*Paroedura picta*), a promising study system especially in developmental biology, and used it to identify changes in gene repertoire across amniotes. The genome-wide analysis of the Madagascar ground gecko allowed us to reconstruct a comprehensive set of gene phylogenies comprising 13,043 ortholog groups from diverse amniotes. Our study revealed 469 genes retained by some reptiles but absent from available genome-wide sequence data of both mammals and birds. Importantly, these genes, herein collectively designated as ‘elusive’ genes, exhibited high nucleotide substitution rates and uneven intra-genomic distribution. Furthermore, the genomic regions flanking these elusive genes exhibited distinct characteristics that tended to be associated with increased gene density, repeat element density, and GC content.

**Conclusion:**

This highly continuous and nearly complete genome assembly of the Madagascar ground gecko will facilitate the use of this species as an experimental animal in diverse fields of biology. Gene repertoire comparisons across amniotes further demonstrated that the fate of a duplicated gene can be affected by the intrinsic properties of its genomic location, which can persist for hundreds of millions of years.

**Electronic supplementary material:**

The online version of this article (10.1186/s12915-018-0509-4) contains supplementary material, which is available to authorized users.

## Background

**Fig. 1:**
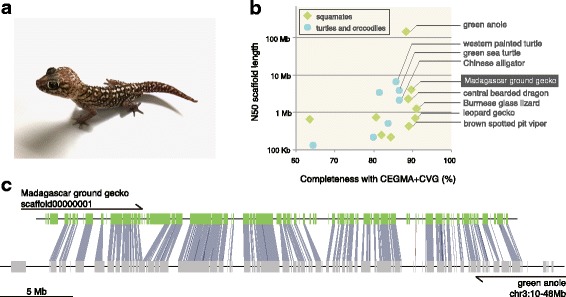
Quality assessment of the *P. picta* genome assembly. (**a**) *P. picta* adult individuals with different color phases. (**b**) A scatter plot of the assessments of assembly quality: CEGMA completeness scores referring to the CVG and N50 scaffold lengths for available reptile genome assemblies. Each reptile genome assembly is shown with a dot. Detailed statistics of assembly quality are included in Additional file 1: Table S2. These metrics were calculated with the webserver gVolante [90]. (**b**) Synteny conservation between the longest scaffold of the *P. picta* genome assembly (scaffold00000001; 33 Mb long) and a part of the green anole chromosome 3 (10–48 Mb in 204 Mb long). Green and gray boxes are protein-coding genes in the gecko and anole genome assembly, respectively, and blue bands indicate orthology between gecko and anole genes

Reptiles and birds, together composing the evolutionary clade Sauropsida, are the evolutionarily closest living companions to mammals [1]. In modern biology, chicken has been established as the primary study system to address the origin of characters shaping mammals [2]. However, birds, which are descendants of a reptilian lineage that originated from dinosaurs, have a variety of highly derived morphological characters, including feathers, wings, and toothless beaks. Furthermore, recent studies revealed that regional characteristics in avian genomes could hinder sequence assembly in those or some regions, resulting in the exclusion of hundreds of genes [3–5] that were previously considered to be evolutionarily lost [6]. Therefore, in order to conduct comparative analyses, the use of reptiles in place of birds as the closest relatives to mammals is a reasonable choice in phylogenetic contexts. Nevertheless, reptiles currently do not serve as practical study systems with regards to feasibility in operational experiments and accessibility to comprehensive molecular sequence information. The Madagascar ground gecko (*Paroedura picta*; Fig. 1a), belonging to the family Gekkonidae, is a reptile candidate that meets these requirements. In captivity, females of this species lay two eggs every 10 days all year round [7], which allows a stable supply of embryonic material. Additionally, the relatively thin eggshells enable *in ovo* manipulation [7, 8], and embryonic stages of this species have already been categorized [7]. With these developmental biology advantages, embryos of this gecko species are becoming frequently used for various studies in this field, revealing the evolutionary histories of molecular mechanisms of cortical neurogenesis and gastrulation in amniotes [9, 10], which has not yet been accomplished using only mammals and birds. Among reptiles, the green anole is relatively popular in biological research, especially at the molecular level, supported by its genomic sequence resources. However, its seasonal breeding and soft eggshell [11] have limited the conduct of continuous and operational experiments. Given these issues, genome sequences of the Madagascar ground gecko, with higher feasibility in experimentation, will facilitate molecular comparative studies involving this species in diverse research fields.

In addition to the comparison of morphologies, a reasonable taxon sampling in phylogenetic contexts can revise evolutionary scenarios of gene repertoire in amniotes previously proposed through the use of fewer species. For example, molecular phylogenetic trees including reptiles consolidated the orthology/paralogy inference of human *Nodal* [12] and *Oct4* [13] genes to the corresponding chicken genes. A comprehensive set of gene phylogenies is referred to as a phylome [14], and reconstructing a phylome with a broad taxon sampling is applicable to an intensive search for genes whose orthologs are absent from model mammalian and bird genomes but are present in some reptiles; indeed, these genes could be thought to have been lost in the common ancestor of amniotes if reptilian genomes are not employed in the search. In this strategy, genes missing in genomic sequences of multiple lineages can be systematically analyzed, providing an insight into the mechanisms of gene repertoire diversification. However, studies of the evolution of gene repertoire have thus far largely concentrated on gene duplication.

In this study, we conducted *de novo* whole genome sequencing of the Madagascar ground gecko. By including the inferred genes in its genome, we have reconstructed a phylome. Remarkably, our results uncovered hundreds of genes retained by reptiles but independently absent from both mammalian and avian genomes. Comparing these genes with their paralogs, we discussed causal factors of the asymmetric fates of these paralogs during vertebrate evolution.

## Results

### Genome sequencing

The *P. picta* genome was sequenced with 75× coverage as paired-end and mate-pair libraries on the Illumina platform (Additional file 1: Table S1), resulting in a genome assembly of 1.69 Gb (Table 1). This size was close to the measure of its haploid nuclear DNA content in somatic cells of *P. picta* (1.80 Gb; Additional file 1: Table S3). The genome assembly had an N50 scaffold length of 4.1 Mb and an overall GC content of 44.8% (Table 1). A total of 37.3% of the genome assembly consisted of repetitive elements (Additional file 1: Table S4, Additional file 2: Figure S1). Gene prediction was performed with the program Augustus, incorporating the optimized prediction parameters for the species and prediction hints based on transcript evidence and protein-level homology to other amniotes. The initial set of gene models was polished as performed previously [14] (see Methods), which resulted in the final set of 27,039 protein-coding genes (Table 1, Additional file 1: Table S5).

**Table 1:** Statistics of the *Paroedura picta* genome assembly

assembly/annotation feature	statistic
Number of scaffolds	110,906
Total size of scaffolds (bp)	1,694,174,484
Longest scaffold (bp)	33,658,631
Shortest scaffold (bp)	500
N50 scaffold length (bp)	4,106,116
Scaffold GC content (%)	44.76
Scaffold Gap content (%; percentage of Ns)	8.99
CEGMA+CVG, ‘Complete’ score (%)	89.70
CEGMA+CVG, ‘Partial’ score (%)	98.71
Number of coding genes	27,039
Median coding sequence length (bp)	912
Coding sequence GC content (%)	52.27
Average number of coding exons	7.12

We assessed the completeness of the genome assembly with CEGMA [15], using the 233 core vertebrate genes (CVG) as a reference [16], as well as BUSCO referring to its vertebrate ortholog set [17]. The assessments demonstrated that the *P. picta* genome assembly harbored comparably high completeness scores (nearly 90% or higher) to several other squamates (Fig. 1b, Additional file 1: Table S2). Additionally, the *P. picta* genome assembly had the third largest N50 scaffold length among reptiles, surpassed only by the assemblies of the green anole and western painted turtle, which were both produced employing Sanger sequencing technology (Additional file 1: Table S2). The continuity was further confirmed by a large-scale conserved synteny between the longest scaffold (33 Mb long) and a region on the anole chromosome 3 (Fig. 1c), as well as the unambiguous retrieval of all four of the entire Hox clusters (Additional file 1: Table S6). In summary, these assessments revealed both high completeness and high continuity of the *P. picta* genome assembly.

The Madagascar ground gecko belongs to Squamata, the same order as the green anole, whose genome harbors unusually low intragenomic GC heterogeneity [18]. The overall GC content of the *P. picta* genome was higher than that of the green anole genome and comparable to those of the turtle and crocodile genomes (Additional file 3: Figure S2a). However, the variance in GC content distribution in the *P. picta* genome was close to that of the green anole genome, especially when using narrow sliding windows (Additional file 3: Figure S2b). A principal component analysis of diverse amniote genomes on the frequencies of 3-mer nucleotides demonstrated that the first principal component is responsible for GC content and, interestingly, the third principal component clearly separated the squamate genomes from those of the other amniotes (Additional file 1: Supplementary Text, Additional file 3: Figure S2c). The results indicate that the genomes of the common squamate ancestors experienced a decrease in the local heterogeneity of GC content, which has further progressed in the extant anole genomes [18].

### Reconstruction of amniote phylome

**Fig. 2:**
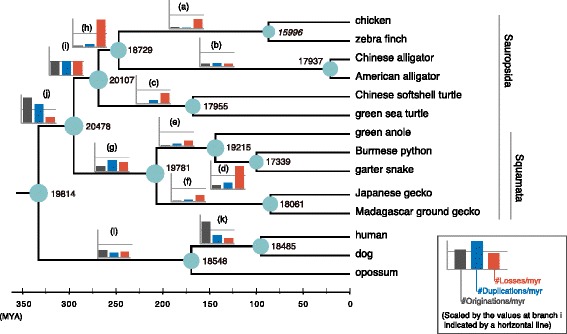
Quantification of gene origination, duplication, and loss in amniote evolution. Bars beside inner branches of the amniote species tree denote numbers of origination, duplication, and loss of genes per million years scaled by the numbers at the branch (i). Gene origination, duplication, and loss events in the terminal branches are not counted because they can be largely influenced by rampant misidentifications in gene prediction. Additionally, inferred ancestral gene repertoires are shown at internal nodes of the tree. Branch lengths are proportional to inferred divergence times shown in Additional file 5: Figure S3, and the details of these values are included in Table 2. The gene number at the ancestral nodes of birds shown in italics is likely underestimated due to underrepresentation of certain genomic regions in whole genome sequencing [3–5]

To clarify the tempo and mode of gene repertoire evolution in sauropsids, we produced a comprehensive set of gene trees, called a phylome, by grouping homologs of amniotes, reconstructing the molecular phylogenetic trees of the individual homolog groups, and dating gene duplication and loss events in the trees (see Methods). We reconstructed maximum-likelihood trees of the 13,043 ortholog groups, each of which contained at least one amniote, using Madagascar ground gecko, Japanese gecko, green anole, Burmese python, garter snake, two turtles, two alligators, two birds, and three mammals, as well as six non-amniote vertebrates (Additional file 1: Table S7, Additional file 4: Table S8). These gene trees were reconciled with the species tree by introducing rearrangements of topologies of the gene trees with low support values (e.g., bootstrap values) in order to resolve topological incongruence caused potentially by methodological variability or incomplete lineage sorting [19]. From the 13,043 ortholog groups, we selected the 1545 groups that consisted of one-to-one orthologs of all 14 tetrapods examined and exhibited tree topologies consistent with the species tree. Using multiple alignments of these one-to-one ortholog groups and a fixed species tree topology, the divergence times of the amniote lineages were inferred by employing an MCMC framework with fossil calibrations at several nodes of the species tree (Additional file 5: Figure S3). Finally, gene origination, duplication, and loss were dated within the reconciled gene trees [19], and the estimated numbers of these events were summed and scaled by time for every inner branch in the species tree (Fig. 2).

The phylome revealed that the numbers of gene origination and duplication events per million years in the early lineages of sauropsids and squamates (branches g, i, and j in Fig. 2, Table 2) were 1.2–35 and 2.3–16 times as high as those in the other more recent inner branches (branches a–f in Fig. 2, Table. 2), respectively. On the other hand, the gene loss rate exhibited a different trend from that of the origination and duplication rates. Using this information of gene origination, duplication, and loss, we reconstructed the gene repertoires of the ancestral lineages in amniotes, whose numbers are shown at the ancestral nodes in Fig. 2. The numbers of genes at the ancestral nodes indicate that the gecko ancestors harbored more genes than the respective common ancestors of the two selected species of snakes, turtles, alligators, and birds.

**Table 2:** Gene origination, duplication, and loss rates in the ancestral sauropsid lineages

Taxon	Branch^a^	Time (myr)	#gene originations	#gene duplications	#gene losses	#originations/myr	#duplications/myr	#losses/myr
Neognathae	(a)	160.3	54	222	2644	0.3369	1.6843	19.1703
Crocodylia	(b)	225.6	134	776	1272	0.5940	3.6392	7.6640
Testudines	(c)	97.74	13	352	1814	0.1330	3.8469	25.4348
Serpentes	(d)	44.52	37	291	1791	0.8311	7.1878	49.7305
Toxicofera	(e)	64.4	11	163	593	0.1708	3.4317	11.8323
Gekkonidae	(f)	122.2	32	270	1505	0.2619	2.4386	16.2684
Squamata	(g)	86.05	88	988	1426	1.0227	16.3626	24.3579
Archosauria	(h)	21.87	8	71	1062	0.3658	4.2524	64.5633
Archelosauria	(i)	26.47	70	373	664	2.6445	22.1760	35.3986
Sauropsida	(j)	37.4	175	707	422	4.6791	27.5401	16.3369
Boreoeutheria	(k)	74.27	303	613	705	4.0797	8.8461	11.7813
Mammalia	(l)	163.27	233	872	1783	1.4271	5.8370	14.7486

^a^Shown in Fig. 2

*myr* million years

### Evolutionary history of Gekkonidae uncovered by the phylome

According to the inference of the divergence times performed in the previous section, the ancestors of *Paroedura* and *Gekko* genera separated 87.2 million years ago (MYA; 95% CI 85.5–88.6 MYA; Additional file 5: Figure S3), which corresponds to the late Cretaceous. This divergence time was consistent with an estimate based on a molecular phylogenetic study employing a large-scale taxon sampling of Gekkonidae with several loci [20]. Additionally, it was revealed that the *Paroedura*-*Gekko* separation corresponds to the divergence between the African/Madagascar and Asian geckos, the oldest split within Gekkonidae [20, 21].

The one-to-one ortholog relationships were further utilized to examine the tempo of neutral nucleotide substitutions in the gecko lineage. We computed the number of synonymous substitutions per site (*K*_S_) between the Madagascar ground gecko and Japanese gecko, as well as between the Madagascar ground gecko and green anole, using 12,889 and 12,330 one-to-one ortholog pairs, respectively (Additional file 1: Table S9). The medians of the *K*_S_ were estimated at 0.230 and 0.823, respectively (Additional file 6: Figure S4a, b). These values were scaled by the inferred divergence times, namely 87.2 MYA for the two geckos’ split and 209.4 MYA for the anole-gecko split (Additional file 5: Figure S3), resulting in 0.00243 and 0.00393 (*K*_S_*/*million years (myr)), respectively. This result suggested a remarkable slowdown in molecular evolutionary rates in Gekkonidae, though squamates are considered to be one of the taxonomic groups harboring the fastest molecular evolutionary rates in amniotes [22]. The *K*_S_ distribution of the ortholog pairs of the two gecko species were also compared with those of other species pairs that split at comparable divergence times to the geckos, namely chicken and zebra finch for birds, and human and dog for mammals. Using the 6231 one-to-one ortholog groups retained by all six amniotes, the distribution of the geckos’ *K*_S_ scaled by time (median 0.00243 *K*_S_*/*myr) was significantly different from those between chicken and zebra finch (divergence time, 86.8 MYA; median 0.00487 *K*_S_/myr; Wilcoxon signed-rank test, *p* < 2.2 × 10^− 16^) and between human and dog (divergence time, 95.3 MYA; median 0.00361 *K*_S_/myr; Wilcoxon signed-rank test, *p* < 2.2 × 10^− 16^) (Additional file 6: Figure S4c). The results also suggest that the nucleotide substitution rates in the gecko lineage have been lower for at least 90 myr.

### Molecular dissection of gecko phenotypes

A comparison of gene repertoires between the Madagascar ground gecko and Japanese gecko allows the reconstruction of ancestral characters of the family Gekkonidae. One of the remarkable characters shared by most geckos is nocturnality [20], which may have accompanied the loss of a few visual opsin genes at least in the species belonging to the genus *Gekko* [23, 24]. However, the evolution of the opsin gene repertoire in the entire Gekkonidae family has not yet been uncovered. In our study, whole genome sequencing revealed that the Madagascar ground gecko possesses three visual opsins, rhodopsin 2 (*RH2*), short wavelength-sensitive opsin 1 (*SWS1*), and long wavelength-sensitive opsin (*LWS*), but has lost rhodopsin (*RH1*) and short wavelength-sensitive opsin 2 (*SWS2*), as have other *Gekko* species [23, 24] (Additional file 7: Figure S5). As in the previous observation for the Japanese gecko [24], the *RH1* locus in the Madagascar ground gecko, located on scaffold00000009, was shown to be pseudogenized with frame shifts. Furthermore, the *RH2* genes of the Madagascar ground gecko and Japanese gecko were revealed to share amino acid substitutions that are known to be associated with a shift to shorter wavelength in the absorption spectrum, specifically F89C and T97A [23]. These observations indicate that the common ancestor of Gekkonidae, which is considered to have been nocturnal [20], already possessed the shrunken set of visual opsins uniquely observed for modern geckos.

The toepad is another defining characteristic of geckos, and its shape and climbing ability have diversified depending on the geckos’ habitats [25]. Previously, 71 beta-keratin genes, major components of the setae of toepads, were detected in the genome of the Japanese gecko, which is capable of climbing a smooth surface [24]. On the other hand, our search detected 120 beta-keratin genes in the genome of the Madagascar ground gecko, which has a weaker climbing ability [25]. The phylogenetic tree of beta-keratin genes exhibited explosive gene duplications that had already initiated prior to the split of these two gecko species (Additional file 8: Figure S6). This finding suggests that the common ancestors of Gekkonidae had already acquired a diversified beta-keratin gene set related to the specialized toepads, potentially leading to remarkable variety in their morphologies in modern geckos.

### ‘Elusive’ genes missing in mammals and birds but present in some reptilian species

Our amniote phylome (Fig. 2) displays the fine-scale evolutionary histories of the gene repertoires. It potentially includes the genes whose orthologs were missing in mammals and birds but retained by some reptiles, which had not been uncovered by a comparative analysis conducted between only mammals and birds. As a first screening, we scanned the phylome for genes whose orthologs were identified in at least two reptilian species and absent from all the mammalian or avian genome assemblies within the individual gene phylogenies we reconstructed (see Methods). This search identified 469 genes, and hereafter, we designated them as ‘elusive’ genes (Additional file 9: Table S10). Of these genes, 263 possessed at least one paralog that was considered to have been duplicated in early vertebrates (i.e., in two-round whole genome duplications) and retained by wider taxonomic groups (Additional file 9: Table S10). In this study, these paralogs are designated ‘non-elusive’ genes, as opposed to ‘elusive’ genes. Within the *P. picta* genome, we identified the orthologs of 259 elusive genes out of 469. Additionally, these elusive genes included 156 orthologs that retained their non-elusive paralogs in the *P. picta* genome. We analyzed these 156 gene pairs of *P. picta* in the comparison between elusive and non-elusive genes in the following analyses.

**Fig. 3:**
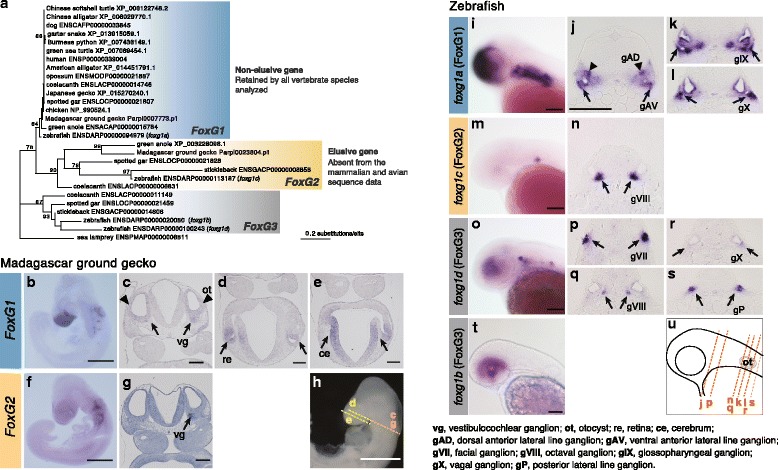
Asymmetric evolution of FoxG genes. (**a**) The maximum likelihood tree of the FoxG genes using 243 aligned sites of amino acid sequences with the JTT + I + G4 amino acid substitution model. The ultrafast bootstrap approximation values of 75 or more are indicated at the nodes. *FoxG2* and *FoxG1* are categorized in the elusive and non-elusive genes defined in this study, respectively. The absence of *FoxG2* orthologs in mammals and birds may be attributable to information loss or secondary gene loss (see Discussion). Whole-mount *in situ* hybridization for *FoxG1* (**b**) and *FoxG2* (**f**) genes using 3 and 4 dpo embryos of the Madagascar ground gecko, respectively. Expression signals of *FoxG1* (**c**–**e**) and *FoxG2* (**g**) by sections of *in situ* hybridization using 2 dpo embryos. *FoxG1* is expressed in the otocyst, vestibulocochlear ganglion (shown by arrowheads in **c**), retina (shown by arrowheads in **d**), and cerebrum (shown by arrowheads in **e**), whereas *FoxG2* is specifically expressed in the vestibulocochlear ganglion (shown by an arrow in **g**). (**h**) The levels of the sections for the gecko embryo are indicated by dashed lines. Whole-mount *in situ* hybridization for *foxg1a* (**i**, the FoxG1 ortholog), *foxg1c* (**m**, the FoxG2 ortholog), and *foxg1d* (**o**) and *foxg1b* (**t**) (the FoxG3 orthologs) using 2 dpf zebrafish embryos. Expression signals of *foxg1a* (**j**–**l**), *foxg1c* (**n**), and *foxg1d* (**p**–**s**) by sections of *in situ* hybridization using the 2 dpf embryos. Any expression signals of *foxg1b* were not observed in the ganglion. (**u**) The levels of the sections for the zebrafish embryos are indicated by dashed lines. Scale bars in **b**–**f**, 1 mm, and those in **i**, **j**, **m**, **o**, and **t**, 100 μm. **k**, **l**, **n**, and **p**–**s** have the same scale as **j**

The identified elusive genes included a member of the FoxG group, the forkhead box-containing transcription factor (Fox) gene family. The *FoxG1* gene, exhibiting features of a non-elusive gene, has been retained by all vertebrate species analyzed so far and plays a pivotal role in the differentiation of telencephalon and neocortex [26, 27]. In contrast, one of its paralogs, *FoxG2*, is possessed by only bony fishes and reptiles, including *P. picta*, and meets the criterion to be recognized as an elusive gene (Fig. 3a). The molecular phylogeny of FoxG genes revealed that *FoxG2* has higher amino acid substitution rates than *FoxG1* (Fig. 3a).

**Fig. 4:**
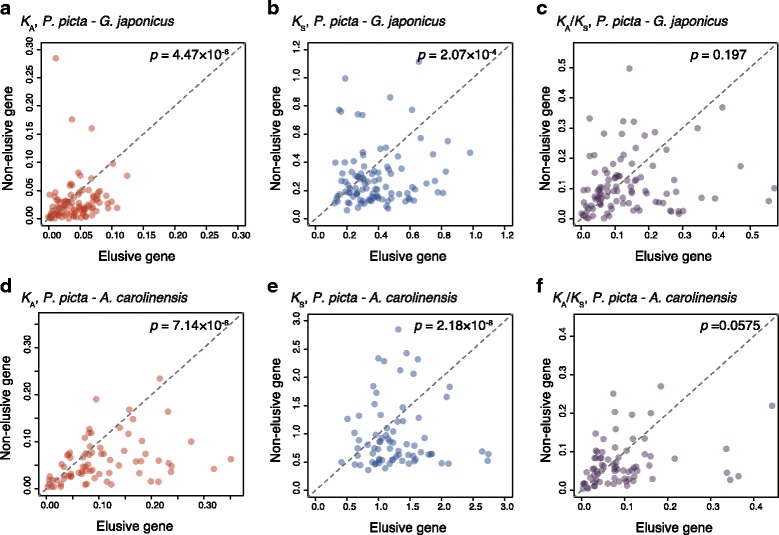
Evolutionary rates of elusive genes and non-elusive genes. Scatter plots of the (**a**) *K*_A_, (**b**) *K*_S_, and (**c**) *K*_A_/*K*_S_ values of the orthologs of the elusive genes and their non-elusive paralogs between Madagascar ground gecko and Japanese gecko, and those of the (**d**) *K*_A_, (**e**) *K*_S_, and (**f**) *K*_A_/*K*_S_ values of the orthologs between Madagascar ground gecko and green anole. Each dot denotes an ortholog pair of elusive genes and their non-elusive paralogs. All of the statistical tests were conducted with the Wilcoxon signed-rank test

To examine if the gene elusiveness is associated with such asymmetric evolutionary rates between paralogs as demonstrated in the FoxG gene tree, we calculated the number of non-synonymous substitutions per site (*K*_A_) for the 156 pairs of elusive and non-elusive genes in *P. picta*. The result showed that *K*_A_ values for the elusive genes were significantly larger than those for their non-elusive paralogs (*p* = 4.47 × 10^− 6^; Fig. 4a). Interestingly, these elusive genes also exhibited significantly larger numbers of synonymous substitutions per site (*K*_S_) than did their non-elusive paralogs (*p* = 2.07 × 10^− 4^; Fig. 4b), although the *K*_A_/*K*_S_ values were comparable (*p* = 0.197; Fig. 4c). Furthermore, the elusive genes identified in the anole genome exhibited larger *K*_A_ and *K*_S_ values than their non-elusive paralogs (Fig. 4d–f). These observations indicate that a significantly high mutation rate accounts for the rapid rate of amino acid substitution in elusive genes.

**Fig. 5:**
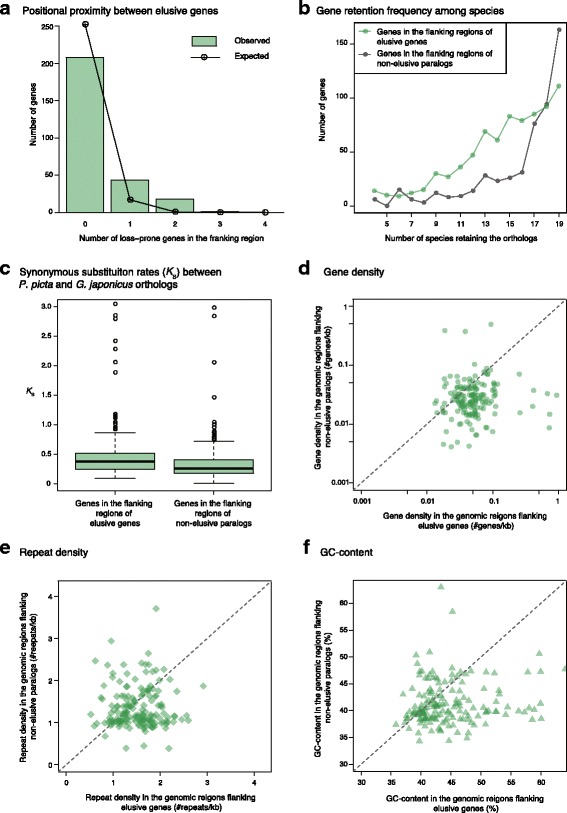
Genomic characteristics of elusive genes and their non-elusive paralogs in the *P. picta* genome. (**a**) Fractions of the 259 elusive genes flanked by other elusive genes (100 kb on both ends). The expected values, which assumed a random distribution of elusive genes, are shown in a dotted line. The observed and expected values are significantly different (*p* = 8.27 × 10^− 27^, the exact test of goodness-of-fit). (**b**) Frequency distributions of the genes flanking the elusive and non-elusive genes related to the numbers of species retaining the orthologs. The genes that harbored one or more actinopterygians or coelacanth orthologs were used. The two distributions are significantly different from each other (*p* = 4.18 × 10^− 19^, Mann–Whitney *U* test). (**c**) Comparison of synonymous substitution rates (*K*_S_) between the genes flanking the elusive and the non-elusive genes. The distributions are significantly different between the two groups (*p* = 1.23 × 10^− 17^, Mann–Whitney *U* test). The *K*_S_ values were computed for the individual ortholog pairs of the Madagascar ground gecko and Japanese gecko. (**d**) Gene densities of the flanking regions of the elusive genes and their non-elusive paralogs, which are significantly different from each other (*p* = 4.44 × 10^− 16^, Wilcoxon signed-rank test). Each dot denotes the gene densities of the flanking regions of an elusive gene and its non-elusive paralog. (**e**) Repeat densities of the genomic regions consisting of the elusive genes or non-elusive paralogs with their flanking regions, which are significantly different between the two groups (*p* = 1.58 × 10^− 4^, Wilcoxon signed-rank test). The repeats annotated with RepeatMasker excluding simple repeats and low-complexity regions were used. (**f**) GC content of the genomic regions consisting of the elusive genes or non-elusive paralogs with their flanking regions, which exhibit a significant difference between the two groups (*p* = 1.11 × 10^− 10^, Wilcoxon signed-rank test)

For more detailed characterization, we scanned the genomic locations of the 259 elusive genes identified in the *P. picta* genome. The result revealed that elusive genes were more frequently flanked by other elusive genes than expected, indicating their significantly uneven distribution (*p* = 8.27 × 10^− 27^; Fig. 5a). We further compared the genomic regions harboring the 156 elusive genes with those harboring their non-elusive paralogs in the *P. picta* genome. The result showed that the orthologs of genes flanking elusive genes tended to be retained by fewer species than those flanking their non-elusive paralogs (*p* = 4.18 × 10^− 19^; Fig. 5b). Additionally, the genes flanking elusive genes had larger *K*_S_ values than did those flanking non-elusive genes (*p* = 1.23 × 10^− 17^; Fig. 5c). Our observations suggest that the gene elusiveness and fast-evolving nature are largely influenced by local characteristics of the genomic regions harboring them. These genomic regions were characterized by high gene densities (*p* = 4.44 × 10^− 16^; Fig. 5d), high repetitive element densities (*p* = 1.58 × 10^− 4^; Fig. 5e), and high GC content (*p* = 1.11 × 10^− 10^; Fig. 5f) in comparison to the genomic regions containing their non-elusive paralogs. The contrast of local genomic characteristics was also observed in the green anole genome (Additional file 10: Figure S7), though GC content was not significantly different possibly due to the strong homogenization of GC content along this particular species [18] (Additional file 3: Figure S2). Our analysis further showed that not only squamates but also turtles and crocodiles harbored these genomic characteristics (Additional file 11: Figure S8, Additional file 12: Figure S9, Additional file 13: Figure S10), indicating that the genomic characteristics had emerged before the split between Lepidosauria and Archelosauria at the latest.

### Taxon-wide intensive search for elusive genes

The initial search for the elusive genes mentioned above employed the phylome of selected species, including three mammalian and two avian species. On the other hand, recent studies succeeded in identifying more than 2000 genes that were thought to be missing in the chicken genome through intensive searches employing abundant sequence information of diverse bird species [3, 4] or the genome assembly with a new single-molecule long read sequencing platform [3–5]. These findings prompted us to examine whether the 469 ‘elusive’ genes missing in the representative genomes (Additional file 9: Table S10) are present in other avian and mammalian species. For this purpose, we performed an in-depth search to identify as many orthologs of the elusive genes as possible. This search employed the genome-wide gene sets for 112 mammalian and 68 avian species and *de novo* transcriptome assemblies and expressed sequence tags for 66 mammalian and 55 avian species (Additional file 1: Supplementary Methods).

The result of this search demonstrated that, out of the 469 genes identified as ‘elusive,’ 157 retained at least one putative mammalian or avian ortholog. They included 48 elusive genes whose orthologs were retained by non-eutherian mammals or paleognaths but not eutherians and neognaths (Additional file 14: Figure S11), demonstrating the biased presence of the orthologs toward the taxonomic groups that were diverged from the other groups in the early mammals or birds. For the remaining 109 genes with the retained orthologs, putative orthologs were identified in highly variable numbers of species (from 1 to 101), while 57 of these genes were harbored by only 10 or fewer mammalian and avian species. The sequence data used for our ortholog detection included genome-wide gene sets of 14 mammals and two birds, as well as the transcriptome catalogs of four avian species, that were produced with the single molecule, real-time (SMRT) sequencing platform. The SMRT platform is free from PCR amplification and capable of unbiased sequencing in terms of GC content [28]. Thus, sequence data produced by this platform are expected to recover more genes in genomic regions with a high GC content that could be underrepresented in the Illumina sequencing platform. However, the SMRT platform-based sequence sets employed in our search recovered orthologs of 21 elusive genes at most (Additional file 1: Table S11). This low proportion of the orthologs recovered by the SMRT platform-based genome-wide sequences indicates that the absence of the orthologs in our results is likely to be better explained by reasons other than data absence caused by technical limitations.

Furthermore, we examined whether the genomic characteristics identified in the previous sections, including rapid nucleotide substitution rate, unequal distribution across the genome, and high GC content (Figs. 4 and 5, Additional file 10: Figure S7), were also exhibited by the elusive genes that retained the mammalian or avian orthologs (elusive gene subset 1), as well as the elusive genes whose mammalian or avian orthologs were not identified even in this in-depth search (elusive gene subset 2). The results showed that, in both elusive gene subsets, the null hypotheses that were rejected with the initial elusive gene set (Figs. 4 and 5, Additional file 10: Figure S7) were all rejected and that, in many of the experiments, the effect sizes of the statistical tests were comparable between the different sets of the elusive genes (Additional file 1: Table S12). Our findings indicate that the elusive genes retaining mammalian or avian orthologs harbored the common molecular evolutionary and genomic characteristics observed in those without mammalian and avian orthologs.

### Differential expression between elusive genes and their non-elusive paralogs

As the analysis in the previous section showed that the absence of the orthologs is not a prerequisite of the peculiar characteristic of the elusive genes, the following analyses focused on the 469 elusive genes initially identified. To elucidate the possible relevance of the nature of the elusive genes to their expression profiles, we compared expression levels in various tissues between elusive genes and their non-elusive paralogs. For this purpose, we retrieved RNA-seq reads of three embryonic stages of *P. picta* and 11 tissues of green anole released previously [16, 29]. Our gene expression quantification showed that 83% and 52% of elusive genes in the *P. picta* and green anole genomes, respectively, possessed expression levels lower than or comparable to those of their non-elusive paralogs within any of the analyzed tissues (fold-change ≤ 2 or fragments per kilobase of transcript per million mapped fragments (FPKM) < 1 for both of the genes; Additional file 15: Figure S12). This suggests that the expression levels of elusive genes tend to be lower than those of their non-elusive paralogs. Furthermore, only 16 and 22 elusive genes out of the 156 and 180 in the Madagascar ground gecko and green anole genomes, respectively, exhibited distinct expression levels from their non-elusive paralogs (fold-change > 10). On the other hand, 60 and 88 non-elusive genes of the Madagascar ground gecko and green anole, respectively, harbored the same trend as the elusive paralogs (fold-change < 0.1). These genes included a duplicate of the green anole *fox-1* gene encoding an RNA binding (Rbfox) protein, whose ortholog is missing in the mammalian and avian genomes and that retains a larger *K*_S_ value than that of its non-elusive gene (Additional file 1: Table S13). The anole transcriptome data suggested that, among the tissues examined, this elusive *Rbfox* gene was specifically expressed in adult muscle and heart (Additional file 1: Table S13). In contrast, *Rbfox3* (or *NeuN*), a non-elusive paralog of the elusive *Rbfox* gene, is a well-known neural biomarker [30], and its expression is absent from the muscle and heart. The zebrafish ortholog of this elusive gene, *rbfox1l*, was also specifically expressed in muscle and heart in embryos [31]. The expression profiles of the elusive gene of the Rbfox gene family could have been altered by the possible acquisition of unique expression profiles.

Furthermore, as a proxy for fine-scale comparison of post-duplication differentiation of expression domains, we investigated the embryonic expression patterns of *P. picta FoxG2* and *FoxG1*, the aforementioned pair of elusive and non-elusive genes. The transcriptome data revealed that *FoxG2* was expressed at lower levels than was *FoxG1* in all three embryonic stages examined (Additional file 16: Figure S13). The result of *in situ* hybridization using early embryos (2–4 dpo; days post oviposition) revealed the restricted expression domain of *FoxG2*, which was nested within those of *FoxG1* (Fig. 3b–h). *FoxG1* was expressed in the cerebrum, retina, otocyst, and vestibulocochlear ganglion, while the expression domain of *FoxG2* was restricted to the vestibulocochlear ganglion. *In situ* hybridization in zebrafish embryos also revealed a restricted expression of *foxg1c*, the zebrafish FoxG2 ortholog, compared to that of *foxg1a*, the zebrafish FoxG1 ortholog, although the expression domain of *foxg1c* was not nested in that of *foxg1a* (Fig. 3i–u, Additional file 16: Figure S13), rather *foxg1c* was expressed in the octaval ganglion, while *foxg1a* was expressed in the cerebrum, dorsal anterior lateral line, ventral anterior lateral line, glossopharyngeal ganglion, and vagal ganglion. Importantly, the vestibulocochlear ganglion in the gecko is considered homologous to the octaval ganglion in zebrafish [32], revealing that FoxG2 orthologs share their expression domain. Furthermore, in contrast to the *P. picta FoxG1*-*FoxG2* relationship, the zebrafish *foxg1a* is not expressed in the octaval ganglion, although *foxg1c* shares the expression domain with *foxg1d*, a zebrafish paralog of *FoxG3* missing in tetrapod genomes*.* The fine-scale expression analysis of the FoxG genes implies that an elusive gene may acquire its unique expression even in a small domain within a tissue that cannot be detected by tissue-level transcriptome analyses.

## Discussion

In this study, we produced the Madagascar ground gecko genome assembly, whose N50 scaffold length amounts to 4.1 Mb, employing the short-read sequencing platform. The high continuity of the assembly was achieved through a variety of targeted mate distances for mate-pair sequencing libraries (see Methods) [33], as well as by the *de novo* assembly program adaptable to highly heterozygous diploids [34]. A heterozygosity distribution across the *P. picta* genome indicated that the genome included regions with relatively high heterozygosity (~0.8% on average) (Additional file 17: Figure S14), which potentially hinders reconstruction of consensus haploid sequences [35]. However, the assembly size (1.69 Gb) was only slightly smaller than the genome size estimated by measuring the haploid DNA content (1.80 Gb), demonstrating that the genome assembly contained few artificial paralogs derived from heterozygous alleles. This speculation was further supported by the completeness assessments, which indicated that almost all of the reconstructed genes retained one-to-one orthology with the reference gene sets (95.2% for the CVG and 99.2% for the vertebrate BUSCO) (Additional file 1: Table S2). The use of the *P. picta* genome assembly, exhibiting high continuity and almost complete gene coverage (Fig. 1b), is expected to facilitate molecular studies on developmental biology as well as diverse comparative fields in biology.

The phylome was constructed through reconciliation of gene trees with the species tree, which revealed a new picture of gene repertoire evolution in amniotes (Fig. 2). In animal evolution, explosive gene origination and duplication seem to have predated the radiation of taxonomic groups, as observed in the origin of metazoans [36, 37] and radiation of cichlids [38]. Our results demonstrated the similar temporal relationship of frequent gene originations and duplications in the ancestors of sauropsids and squamates with the taxonomic radiations in these lineages (Fig. 2). The figure also indicated frequent gene originations and duplications in the lineage predating the eutherian radiation, which was supported by the previous gene gain-and-loss analysis using more mammalian species [39]. The phylome displayed the high conservation of gene repertoire in the ancestor of geckos (Fig. 2), suggesting that massive gene loss accompanying a large-scale exclusion of certain genomic regions is unlikely to be observed in the genome assemblies of geckos. In contrast, the phylome exhibited a large reduction in gene repertoire in the common ancestor of birds, which is becoming recognized as an underestimate due to a failure of gene prediction within the genomic regions missing in the whole genome assemblies [3, 4]. Notably, a comparison of conserved non-coding element repertoires among sauropsids indicated no large-scale compactions in the early avian lineage (Additional file 1: Supplementary Text, Additional file 18: Figure S15).

The amniote phylome also illuminated hundreds of ortholog groups that were missing in the mammalian and avian genomes but retained in some of the reptilian genomes (Additional file 9: Table S10). Absence of orthologs from multiple vertebrate lineages and plentiful accumulation of amino acid substitutions, which are similar to the characteristics of the ‘elusive’ genes, have been observed for several genes, including *Pax10* [40], *Dact4* [41], and *Bmp16* [42]. These genes have duplicates with trends similar to those of the non-elusive genes that arose in early vertebrates and have been rigidly retained by diverse vertebrate species. In a well-accepted view of the fates of gene duplicates, the heterogeneity of amino acid substitution rates among paralogs is assumed to be largely influenced by the difference in the degrees of functional constraints among them [43]. Accordingly, a variety of theoretical models have been introduced to explain the correlations between functional and genetic divergences of duplicated genes [44–49]. On the other hand, our study uncovered the characteristics of elusive genes and their flanking regions, elevated nucleotide substitution rates (Fig. 4), and increased gene density, repeat density, and GC content (Fig. 5, Additional file 10: Figure S7, Additional file 11: Figure S8, Additional file 12: Figure S9, Additional file 13: Figure S10). Importantly, these characteristics are unlikely to be directly associated with gene functions and, thus, asymmetry in them cannot be explained by the existing theoretical models based on gene functions.

In our study, we provisionally designated genes absent from the available mammalian and avian sequence sets as elusive genes. Due to the limitation of sequencing and assembly technologies, full reconstruction of genome assembly is not feasible, which hinders judgement of whether gene absence is due to evolutionary loss or information loss. Accumulating sequence data of various species based on more sequencing platforms is expected to facilitate identification of more elusive genes that were regarded as lost during evolution [3–5]. One of such recent studies employing an intensive search achieved the recovery of 86.9% of the avian orthologs that were retained by human and turtle but absent from the chicken genome assembly [4]. Additionally, three-fourths of the identified genes were retained by ten or more avian species [4]. This study provides proof of a method for retrieving genes that tend to be elusive.

As performed in the aforementioned study aimed for avian ortholog detection, but with more extensive sequence data, we further searched for the mammalian and avian orthologs. However, our result revealed lower recovery rates and species inclusion of the orthologs than did the previous study. Only 33.5% of mammalian or avian orthologs of the elusive genes (157 out of 469) were identified, and 59.6% of the identified orthologs of the elusive genes were retained by fewer than 10 mammalian and avian species (Additional file 14: Figure S11). The identified orthologs were not abundant within the sequence sets produced with the SMRT platform (Additional file 1: Table S11), which performs sequencing unaffected by GC content. Therefore, our identification of the limited number of orthologs of the elusive genes, through the intensive search, is likely to be better explained by other reasons, including genuine evolutionary gene loss, rather than by data absence due to technical limitations. This speculation would be confirmed with nearly complete genome assemblies of mammalian and avian species, which is expected to be achieved by the reference Vertebrate Genomes Project led by the G10 K consortium [50].

**Fig. 6:**
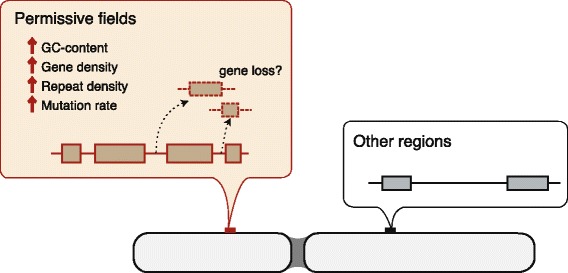
Disparity of genomic fields. A permissive field (a brown bar) that includes elusive genes displays elevated GC content, high gene density, high repeat density, and high mutation rate in comparison with other regions (a black bar)

More importantly, the elusive genes, whose definition in this study is not implicated in any evolutionary process in principle, exhibited particular genomic characteristics including rapid nucleotide substitutions, uneven distribution in the genomes, and high GC content (Additional file 1: Table S12). These characteristics were overrepresented in the elusive genes whether or not they retained the mammalian and avian orthologs. Altogether, it is suggested that the mechanism permitting the gene elusiveness and fast-evolving property operates extensively in the genomic regions harboring these characteristics. Our findings led to the theory that a genome is a composite of such ‘permissive’ regions and more conservative regions (Fig. 6). The disparity of local characteristics between different genomic regions would result in asymmetry of fates between gene duplicates.

The genomic characteristics observed in the ‘permissive’ fields that have a high gene density, high repeat density, and high GC content can be associated with genomic instability. Among the annotated known repeat elements in all of the reptile genomes studied, only SINE was overrepresented in the genomic regions including the elusive genes (Additional file 1: Table S14). Elevated GC content and repeat element density (e.g., SINE and (A)_n_ densities) are known to be associated with high recombination rates [51], which could increase mutation rates [51, 52]. Additionally, previous studies on mammalian genomes revealed that non-allelic homologous recombination, one of the mechanisms delivering CNVs including deletions, frequently occur in the genomic regions harboring high gene density, SINE density, and GC content [53–57]. Interestingly, microdeletions accompanying extrachromosomal circular DNAs are frequently observed in the genomic regions with high gene density and GC content in normal cells, including sperms, of adult mouse tissues [58]. Accordingly, the ‘permissive’ fields could allow deletions in the genomes through gametogenesis.

From the viewpoint of chromosome evolution, the vertebrate genome studies indicated that recombination rates are negatively correlated with chromosome sizes in at least some of the vertebrate genomes analyzed thus far [59, 60], suggesting that frequent recombinations facilitated the reduction of chromosome sizes. Remarkably, microchromosomes, extremely small chromosomes of some non-mammalian vertebrates, generally exhibit high recombination rates and high gene density, as well as high GC content and rapid nucleotide substitution rates [2, 61]. In our analysis, we identified 396 and 550 chicken orthologs of the *P. picta* genes flanking the elusive genes and their non-elusive paralogs in its genome, respectively. Interestingly, 71.2% of the genes in the former group were located in the chicken microchromosomes (chromosomes 9 to 38), while 51.8% of those in the former group were localized within the microchromosomes. The finding indicated a significantly different trend of the localization of the genes within the microchromosomes (Fisher’s exact test, *p* = 1.89 × 10^− 9^; odds ratio, 2.30). This finding implies that a large portion of the permissive fields might have belonged to ancient microchromosomes, many of which were thought to have originated prior to the last common ancestor of bony vertebrates [62]. The characteristics of the genomic regions derived from the microchromosomes may still have persisted even after their fusion into macrochromosomes in ancestral squamates at the latest, which allowed us to identify the permissive fields in the *P. picta* genome.

The phylome was reconstructed employing vertebrate-wide taxon sampling, which revealed that fewer orthologs of the elusive genes were retained in the amphibian and bony fish genomes than for those of the non-elusive genes (Additional file 1: Table S15). Our results also indicated that the degree of this trend was variable among the taxonomic groups. The bony fish genomes tended to retain more orthologs of the elusive genes than did the tetrapod genomes (Additional file 1: Table S15). This diminished the gene elusiveness in bony fishes seems to be consistent with their relatively moderate characteristics of the permissive fields; indeed, the elusive genes and their flanking regions in fish and amphibian genomes possess high gene density but do not exhibit a high GC content and repeat density (Additional file 11: Figure S8, Additional file 12: Figure S9, Additional file 13: Figure S10). These findings suggest that, during the evolution of amniotes, GC content and repeat density within the permissive fields were elevated, resulting in the consolidation of the disparity of local characteristics between different genomic regions. This consolidation of disparity within the amniote genomes also explains the finding that the identified elusive genes are not always possessed by all reptiles (Additional file 1: Table S15). Supporting this assumption, the green anole genome, which exhibited secondarily diminished heterogeneity of GC content in the elusive genes and flanking regions, possesses the largest number of orthologs of the elusive genes among the reptiles analyzed.

The fast evolution of both amino acid and nucleotide sequences of the elusive genes (Fig. 4) indicates that their functions have been largely differentiated from the ancestral ones. Furthermore, the scrutiny of the *P. picta* and green anole RNA-seq data revealed that the elusive genes tend to have lower expression levels than or comparable to their non-elusive paralogs (Additional file 15: Figure S12). These results lead to an idea of the fate of genes in the permissive fields, namely that acceptance of mutations in coding and regulatory regions influenced by the rapid-evolving nature brings about reduction of functional constraints by losing the original protein functions and expression domains, potentially resulting in permission of gene loss.

At the same time, the results indicate that a small fraction of the elusive genes may have acquired novel expression domains or novel protein function. We detected 16 and 22 elusive genes of the Madagascar ground gecko and green anole, respectively, with higher expression levels than their non-elusive paralogs (fold-change > 10) in certain tissues or embryonic stages based on the RNA-seq data. Our detailed analysis of embryonic expressions of FoxG genes using the gecko and zebrafish illustrated restricted expression domains of the elusive genes, which were sometimes distinct from those of the non-elusive paralog (Fig. 3, Additional file 16: Figure S13). Furthermore, the comparison of *K*_A_ values between the elusive and non-elusive paralogs illustrated a limited number of such elusive genes that exhibited *K*_A_ values smaller than or comparable to their non-elusive paralogs (Fig. 4a, d). The deviation from the fast-evolving trend for these elusive genes implies an increase in functional constraints in a specific lineage. These elusive genes included a few genes belonging to the taste receptor type 1 gene family and the opsin gene family (Additional file 1: Supplementary Text, Additional file 7: Figure S5, Additional file 19: Figure S16), indicating that these sensory receptors have acquired unique function as a result of their adaptation to new environments. Taken together, it is speculated that the permissive fields may have conferred new functions to genes that once had possessed the characteristics related to elusive genes, though this type of genes could be a minority.

## Conclusions

Our study primarily aimed to provide whole genome sequence information of the Madagascar ground gecko, which is becoming more frequently utilized as an experimental animal, especially in developmental biology. Whole genome sequencing and subsequent analyses resulted in genome assembly with high continuity as well as almost complete reconstruction of the set of gene models. Taking advantage of accumulating large-scale sequence information of reptiles including the gecko, our study shed new light on the asymmetric evolution of paralogs, potentially featuring gene loss. Comprehensive screening of vertebrate phylogenetic trees, as well as massive gene and transcript data of mammals and birds, identified ‘elusive’ genes that were missing in the mammalian and avian lineages. Our comparison of the elusive genes with their non-elusive paralogs revealed the rapidly evolving nature of the elusive genes as well as the distinct characteristics of the genomic regions flanking them. This finding manifested the fate of duplicated genes affected by the intrinsic properties of genomic regions harboring them, in addition to functional constraints on their roles.

## Methods

### Sample source

Embryos of *P. picta*, whose sexes were unknown, were provided by the Animal Resource Development Unit, RIKEN CLST. Animal breeding and experiments were conducted in accordance with the guidelines approved by the Institutional Animal Care and Use Committee (IACUC), RIKEN Kobe Branch. Embryos and larvae of zebrafish (*Danio rerio*) with the Oregon AB genetic background were experimented on in accordance with the regulations on animal experiments approved by the Nagoya University animal experiment committee and the Guidelines for the Proper Conduct of Animal Experiments (Science Council of Japan). The zebrafish individuals were maintained at the Bioscience and Biotechnology Center, Nagoya University.

### Sequencing

Genomic DNA was extracted from an embryo using phenol-chloroform followed by precipitation with ethanol. For paired-end library preparation, the extracted genomic DNAs were sheared with Focused-ultrasonicator S220 (Covaris) to retrieve DNA fragments of variable length distributions (see Additional file 1: Table S16 for detailed information about amounts of starting DNA and conditions for shearing). Library preparation was performed employing a KAPA LTP Library Preparation Kit (KAPA Biosystems). The paired-end libraries were prepared without PCR amplification, and the mate-pair libraries were prepared using optimal numbers of PCR cycles for individual libraries, which were determined with the KAPA Real-Time Library Amplification Kit (KAPA Biosystems) by preliminary qPCR-based quantification using an aliquot of adaptor-ligated DNAs. Small molecules in the prepared libraries were removed by size selection using Agencourt AMPure XP (Beckman Coulter Inc.). Numbers of PCR cycles and conditions of size selection for individual libraries are included in Additional file 1: Table S16. Mate-pair libraries were prepared using Nextera Mate Pair Sample Prep Kit (Illumina), employing our customized iMate protocol [33]. The details of mate-pair library preparation are included in Additional file 1: Table S16.

After size selection, the prepared libraries were quantified using KAPA Library Quantification Kits (KAPA Biosystems). These libraries were sequenced on a HiSeq 1500 (Illumina), operated by HiSeq Control Software v2.0.12.0 using HiSeq SR Rapid Cluster Kit v2 and HiSeq Rapid SBS Kit v2, and MiSeq (Illumina), operated by MiSeq Control Software v2.3.0.3 using MiSeq Reagent Kit v3 (Illumina). Read lengths were 151 or 171 nt on HiSeq and 301 nt on MiSeq. Base calling for the HiSeq output was performed with Real-Time Analysis v1.17.21.3, and the fastq files were generated with bcl2fastq v1.8.4 (Illumina). In the case of MiSeq, Real-Time Analysis v1.18.42 and MiSeq Reporter v2.3.32 were used. The resultant fastq files were subject to quality control with FastQC v0.10.1 [63]. Removal of low-quality bases and the Illumina adapter sequences from paired-end reads was processed by Trim Galore v0.3.3 [64], in which cutadapt v1.1 [65] was run with options ‘--stringency 2 --quality 20.’ Processing of mate-pair reads was performed using NextClip v1.1 [66] with its default parameters, and all processed reads within Category A-D by NextClip were used in genome scaffolding. Total numbers of reads and their nucleotide lengths for individual libraries, before and after read trimming, are listed in Additional file 1: Table S1.

### Genome assembly

*De novo* genome assembly and scaffolding employing the processed short reads were carried out using the program Platanus v1.2.1 [34] with its default parameters. The assembly step employed paired-end reads as well as singletons whose pairs were filtered, and the scaffolding step employed paired-end and mate-pair reads. The gap closure step employed all of the single, paired-end, and mate-pair reads. Scaffolds shorter than 500 bp were discarded from the genome assembly. Resultant scaffold sequences were further screened for contaminated organismal and artificial sequences employing DeconSeq v0.4.3 [67] (Additional file 1: Supplementary Methods; Additional file 20: Figure S17).

### Assembly quality assessment

The N50 scaffold lengths of the genome assemblies of *P. picta* and other reptiles were computed using the Perl script assemblathon_stats.pl [68] developed by the Assemblathon project [69]. CEGMA v2.5 [15] referring to the CVG [16] was utilized in order to assess the completeness of the assemblies. For the CEGMA runs, GeneWise v2.2.3-rc7 was employed [70]. BUSCO v2.0.1 referring to its vertebrate gene set was further used for this purpose [17].

### Repeat masking

Conventional and *de novo* repeat motifs in the *P. picta* genome assembly were modeled with RepeatModeler v1.0.8 [71] with its default parameters. Using this model, repeats were predicted with RepeatMasker v4.0.5 [72] with the ‘-xsmall’ option. Additionally, RepeatMasker with the ‘-xsmall -nolow’ option was executed to produce the masked assembly utilized for gene prediction. The repeat annotations of the green anole, Mississippi alligator, western clawed frog, coelacanth, spotted gar, stickleback, and zebrafish, all of which were predicted based on the RepeatMasker Library db20140131, were obtained from the RepeatMasker Genomic Datasets [73] (last accessed on 7 July 2016). Repeat masking of the Chinese softshell turtle genome was performed with the same procedure as that of the *P. picta* genome.

### Gene prediction

We ran Augustus v3.1 [74] on the *P. picta* genome employing the training module of gene prediction and the hints based on the evidence of transcripts and homologies to peptides of well-annotated species. The detailed procedures are described in Additional file 1: Supplementary Methods. In total, 34,573 genes were predicted. Finally, we selected the genes that had explicit evidence of (1) expression (FPKM ≥ 10) or (2) sequence homology to a protein of any other vertebrates (BLASTP bit-score ≥ 50 for beta-keratins and bit-score ≥ 60 for other genes). The resultant gene set consisted of 27,043 genes, and this was used for the downstream analyses.

### Amniote phylome reconstruction

The detailed procedures of the phylome reconstruction are described in Additional file 1: Supplementary Methods. In summary, employing Ensembl Gene Tree release 82 [75] as seed ortholog groups, we retrieved 13,053 ortholog groups, in which at least one amniote was included, using 20 vertebrates, namely Madagascar ground gecko, Japanese gecko, green anole, Burmese python, garter snake, Chinese softshell turtle, green sea turtle, Mississippi alligator, Chinese alligator, human, dog, opossum, chicken, zebra finch, western clawed frog, coelacanth, gar, zebrafish, stickleback, and sea lamprey. Phylogenetic trees of the 13,043 ortholog groups were inferred with the Maximum-likelihood approach [76].

The inferred gene trees were reconciled with the species tree, whose topology is shown in Fig. 2, to infer the timings of gene origination, duplication, and loss. For this purpose, we iteratively employed the program Notung v2.8 [19]. First, we ran Notung with the ‘rooting’ mode, which provided a root for an unrooted tree to minimize the number of gene duplications and losses. The rooted trees were reformatted by the ETE toolkit [77] to allocate the support values at the correct nodes. For each gene tree, we extracted ten rooted trees in ascending order of the number of rendered duplications and losses, followed by executions of Notung with the ‘rearrange’ mode. This mode allows the bifurcations in the gene trees with low support values to be rearranged to be reconciled with the species tree, resulting in the modification of the gene trees with fewer duplication and loss events. The Notung runs with the ‘rearrange’ mode were performed with different thresholds of bootstrap values (90%, 95%, and 98%). From these runs, the tree topology with the smallest number of gene duplications and losses was selected for each gene group. Finally, Notung with the ‘reconciliation’ mode employing the phylogenomics option was executed using the rearranged trees. The Notung run with this mode counted origination, duplication, and loss events for individual gene trees and summarized these events at every branch of the species tree. Additionally, the execution specified an orthologous/paralogous relationship for each gene pair in an ortholog group in accordance with the information of duplication and loss from a reconciled gene tree.

### Identification of elusive genes and their non-elusive paralogs

The reconciled gene trees were divided into subtrees, each of which contained an ortholog group composed of the bony vertebrates, namely 19 species out of the 20 used for the phylome reconstruction (Additional file 1: Table S7), excluding sea lamprey. The subtrees in a tree are likely to be paralogs occurring in the whole genome duplications in early vertebrates. The orthology information was provided by the Notung outputs. If the subtree included none of the mammalian and avian genes but possessed orthologs of at least two reptile species and at least one outgroup species (amphibians, sarcopterygians, and actinopterygians), we defined the gene as ‘elusive.’ If the reconciled tree including the subtree of the elusive gene also harbored another subtree that retained at least two mammals, two birds, two reptiles, and two outgroups, then the gene in the subtree was defined as ‘non-elusive.’ Among the subtrees of non-elusive genes, the genes closest to the elusive genes based on the comparison of BLASTP bit scores were called representative non-elusive genes and were used in comparative analyses using the elusive genes (Figs. 4–6, Additional file 10: Figure S7, Additional file 11: Figure S8, Additional file 12: Figure S9, Additional file 13: Figure S10).

### Synonymous and non-synonymous substitution rate estimation

Coding nucleotide sequences were aligned in concordance with the alignments of their translation sequences, followed by a removal of ambiguous sites. These procedures were carried out at a time employing trimAl with the options ‘-strict -block 8 -backtrans.’ Numbers of synonymous and non-synonymous substitutions per site were computed with codeml implemented in PAML v4.9a [78].

### Divergence time inference

From the phylome that we constructed, 3735 groups retaining one-to-one orthologous relationships for all tetrapods were retrieved. Among them, we excluded the ortholog groups (1) harboring short alignment lengths and (2) showing anomalous tree shapes, from which the peptide alignment filtered by trimAl contained fewer than 200 ungapped sites, and at least one tree branch leading to an edge belonged to the 0.1% top branch length, or the Robinson–Foulds distance between the gene tree and species tree was more than 0.25. The Robinson–Foulds distance was estimated with the ETE toolkit v3.0 [77]. The resultant 1545 ortholog groups containing 773,242 gapless alignment sites were used for divergence time estimation. We estimated the divergence times with mcmctree implemented in PAML v4.9a [78] allowing separate parameter calculations for each locus assuming the WAG+Γ model. The mcmctree run was performed with a collection of 10,000 samples of every ten generations after discarding 50,000 generations as burn-in. Fossil records for calibration of divergence time estimation are listed in Additional file 1: Table S17.

### Gene expression quantification

The RNA-seq reads from three embryonic stages of *P. picta* [16] and 11 organs/whole embryos of green anole [29] were obtained from the NCBI Short Read Archive. These reads were mapped to the *P. picta* genome assembly and green anole genome assembly AnoCar2.0, respectively, using TopHat v2.1.0 [79], and the mapping data was processed to quantify the gene expression using Cuffdiff v2.2.0 [80]. FPKM values of multiple biological replicates for a sample were averaged. A log ratio of FPKM+1 values was calculated between the elusive genes and their non-elusive paralogs.

### *In situ* hybridization

*P. picta* embryos were staged based on the staging table by Noro et al. [7]. DIG-labeled RNA probes for *P. picta FoxG1* and *FoxG2* were designed to hybridize outside the forkhead domains to avoid cross-hybridization (Additional file 1: Table S18). We performed whole-mount *in situ* hybridization as described previously [10], with minor modifications. Briefly, the step of washing embryos with xylene before rehydration was omitted, and the post-hybridization wash was performed at 65 °C. Additionally, we performed *in situ* hybridization on paraffin-embedded transverse sections in accordance with the previous literature [81], with some modifications as described below. Embryos were fixed in Serra’s fixative, embedded in paraffin, and sectioned at 8 μm. Following deparaffinization with xylene and rehydration with a graded EtOH/PBS series, the sections were fixed in 4% PFA/PBS at room temperature (RT) for 10 min. Hybridization buffer was prepared as follows: 50% formamide, 5× SSC (pH 7.0), 1% SDS, 50 μg/mL total yeast RNA, 50 μg/mL heparin sulfate, 5 mM EDTA (pH 8.0), and 0.1% CHAPS. After hybridization, the sections were washed in washing solution I (50% formamide, 5× SSC, 1% SDS), washing solution II (50% formamide, 2× SSC), washing solution III (2× SSC, 0.3% CHAPS), and washing solution IV (0.2× SSC, 0.3% CHAPS). Individual washing processes with different washing solutions were performed twice for 30 min at 65 °C. Subsequently, the sections were washed in PBST buffer (PBS, 0.3% Tween20) and incubated in blocking solution (0.5% blocking buffer in MABT buffer) for 1 h at RT. Afterward, the sections were incubated with alkaline phosphatase-conjugated anti-DIG Fab fragments (diluted in 1:2000) in 1% blocking reagent in PBST buffer overnight at 4 °C, followed by a wash with TBST for 20 min. For whole-mount *in situ* hybridization of zebrafish, embryos and larvae were fixed overnight at 4 °C in 4% PFA/PBS. The fixed embryos and larvae were washed with PBS and incubated in methanol at −30 °C overnight. The embryos and larvae were rehydrated through incubation in 50% methanol/PBS, in 25% methanol/PBS, and twice in PBST. The samples were treated with proteinase K (10 μg/mL in PBST) for 5 min (for 1 day post fertilization (dpf) embryos), 15 min (for 2 dpf embryos), and 30 min (for 3 dpf larvae) at RT and washed three times with PBST. Hybridization was performed with a digoxigenin-UTP-labeled riboprobe at 65 °C overnight following incubation in hybridization buffer (50% formamide, 5× SSC, 50 μg/mL heparin, 0.1% Tween20, 5 mg/mL torula RNA) for more than 1 h. Subsequently, the samples were washed using three solutions: 50% formamide, 2× SSC, and 0.3% Tween20; 2× SSC and 0.3% Tween20; 0.2× SSC and 0.3% Tween20. The washings with different solutions were individually processed twice for 30 min at 65 °C. The samples were rinsed twice with PBST and incubated with blocking solution (1× Roche blocking reagent, 5% heat-inactivated fetal bovine serum, PBS) for 30 min, followed by further incubation with 1/5000 diluted alkaline phosphatase-conjugated anti-DIG in blocking buffer at RT for 1.5 h. After six washes with PBST for 20 min each and two washes with staining buffer (100 mM Tris-HCl pH 9.5, 50 mM MgCl_2_, 100 mM NaCl, 0.3% Tween20) for 5 min each, the samples were stained with BM purple (Roche) in the dark until staining intensity increased no further. The staining was stopped with three washes of PBS and fixed with 4% PFA/PBS. DIG-labeled RNA probes for zebrafish *foxg1a*, *foxg1b*, *foxg1c*, and *foxg1d* were designed outside the forkhead domains (Additional file 1: Table S18). Plastic sections (10 μm thickness) were made from stained embryos using Technovit 8100 (Heraeus Kulzer) and a Leica RM2125RT microtome.

### Single nucleotide variation (SNV) calling

We employed SNV calling as described previously by Kajitani et al. [34], with some modifications outlined below. The filtered reads for the genome assembly, which contains both paired-ends and singletons, were used for this purpose. Initially, all of these reads were treated as single reads and were mapped to the *P. picta* genome assembly, with the reads exhibiting a more than five edit distance or multi-mapping listed as low-quality. Paired-end reads were also mapped to the *P. picta* genome with Bowtie2 v2.2.9 [82] and SAMTools v1.3.1 [83] keeping the reads in pairs. The low-quality or multi-mapped reads listed were discarded from the paired-end and singleton mapping data using FilterSamReads implemented in Picard Tools v2.4.1 [84]. Additionally, unmapped reads and possible PCR duplicates were removed from the data using ‘samtools view -bh -F 4’ and ‘samtools rmdup,’ respectively. Merging the outputs of the mapping, variants were called with ‘samtools mpileup’ employing a minimum base quality option ‘-Q 30.’ The variants in the 100 bp flanking regions of gaps (Ns) or the 100 bp ends of individual scaffolds were discarded. Finally, the SNVs satisfying ≥ 20 read depth, ≥ 30 mapping quality, and ≥ 25 minor allele frequency were collected using vcffilter implemented in vcflib [85] (accessed 11 June 2016) and vcftools v0.1.14 [86].

### Genome size estimation by flow cytometry

Estimation of nuclear DNA content of *P. picta* was performed as previously described [87], using cells from a whole embryo. Additionally, cells from a chicken embryo [88] with an unknown sex were used as a reference with a known nuclear DNA content. For this purpose, a fertilized chicken egg was obtained from a local farm. The individual embryos were minced using scissors, rinsed once in PBS(−), and incubated in 0.25% trypsin-EDTA solution (Thermo Fisher Scientific) for 15 min at 37 °C with gentle agitation to dissociate the cells. FBS was added to an equal amount to stop the trypsinization reaction. Cell suspension was passed through a 40 μm cell strainer (BD Bioscience) to remove cell clumps and debris. After centrifugation, the cell pellet was rinsed once in PBS(−). Then, 1 × 10^6^ cells in 50 μL of PBS(−) were permeabilized by adding 50 μL of 0.1% Triton-X 100 solution, and DNA staining was performed by adding 450 μL of PI/RNase staining buffer (BD Bioscience) and incubating for 15 min at RT. Fluorescence intensities were measured with the excitation at 488 nm and a bandpass filter of 575/26 nm in FACSCanto II (BD Bioscience). Measurements in triplicates were averaged prior to calculation of nuclear DNA content. According to a regression line passing an origin based on the chicken 2C and 4C peak intensities and their genome sizes, the C-value of *P. picta* was estimated from its 2C peak intensity. The C-value of the *P. picta* genome was converted into genome size, multiplying it by a scale factor of 0.978 × 10^9^ Gb/pg [89] (Additional file 1: Table S3).

### Statistical analyses

All of the statistical tests in this study, which were performed using R, were non-parametric and two-tailed. Mann–Whitney *U* test, Wilcoxon signed-rank test, Fisher’s exact test, the exact test of goodness-of-fit were conducted with wilcox.exact in the exactRankTests package, wilcox.exact with the paired option, and fisher.test and xmulti in the Xnomial package. We considered *p* < 0.05 to be statistically significant. Additionally, effect sizes for the statistical tests were computed.

## Additional files

Additional file 1: Supplementary text, Supplementary methods, Tables S1–S14. **Table S1.** Summary of the genomic DNA sequencing. **Table S2.** Continuity and completeness of the reptile genome assemblies. **Table S3.** Genome size estimation based on flow cytometry. **Table S4.** Repeat content of the Madagascar ground gecko genome assembly. **Table S5.** Predicted genes with homology and transcription evidence. **Table S6.** Hox gene clusters in the *P. picta* genome assembly. **Table S7.** Sources of sets of gene models employed in amniote phylome reconstruction. **Table S9.** One-to-one orthologs in the reconstructed phylome. **Table S11.** Mammalian and Avian species harboring relatively many orthologs of the elusive genes. **Table S12.** Statistical tests for characterization of the elusive genes that retained mammalian and avian orthologs. **Table S13.** Expression levels and evolutionary rates of green anole Rbfox genes. **Table S14.** Numbers of SINEs in the elusive and non-elusive genes and their flanking regions. **Table S15.** Numbers of the orthologs of the elusive genes in the non-mammalian/avian vertebrates. **Table S16.** Conditions of library preparation for genome sequencing. (DOCX 141 kb)

Additional file 2: **Figure S1.** Repeat landscape. (PDF 182 kb)

Additional file 3: **Figure S2.** Difference in GC content across the amniote genomes. (PDF 654 kb)

Additional file 4: **Table S8.** Phylome contents and gene trees. (TXT 45784 kb)

Additional file 5: **Figure S3.** Divergence times between major reptile lineages. (PDF 360 kb)

Additional file 6: **Figure S4.** *K*_S_ distribution of the *P*. *picta* genes. (PDF 210 kb)

Additional file 7: **Figure S5.** Opsin gene tree. (PDF 3021 kb)

Additional file 8: **Figure S6.** Beta-keratin gene tree. (PDF 666 kb)

Additional file 9: **Table S10.** Elusive reptile genes and their non-elusive paralogs. (XLSX 258 kb)

Additional file 10: **Figure S7.** Genomic characters of the elusive and their non-elusive paralogs in the anole genome. (PDF 820 kb)

Additional file 11: **Figure S8.** Gene densities in the 100 kb-flanking regions of the orthologs of the elusive reptile genes and their non-elusive genes. (PDF 1248 kb)

Additional file 12: **Figure S9.** GC content in the genomic regions including the orthologs of the elusive reptile genes and their non-elusive genes. (PDF 1010 kb)

Additional file 13: **Figure S10.** Repeat densities in the genomic regions including the orthologs of the elusive reptile genes and their non-elusive gene. (PDF 739 kb)

Additional file 14: **Figure S11.** Frequency distribution of the elusive genes relating to the number of mammalian and avian orthologs. (PDF 299 kb)

Additional file 15: **Figure S12.** Comparison of expression levels between the elusive genes and non-elusive genes. (PDF 368 kb)

Additional file 16: **Figure S13.** Expression profiles of FoxG genes for zebrafish embryos. (PDF 1046 kb)

Additional file 17: **Figure S14.** SNV density distribution. (PDF 145 kb)

Additional file 18: **Figure S15.** Conserved noncoding elements among amniotes. (PDF 606 kb)

Additional file 19: **Figure S16.** Taste receptor gene tree. (PDF 4710 kb)

Additional file 20: **Figure S17.** Summary of contamination check for genome assembly. (PDF 146 kb)

## Abbreviations

CVG: core vertebrate genes
dpf: days post fertilization
dpo: days post oviposition
FPKM: Fragments per kilobase of transcript per million mapped fragments
MYA: million years ago
myr: million years
RT: room temperature
SMRT: single molecule, real-time
SNV: single nucleotide variation
