# Supplementary material for: Madagascar ground gecko genome analysis characterizes asymmetric fates of duplicated genes
Source: BMC Biol. 2018 Apr 16;16:40. doi: 10.1186/s12915-018-0509-4 (PMC5901865; doi:10.1186/s12915-018-0509-4)
Supplement: Supplementary file 1 — Supplementary text, Supplementary methods, Tables S1–S14. Table S1. Summary of the genomic DNA sequencing. Table S2. Continuity and completeness of the reptile genome assemblies. Table S3. Genome size estimation based on flow cytometry. Table S4. Repeat content of the Madagascar ground gecko genome assembly. Table S5. Predicted genes with homology and transcription evidence. Table S6. Hox gene clusters in the P. picta genome assembly. Table S7. Sources of sets of gene models employed in amniote phylome reconstruction. Table S9. One-to-one orthologs in the reconstructed phylome. Table S11. Mammalian and Avian species harboring relatively many orthologs of the elusive genes. Table S12. Statistical tests for characterization of the elusive genes that retained mammalian and avian orthologs. Table S13. Expression levels and evolutionary rates of green anole Rbfox genes. Table S14. Numbers of SINEs in the elusive and non-elusive genes and their flanking regions. Table S15. Numbers of the orthologs of the elusive genes in the non-mammalian/avian vertebrates. Table S16. Conditions of library preparation for genome sequencing. (DOCX 141 kb) [file 12915_2018_509_MOESM1_ESM.docx]

**Additional file 1: Supplementary text, Supplementary methods, Tables S1-S14**

**Supplementary Text**

*Principal component analysis on the nucleotide base compositions of the amniote genomes*

The Madagascar ground gecko genome exhibited higher GC-content than the lizard and snake genomes while it demonstrated a decreased variance of the GC-content distribution as the genomes of the lizards and snakes did (Additional file 3: Fig. S2a, S2b). In order to uncover the evolution of nucleotide base compositions within order Squamata, a principal component analysis was performed employing the frequencies of 3-mer nucleotides for diverse amniote genomes. The results demonstrated that the GC-content was closely tied to the first principal component (Additional file 3: Fig. S2c). Interestingly, the third principal component, which possessed an explained variance comparable to the second principal component, clearly separated the squamate genomes from the genomes of the other amniotes (Additional file 3: Fig. S2c). The eigenvectors of the third principal component were associated with the presence of CpG dinucleotides. This finding indicates that the genomes of the common squamate ancestors experienced a modification of their base compositions, accompanying an increase of CpG sites and a decrease in the local heterogeneity of GC-content. Later in evolution, the further decrease of the heterogeneity resulted in a greater uniformity of GC-content in the extant anole genomes [[18](#_ENREF_18)].

*The elusive sensory receptor genes exhibiting moderate K_A_ values*

The taste receptor type 1 (Tas1R) gene family, belonging to the G-protein coupled receptor (GPCR) superfamily, includes an elusive gene whose orthologs were identified only in the genomes of the Madagascar ground gecko, green anole, and coelacanth among the 19 examined bony vertebrates (Additional file 19: Fig. S16). The *P*. *picta* and green anole orthologs of the elusive gene harbored comparable *K*_A_ values to those of other paralogs, such as *Tas1R1* and *Tas1R3* (Additional file 1: Table S19). Proteins of this receptor family are known to form heterodimers: in mammals, a TAS1R1/TAS1R3 dimer functions as an 'umami' receptor and a TAS1R2/TAS1R3 dimer forms a sweet receptor [[91](#_ENREF_91)], and various heterodimers of this family also respond to tastants in teleosts [[92](#_ENREF_92)]. This result implies that a unique function of the elusive gene as a component of a heterodimer potentially increases the functional constraint, leading to a decrease in the amino acid substitution rate. Additionally, the opsin gene family, another group of GPCR superfamily, contains a few elusive genes (Additional file 7: Fig. S5). TMT (teleost multiple tissue) opsin, whose orthologs were independently lost in the mammalian and avian lineages, possesses comparable *K*_A_ values to those of its non-elusive paralogs (Additional file 1: Table S19). The Japanese gecko, green anole, sea turtle, and crocodile retain its orthologs, though it is absent from the current *P*. *picta* genome assembly. The TMT opsin orthologs exhibited *K*_A_ and *K*_S_ values comparable to those of the other opsin genes as well, suggesting that this gene is increasing the functional constraint and losing the genomic signatures of the elusive genes (Additional file 1: Table S19). On the other hand, TMT opsin 3, one of the elusive genes within this group, possesses larger *K*_A_ values between the Madagascar ground gecko and green anole orthologs than those of the non-elusive paralogs (Additional file 1: Table S19).

*Conserved non-coding elements (CNEs) in the P. picta genome assembly*

We searched for CNEs in the sauropsid genomes, referring to two different CNE resources: the CONDOR database comprised of non-coding elements conserved between the mammalian and *Fugu rubripes* genomes [[93](#_ENREF_93)] and the elephant shark genome project which included the CNEs between holocephalan and mammalian genomes [[94](#_ENREF_94)] (See Supplementary Methods in this file). Our census incorporating diverse sauropsids revealed that crocodiles retain the largest number of CNEs among the sauropsid species, while squamates have retained the smallest numbers (Additional file 18: Fig. S15). This finding can be explained by the variation of evolutionary rates of CNEs across the taxonomic greoups [[22](#_ENREF_22)]. Importantly, in squamates, many of the CNEs that were failed to be identified with strict criteria were recovered with looser criteria (Additional file 18: Fig. S15). This indicates that the CNEs in squamates have accumulated more nucleotide substitutions than those in the other sauropsids, but still function. The ancestral node splitting chicken and zebra finch, corresponding to the common ancestors of the superorder Neognathae, retains similar number of CNEs as those of the turtles and crocodiles. This result is consistent with the recent findings from the intensive search of the missing genes in the avian genomes: many of the genes that were once considered to be lost in the common ancestor of birds are retained in the avian genomes [[3](#_ENREF_3), [4](#_ENREF_4)]. Therefore, our findings can give the evidence disproving the 'massive gene loss' hypothesis in the early birds.

*Retention of mammalian and avian orthologs by the elusive genes*

We identified the putative orthologs of the 157 elusive genes out of 469 (Additional file 1: Supplementary Methods) from the consolidated sequence set of >100 mammals and >70 birds. The recovery ratio of the orthologs of the elusive genes in the mammalian and avian genomes (33.5%) was much less than that in the previous study in the avian genomes (86.9%) for the ortholog groups consisting of human and Chinese softshell turtle [[4](#_ENREF_4)]. Additionally, the frequency distributions of these genes relating to a number of species retaining orthologs were notably different between the two studies. Our study revealed that 40.4% and 8.5% of the genes which retained avian orthologs included ≥10 and ≥40 bird species, respectively [[4](#_ENREF_4)], while the analyses performed by Botero-Castro et al. revealed that 75.5% and 51.5% of the genes with avian orthologs included such numbers of the birds (Fig. S11). The finding may be either explained by frequent loss of the elusive genes in birds or failure of the ortholog detection potentially due to extremely high GC-content in the genomic regions.

Our consolidated sequence set for the ortholog detection included the genome-wide gene sets of chicken and golden collared manakin (*Manacus vitellinus*) and full-length transcriptome catalogs of chicken, zebra finch, ruby-throated hummingbird (*Archilochus colubris*), and Anna’s hummingbird (*Calypte anna*) that were produced utilizing the SMRT sequencing platform. These assemblies were expected to recover the orthologs located in the genomic regions that were excluded from the genome assembly (e.g., GC-rich regions) employing short reads. However, the set of gene models of the chicken was already employed for the initial search for the elusive genes, and the gene model sets of the golden collared manakin and the full-length transcriptome catalogs of the four birds recovered the orthologs of 21 or less elusive genes (Table S11). The results imply that the absence of the orthologs of the elusive genes from the avian genome assemblies is not associated with the genomic features relating to difficulty in sequencing of the corresponding regions. Rather, the results may reflect less frequent retention of avian orthologs for the elusive genes than that for the genes used by Botero-Castro et al.

**Supplementary Methods**

*Removal of contaminant sequences*

Following the genome assembly, the scaffold sequences were screened for contaminated organismal and artificial sequences. DeconSeq version v0.4.3 [[67](#_ENREF_67)] was executed for the scaffolds using potential contaminant sequences such as *P. picta* mitochondrial DNA and the phiX spike-in sequence, as well as 2,513 bacterial genome and 21 fungal genome sequences obtained from the Microbial Genome Database (MBGD) [[95](#_ENREF_95)]. Additionally, we searched for contaminants derived from the same sequencing runs as the *P*. *picta* genomic DNA sequencing. For this purpose, the in-house genome and transcriptome assemblies whose reads were produced in the same lanes of the Illumina HiSeq flow cell as the *P*. *picta* genome sequencing were also utilized as the reference of potential contaminants. The DeconSeq run was executed by querying the scaffolds split into 1 kb windows with 100 bp overlaps. The split sequences that exhibited ≥90 bp matches and ≥98% similarities to the potential contaminants were retrieved as candidates of contamination. These similarity matches were validated with BLASTN implemented in NCBI BLAST 2.2.31+ [[96](#_ENREF_96)]. From these candidates, we ignored those harboring the similarity with the vertebrate ultraconserved elements that were obtained from http://urtraconserved.org [[97](#_ENREF_97)] and UCNEbase [[98](#_ENREF_98)], as well as those that included simple repeats and low-complexity regions predicted by RepeatMasker v4.0.5 [[72](#_ENREF_72)] with ≥50% of their lengths. Furthermore, if the candidates matched the potential contaminant but also overlapped with *P. picta* *de novo* transcriptome contigs [[16](#_ENREF_16)] with a high expression level (FPKM≥5), then they were removed from the list of candidates. Following this filtering of the contaminant candidates of the split scaffolds, we searched for the full-length scaffolds that were found to have ≥95% of their entire length composed of these candidates. If the split sequences of these scaffolds exhibited ≥98% similarities with the potential contaminants on average, the scaffolds were finally classified as contaminants. This procedure resulted in the elimination of a total of 21 full scaffolds from the original assembly. A flowchart of this procedure is shown in Additional file 20: Fig. S17. Finally, scaffold IDs were re-assigned according to the sequence length.

*Gene prediction*

In order to predict a set of gene model, we ran Augustus v3.1 [[74](#_ENREF_74)] on the *P*. *picta* genome employing the training module of gene prediction and the hints based on the evidence of transcripts and sequence homologies to peptides of the well-annotated vertebrate species. Within the genome assembly, 336 genes were reconstructed in full lengths using the BUSCO v1.1 pipeline referring to its metazoan reference gene set [[99](#_ENREF_99)]. These genes were used to train the program Augustus in order to set the optimal gene prediction parameters. Exon boundary hints were extracted from the mapping data of the *P*. *picta* embryonic RNA-seq reads, which had been produced in our previous study [[16](#_ENREF_16)], to the genome assembly using Tophat v2.1.0 [[79](#_ENREF_79)]. Additionally, hints of exon boundaries and coding regions were extracted from the alignments between the human and chicken RefSeq proteins and the *P*. *picta* genome assembly, employing Exonerate 2.2.0 [[100](#_ENREF_100)]. Augustus was executed with the options '--codingseq=1 --protein=1 --alternatives-from-evidence=false --allow_hinted_splicesites=atac --introns=on --softmasking=1 --genemodel=partial' using the aforementioned masked genome assembly processed by RepeatMasker. The Augustus run resulted in 34,593 predicted protein-coding genes.

The gene models predicted by Augustus were further improved by utilizing the transcription evidence and sequence homology to known proteins. This improvement that included splitting fusion genes into multiple genes, fusing multiple split genes into one, and elongating truncated genes were processed as described previously [[101](#_ENREF_101)] with some modifications. The mapping-origin transcripts were generated from the RNA-seq read mapping data using Cufflinks v2.2.0 [[80](#_ENREF_80)]. We also utilized the *de novo* transcriptome assembly, which was previously reconstructed from the same RNA-seq reads as the mapping-origin method [[16](#_ENREF_16)], followed by alignment of them to the genome assembly with PASA v2.0.2 [[102](#_ENREF_102)]. We employed the protein-genome alignments that were previously utilized to provide hints for the Augustus gene prediction as protein-homology evidence. The overlapping rule between the aligned positions with the peptides and the locations of the transcript evidence in the genome assembly was modified from ≥60% to ≥80%. This procedure produced 34,463 protein-coding genes.

Separately, beta-keratin genes were specifically re-annotated employing the Augustus Protein Profile eXtension (PPX) method [[103](#_ENREF_103)]. Amino acid sequences of birds and reptile beta-keratin, which were retrieved from the NCBI RefSeq database, were aligned with MAFFT v7.222 [[104](#_ENREF_104)], followed by classification of the alignment into one of four groups in accordance with their sequence similarity. For the individual groups, protein profiles were generated using the script msa2prfl.pl implemented in the Augustus package. Augustus runs were performed employing these protein profiles, resulting in the prediction of 120 genes whose translated sequences harbored the hits against at least one of the protein profiles. Of them, 110 genes were unique to this gene prediction. These procedures resulted in 34,573 predicted genes. The genes were filtered by transcriptome evidence and sequence similarities with vertebrate peptides, resulting in 27,043 genes (See Methods).

*Retrieval of ortholog groups and phylogenetic tree inference for phylome reconstruction*

In order to reconstruct the amniote phylome, we first made core homolog groups based on Ensembl Gene Tree release 82 [[75](#_ENREF_75)]. From the individual gene trees provided by this repository, we extracted the subtrees that had the largest tree space containing only vertebrates. From the subtrees, we retrieved the core homologs, consisting of the amino acid sequences of 13 vertebrates: human, dog, opossum, chicken, zebra finch, Chinese softshell turtle, green anole, western clawed frog, coelacanth, gar, zebrafish, stickleback, and sea lamprey. Individual peptides of the homolog groups were translations from the 'canonical' transcripts, each representing a gene from the multiple transcripts in the Ensembl gene annotations. Next, we added putative orthologs of Madagascar ground gecko, Japanese gecko, Burmese python, garter snake, green sea turtle, Mississippi alligator, and Chinese alligator into the core homolog groups. For this purpose, the peptides of these reptiles except Madagascar ground gecko were retrieved from NCBI RefSeq [[105](#_ENREF_105)], and BLASTP (in NCBI BLAST v2.2.31+ [[96](#_ENREF_96)]) runs were carried out for the peptides of the seven reptiles using those of the 13 aforementioned vertebrates as a database. Within the peptide sets of these reptiles, we selected the 'canonical' peptides of the individual genes whose BLASTP bit scores were the largest among the peptides derived from a gene. The canonical peptides were integrated into the core homolog groups when satisfying both of the two following conditions: (i) the canonical peptide had a BLASTP best-hit to a member of the core homolog group and overlapped with ≥50% of its length; (ii) the best-hit bit score between them was greater than at least one of the bit scores between two of the members of the core homolog group.

In this procedure, we integrated the canonical RefSeq peptides of chicken, zebra finch, Chinese softshell turtle, and green anole that were absent from the Ensembl annotations. The loci that were annotated by RefSeq but not by Ensembl were extracted by comparing the annotation between RefSeq and Ensembl for all of the species except chicken, whose release version of the genome assemblies was different between the two databases. The chicken canonical transcripts from RefSeq and Ensembl were clustered by cd-hit-est v4.6.4 [[106](#_ENREF_106)] with the options '-c 0.95 -G 0 -aL 0.3 -aS 0.5' followed by extraction of the representative sequences. When the RefSeq protein was predicted more completely than the Ensembl canonical peptide in the same loci, the canonical peptide was replaced by this RefSeq protein. Through these procedures, 12,598 homolog groups including at least one genes of amniote species were produced.

Additionally, we reconstructed homolog groups that originated in the sauropsid lineages but were absent from the Ensemble Gene Tree. Canonical peptides of singletons from the 20 mentioned vertebrates, which did not belong to any of the core homolog groups, were clustered with OrthoFinder version 0.4 [[107](#_ENREF_107)] with its default parameters. In the individual clusters, the peptides with at least one BLASTP hit to other members overlapping with ≥50% of their lengths were selected. Finally, 455 clusters that consisted of only the selected members of sauropsids were used as homolog groups, and 13,053 groups were produced in total.

Amino acid sequence alignments of these 13,053 ortholog groups were produced with the six-way alignment method introduced in the PhylomeDB construction [[108](#_ENREF_108)]. Forward and reverse sequence alignments were performed with MAFFT v7.222 [[104](#_ENREF_104)], Clustal Omega v1.2.0 [[109](#_ENREF_109)], and ProbCons v1.12 [[110](#_ENREF_110)], and the consensus alignments were produced with M-Coffee v11.00 [[111](#_ENREF_111)]. Unambiguous sites in the alignments were selected with trimAl v1.4 [[112](#_ENREF_112)] with the '-automated1' option. If the number of ungapped sites in the trimmed alignment was no less than 200 and more than half of the total sites, the gapped sites were discarded. Using the alignments of the 11,581 groups containing four or more sequences, phylogenetic trees were inferred with IQ-TREE v1.4.0-beta2 [[76](#_ENREF_76)] employing the substitution model selection and Ultrafast bootstrap methods [[113](#_ENREF_113)]. Of these, 11,571 gene trees were inferred. Tree topologies were given *a priori* for the ortholog groups consisting of two or three sequences.

*Search for mammalian and avian putative orthologs of the elusive genes*

In order to detect mammalian and avian orthologs of the elusive genes, we constructed peptide and nucleotide sequence databases of mammals and aves, which mainly consisted of the gene models based on the whole genome assembly and large-scale transcriptome sequence sets. For this purpose, we first retrieved the NCBI nr ant nt sequence collections of mammals and birds, which included the genome-wide gene sets of 112 mammals and 68 birds, as well as the *de novo* transcriptome assemblies, the Iso-seq full length transcripts, and EST sequences of 66 mammals and 19 birds from the NCBI Transcriptome Shotgun Assembly (TSA) and NCBI EST. The Iso-seq full length transcripts of ruby-throated hummingbird were obtained from the Zenodo repository (https://zenodo.org/record/311651) [[114](#_ENREF_114)], and those of zebra finch and Anna’s hummingbirds were downloaded from the following URL (https://downloads.pacbcloud.com/public/dataset/AvianBrainTranscriptome/) [[115](#_ENREF_115)]. Furthermore, as performed in the previous literature [[4](#_ENREF_4)], we reconstructed *de novo* transcriptome assemblies of 43 birds. From NCBI Short Read Archive (SRA), we downloaded short reads that were listed in Supplementary Table 1 of Botero-Castro et al. 2017 [[4](#_ENREF_4)], except those of *Melopsittacus undulatus* (SRR029329, SRR029330, SRR5336227, SRR5336228, SRR5336543, SRR5336544, SRR5336545, SRR5336546, SRR5336547). The reads were pooled for each species and subject to *de novo* transcriptome assembly with Trinity v2.4.0 following adapter and quality trimming with trim_galore v0.4.3 filter (options ‘--phred33 -q 30 --length 50 --trim1’ for the reads 75 nt or longer and ‘--phred33 -q 30 --length 30 --trim1’ for those of 50 nt or shorter) and quality filtering with fastq_quality (options ‘-Q 33 -q 30 -p 80’) embedded by FASTX-toolkit v0.0.14. The contigs of the *de novo* assemblies and EST sequences were grouped for each species based on nucleotide sequence similarity with cd-hit-est v4.6.8 with options ‘-c 0.95 -n 10,’ and the representative sequences for each cluster were used for the following analyses. In total we retrieved the comprehensive transcriptome evidence of 56 birds.

The reptile elusive genes were queried against the peptides and nucleotides described above with NCBI blastp and tblastn, respectively (the first search). For each query, we retrieved the subjects whose bit scores exceeded the half of that of the best-hit. This search was expected to contain more subjects than the best-hit criterion and retrieve the truncated sequences of true orthologs, which potentially exhibit lower bit scores than the best-hit. Subsequently, these subjects, the mammalian and avian homologs, were queried against the peptides of the reptiles used in the study employing NCBI blastp and blastx (the second search). If the best-hits of the second search were identical to the queries of the first search or belonged to the same ortholog groups as these queries, the mammalian and avian homologs were considered to be the candidates of the orthologs. The peptide sequences of the candidates were further queried against the non-amniote orthologs of the elusive genes in order to exclude falsely collected candidates based on the following criteria (the thirds search). In accordance with the cascade of the similarity searches, we selected the candidates that satisfied the following conditions (i) or (ii) as putative orthologs: (i) *Bit*_e,c_ > *Bit*_c,o_; (ii) *Bit*_e,c_ > *Bit*_c,o_×0.8 and *Sim*_e,c_ > *Sim*_c,o_; where *Bit*_e,c_ denotes a bit score based on the amino acid substitution matrix between an elusive gene and its candidate of mammalian or avian ortholog in the first search, *Bit*_c,o_ indicates the one between the candidate and its best-hit non-amniote ortholog of the elusive gene in the third search, and *Sim*_e,c_ and *Sim*_c,o_ represent the percent similarities based on the BLAST searches for these pairs. In order to achieve more sensitive search for mammalian and avian orthologs, we also employed MMSeqs2 [[116](#_ENREF_116)] with the option of two iterations in the same procedure as performed above with BLAST. This search identified no additional mammalian or avian species that retained the orthologs of the elusive genes.

*Conserved noncoding elements (CNEs)*

The conserved noncoding elements in the human genome were retrieved from the CONDOR database [[93](#_ENREF_93)] in which the CNEs were identified by comparisons between various mammalian genomes and the *Fugu rubripes* genomes. In addition, the conserved elements in the elephant shark genome that were identified previously were retrieved [[94](#_ENREF_94)]. We divided the BLASTN top hits of these CNEs within each individual genome of the 14 amniotes into the three classes that follow: a strict match with ≥100 bp aligned regions and >95% similarity, a moderate match with ≥80 bp aligned regions and >85% similarity, and a relaxed match with ≥60 bp aligned regions and >80% similarity.

**Supplementary Tables**

**Table S1. Summary of the genomic DNA sequencing**

| SRA Run ID | Insert size/ target mate distance (bp/kb) | Sequencer | Read length (nt) | Number of raw reads | Number of pairs after QC/NextClip | Number of bases in pairs after QC/NextClip | Number of singletons after QC/NextClip | Number of bases used including single reads after QC | Average Sequence depth |
| --- | --- | --- | --- | --- | --- | --- | --- | --- | --- |
| Paired-end libraries | |  |  |  |  |  |  |  |  |
| DRR089867 | 380 | HiSeq 1500 | 151 | 91,195,345 | 87,451,440 | 25,638,345,307 | 3,038,676 | 26,059,038,484 |  |
| DRR089868 | 480 | HiSeq 1500 | 151 | 107,862,502 | 101,518,039 | 29,578,911,146 | 5,491,173 | 30,338,209,877 |  |
| DRR089869 | 610 | HiSeq 1500 | 151 | 79,822,580 | 72,136,369 | 20,796,851,706 | 6,968,544 | 21,784,022,146 |  |
| DRR089870 | 380 | HiSeq 1500 | 171 | 63,540,896 | 61,549,881 | 20,442,724,801 | 1,190,565 | 20,626,493,679 |  |
| DRR089871 | 480 | HiSeq 1500 | 171 | 57,643,086 | 55,217,515 | 18,316,958,148 | 1,741,173 | 18,589,052,339 |  |
| DRR089872 | 610 | HiSeq 1500 | 171 | 21,545,432 | 19,979,851 | 6,569,894,994 | 1,295,543 | 6,777,534,306 |  |
| DRR089873 | 610 | MiSeq | 301 | 22,525,496 | 21,191,530 | 11,653,772,284 | 438,101 | 11,774,343,663 |  |
| Paired-end total | |  |  | 444,135,337 | 419,044,625 | 132,997,458,386 | 20,163,775 | 135,948,694,494 | 75.4 |
|  |  |  |  |  |  |  |  |  |  |
| Mate-pair libraries | |  |  |  |  |  |  |  |  |
| DRR089874 | 1-6 | MiSeq | 301 | 5,180,605 | 3,799,955 | 1,297,157,832 |  |  |  |
| DRR089875 | 1-6 | HiSeq 1500 | 171 | 45,684,998 | 27,358,190 | 6,648,034,972 |  |  |  |
| DRR089876 | 1-6 | Miseq | 301 | 6,743,037 | 5,035,179 | 1,569,172,780 |  |  |  |
| DRR089877 | 1-6 | HiSeq 1500 | 171 | 22,344,855 | 14,616,403 | 3,468,713,254 |  |  |  |
| DRR089878 | 3-8 | Miseq | 301 | 5,516,267 | 4,188,410 | 1,324,704,763 |  |  |  |
| DRR089879 | 3-8 | HiSeq 1500 | 171 | 40,038,491 | 26,292,846 | 6,286,799,194 |  |  |  |
| DRR089880 | 6-10 | Miseq | 301 | 4,708,934 | 3,647,341 | 1,227,918,021 |  |  |  |
| DRR089881 | 6-10 | HiSeq 1500 | 171 | 21,085,361 | 13,884,252 | 3,443,513,964 |  |  |  |
| DRR089882 | 11-18 | Miseq | 301 | 3,203,603 | 2,448,025 | 772,074,997 |  |  |  |
| DRR089883 | 11-18 | HiSeq 1500 | 171 | 19,833,527 | 13,513,880 | 3,288,172,552 |  |  |  |
| Mate-pair total | |  |  | 174,339,678 | 114,784,481 | 29,326,262,329 |  |  |  |

**Table S2. Continuity and completeness of the reptile genome assemblies**

| Species | Common name | Family | Genome assembly size | CEGMA+CVG | | | BUSCO+vBUSCO | | | N50 scaffold length (bp) | Longest scaffold (bp) |
| --- | --- | --- | --- | --- | --- | --- | --- | --- | --- | --- | --- |
|  |  |  |  | %Complete | %Partial | %1-to-1 Complete | %Complete | %Partial | %1-to-1 Complete |  |  |
| *Paroedura picta* | Madagascar ground gecko | Gekkonidae | 1,694,174,484 | 89.70 | 98.71 | 95.22 | 89.79 | 96.75 | 99.22 | 4,106,116 | 33,658,631 |
| *Gekko japonicus* | Japanese gecko | Gekkonidae | 2,490,257,917 | 80.69 | 97.85 | 87.23 | 90.84 | 97.10 | 98.81 | 707,733 | 4,762,499 |
| *Eublepharis macularius* | leopard gecko | Eublepharidae | 2,017,070,936 | 90.56 | 97.85 | 91.00 | 95.13 | 98.45 | 98.78 | 663,762 | 10,696,998 |
| *Anolis carolinensis* | green anole | Iguanidae | 1,931,078,847 | 88.41 | 98.71 | 97.57 | 88.82 | 94.47 | 99.13 | 150,641,573 | 263,920,458 |
| *Pogona vitticeps* | Central bearded lizard | Agamidae | 1,816,116,151 | 88.84 | 97.85 | 94.20 | 94.86 | 97.95 | 99.39 | 2,290,546 | 14,681,335 |
| *Protobothrops mucrosquamatus* | brown spotted pit viper | Viperidae | 1,673,860,670 | 89.27 | 98.71 | 90.38 | 91.88 | 97.53 | 98.02 | 424,052 | 6,916,597 |
| *Python bivittatus* | Burmese python | Pythonidae | 1,435,034,535 | 84.55 | 97.42 | 88.32 | 91.69 | 97.10 | 99.28 | 213,970 | 1,452,584 |
| *Ophiophagus hannah* | king cobra | Elapidae | 1,594,074,654 | 81.97 | 97.42 | 85.86 | 87.20 | 95.82 | 99.33 | 241,519 | 2,844,733 |
| *Thamnophis sirtalis* | garter snake | Colubridae | 1,424,897,867 | 63.52 | 90.13 | 93.24 | 72.31 | 87.01 | 98.66 | 647,592 | 3,920,154 |
| *Ophisaurus gracilis* | Burmese glass lizard | Anguidae | 1,781,357,942 | 90.99 | 98.71 | 95.75 | 95.44 | 98.26 | 99.23 | 1,273,270 | 6,684,035 |
|  |  |  |  |  |  |  |  |  |  |  |  |
| *Pelodiscus sinensis* | Chinese softshell trutle | Trionychidae | 2,202,483,752 | 81.55 | 97.85 | 95.79 | 94.04 | 97.83 | 99.42 | 3,350,749 | 16,024,077 |
| *Chelonia mydas* | green sea turtle | Cheloniidae | 2,208,393,880 | 86.70 | 98.71 | 96.04 | 94.66 | 98.41 | 99.14 | 3,864,108 | 90,216,835 |
| *Chrysemys picta bellii* | western painted turtle | Emydidae | 2,365,766,571 | 85.41 | 99.14 | 98.99 | 95.90 | 98.45 | 99.27 | 7,072,151 | 77,392,008 |
| *Alligator mississippiensis* | American alligator | Alligatoridae | 2,174,243,242 | 83.69 | 98.28 | 93.85 | 95.55 | 98.65 | 99.39 | 508,966 | 4,958,242 |
| *Alligator sinensis* | Chinese alligator | Alligatoridae | 2,274,864,441 | 86.70 | 99.14 | 98.02 | 95.05 | 98.22 | 99.35 | 2,188,296 | 8,641,424 |
| *Crocodylus porosus* | saltwater crocodile | Crocodylidae | 2,123,474,087 | 79.83 | 97.85 | 89.25 | 92.07 | 98.18 | 98.95 | 204,986 | 2,119,207 |
| *Gavialis gangeticus* | gharial | Gavialidae | 2,882,656,219 | 64.38 | 97.00 | 91.33 | 84.57 | 96.71 | 99.36 | 127,604 | 1,020,052 |

**Table S3. Genome size estimation based on flow cytometory**

| Species | Known DNA amount (pg) | Samples/ average/ SD | PI PE-A (×10^3^) | | Estimated C-value (pg)^*1^ | Estimated genome size (Gb)^*2^ |
| --- | --- | --- | --- | --- | --- | --- |
|  |  |  | 2N | 4N |  |  |
| Chicken | 1.25 | embryo-1 | 29.53 | 57.03 | 1.312 | 1.284 |
|  |  | embryo-2 | 27.9 | 55.28 | 1.240 | 1.213 |
|  |  | embryo-3 | 28.5 | 55.83 | 1.267 | 1.239 |
|  |  | average | 28.64 | 56.04 | 1.273 | 1.245 |
|  |  | SD | 0.82 | 0.9 | 0.036 | 0.036 |
| Madagascar ground gecko |  | embryo-1 | 41.15 | 82.58 | 1.829 | 1.789 |
|  |  | embryo-2 | 41.46 | 82.68 | 1.843 | 1.802 |
|  |  | embryo-3 | 41.77 | 83.17 | 1.856 | 1.816 |
|  |  | average | 41.46 | 82.81 | 1.843 | 1.802 |
|  |  | SD | 0.31 | 0.31 | 0.014 | 0.013 |

^*1^ The regression line passing an origin between the DNA amount of the chicken haplotype and the PI PE-A peak values was approximated as y = 0.00225x. Then the C-values were calculated from the PI PE-A peak values for 2N employing this regression line.

^*2^ The genome size was computed converted by multiplying the C-value by a scale factor 0.978×10^9^ Gb/pg

**Table S4. Repeat content of the Madagascar ground gecko genome assembly**

| Class | Family | Count | Masked bp | %masked |
| --- | --- | --- | --- | --- |
| DNA | CMC-EnSpm | 3,821 | 346,696 | 0.02 |
|  | PIF-Harbinger | 73,201 | 7,457,038 | 0.44 |
|  | TcMar-Mariner | 875 | 235,059 | 0.01 |
|  | TcMar-Tc2 | 14,693 | 2,432,674 | 0.14 |
|  | TcMar-Tigger | 417 | 103,829 | 0.01 |
|  | hAT-Ac | 71,509 | 9,073,403 | 0.54 |
|  | hAT-Blackjack | 70,105 | 7,972,491 | 0.47 |
|  | hAT-Charlie | 179,832 | 36,077,824 | 2.13 |
|  | hAT-Tag1 | 9,271 | 1,475,195 | 0.09 |
|  | hAT-Tip100 | 19,483 | 2,612,971 | 0.15 |
| LINE | CR1 | 382,195 | 79,468,587 | 4.70 |
|  | CRE | 7,454 | 709,559 | 0.04 |
|  | Dong-R4 | 1,589 | 498,361 | 0.03 |
|  | Jockey | 3,037 | 779,729 | 0.05 |
|  | L1 | 18,075 | 5,960,751 | 0.35 |
|  | L2 | 353,857 | 78,159,778 | 4.62 |
|  | Penelope | 170,107 | 21,725,026 | 1.28 |
|  | RTE-BovB | 131,281 | 31,155,432 | 1.84 |
|  | RTE-X | 29,569 | 8,378,412 | 0.50 |
| LTR | Copia | 1,946 | 774,778 | 0.05 |
|  | DIRS | 8,573 | 2,814,205 | 0.17 |
|  | ERV4 | 1,464 | 401,691 | 0.02 |
|  | Ginger | 1,337 | 622,087 | 0.04 |
|  | Gypsy | 8,846 | 4,770,531 | 0.28 |
|  | Ngaro | 4,734 | 2,918,718 | 0.17 |
|  | (Unannotated) | 1,208 | 433,739 | 0.03 |
| RC | Helitron | 582 | 78,674 | <0.01 |
| SINE | 5S | 146,952 | 20,861,956 | 1.23 |
|  | 5S-Deu-L2 | 21,497 | 2,531,607 | 0.15 |
|  | ID | 64,655 | 9,154,161 | 0.54 |
|  | L2 | 3,345 | 273,077 | 0.02 |
|  | MIR | 355,875 | 61,321,839 | 3.63 |
|  | U | 387 | 13,030 | <0.01 |
|  | tRNA-Core-RTE | 2,829 | 290,278 | 0.02 |
|  | tRNA-Sauria | 2,084 | 212,343 | 0.01 |
|  | tRNA-Sauria-L2 | 46,669 | 5,118,684 | 0.30 |
|  | (Unannotated) | 2,154 | 195,723 | 0.01 |
| Unknown |  | 1,107,946 | 204,012,208 | 12.06 |
| total interspersed |  | 3,323,454 | 611,422,144 | 36.15 |
|  |  |  |  |  |
| Low_complexity |  | 27,048 | 1,372,758 | 0.08 |
| RNA |  | 628 | 97,112 | 0.01 |
| Satellite |  | 15,943 | 3,601,365 | 0.21 |
| Simple_repeat |  | 323,429 | 14,646,606 | 0.87 |
| rRNA |  | 12,398 | 422,558 | 0.02 |
| snRNA |  | 889 | 35,789 | <0.01 |
| Total |  | 3,703,789 | 631,598,332 | 37.34 |

**Table S5. Predicted genes with homology and transcription evidence**

| Total number of genes predicted by Augustus | 34,573 |
| --- | --- |
| (i) Genes with homology evidence | 26,432 |
| (ii) Genes with transcription evidence | 16,938 |
| Final gene set (satisfying either (i) or (ii)) | 27,039 |

**Table S6. Hox gene clusters in the *P. picta* genome assembly**

| Classification | Location | Members |
| --- | --- | --- |
| HoxA | scaffold00000026: 8.64-8.76 Mb | *HoxA1*, *HoxA2*, *HoxA3*, *HoxA4*, *HoxA5*, *HoxA6*, *HoxA7*, *HoxA9*, *HoxA10*, *HoxA11*, *HoxA13* |
| HoxB | scaffold00000298: 0.13-0.42 Mb | *HoxB1*, *HoxB2*, *HoxB3*, *HoxB4*, *HoxB6*, *HoxB7*, *HoxB8*, *HoxB9*, *HoxB13* |
| HoxC | scaffold00000132: 1.81-2.03 Mb | *HoxC3*, *HoxC4*, *HoxC6*, *HoxC8*, *HoxC9*, *HoxC10*, *HoxC11*, *HoxC12*, *HoxC13* |
| HoxD | scaffold00000146: 0.89-1.03 Mb | *HoxD1*, *HoxD3*, *HoxD4*, *HoxD8*, *HoxD9*, *HoxD10*, *HoxD12*, *HoxD13* |

**Table S7. Sources of sets of gene models employed in our the amniote phylome reconstruction**

| Species | Species abbreviation | Common name | Biodata repository | Genome assembly release | Annotation release |
| --- | --- | --- | --- | --- | --- |
| *Homo sapiens* | HOMSA | human | Ensembl | GRCh38.p3 | Release 82 |
| *Canis familiaris* | CANFA | dog | Ensembl | CanFam3.1 | Release 82 |
| *Monodelphis domestica* | MONDO | opossum | Ensembl | monDom5 | Release 82 |
| *Gallus gallus* | GALGA | chicken | Ensembl | Galgal4 | Release 82 |
|  |  |  | NCBI RefSeq Genome | Gallus_gallus-5.0 | NCBI Gallus gallus Annotation Release 103 |
| *Taeniopygia guttata* | TAEGU | zebra finch | Ensembl | taeGut3.2.4 | Release 82 |
|  |  |  | NCBI RefSeq Genome | Taeniopygia_guttata-3.2.4 | NCBI Taeniopygia guttata Annotation Release 103 |
| *Alligator mississippiensis* | ALIMI | American alligator | NCBI RefSeq Genome | ASM28112v3 | NCBI Alligator mississippiensis Annotation Release 101 |
| *Alligator sinensis* | ALISI | Chinese alligator | NCBI RefSeq Genome | ASM45574v1 | NCBI Alligator sinensis Annotation Release 101 |
| *Pelodiscus sinensis* | PELSI | Chinese softshell tuｒtle | Ensembl | PelSin_1.0 | Release 82 |
|  |  |  | NCBI RefSeq Genome | PelSin_1.0 | NCBI Pelodiscus sinensis Annotation Release 101 |
| *Chelonia mydas* | CHEMY | green sea turtle | NCBI RefSeq Genome | CheMyd_1.0 | NCBI Chelonia mydas Annotation Release 100 |
| *Anolis carolinensis* | ANOCA | green anole | Ensembl | AnoCar2.0 | Release 82 |
|  |  |  | NCBI RefSeq Genome | AnoCar2.0 | NCBI Anolis carolinensis Annotation Release 101 |
| *Python bivittatus* | PYTBI | Burmese python | NCBI RefSeq Genome | Python_molurus_bivittatus-5.0.2 | NCBI Python bivittatus Annotation Release 100 |
| *Thamnophis sirtalis* | THASI | garter snake | NCBI RefSeq Genome | Thamnophis_sirtalis-6.0 | NCBI Thamnophis sirtalis Annotation Release 100 |
| *Gekko japonicus* | GEKJA | Japanese gecko | NCBI RefSeq Genome | Gekko_japonicus_V1.1 | NCBI Gekko japonicus Annotation Release 100 |
| *Xenopus tropicalis* | XENTR | Western clawed frog | Ensembl | JGI 4.2 | Release 82 |
| *Latimeria chalumnae* | LATCH | coelacanth | Ensembl | LatCha1 | Release 82 |
| *Lepisosteus oculatus* | LEPOC | spotted gar | Ensembl | LepOcu1 | Release 82 |
| *Danio rerio* | DANRE | zebrafish | Ensembl | GRCz10 | Release 82 |
| *Gasterosteus aculeatus* | GASAC | stickleback | Ensembl | BROAD S1 | Release 82 |
| *Petromyzon marinus* | PETMA | sea lamprey | Ensembl | Pmarinus_7.0 | Release 82 |

**Table S9. One-to-one orthologs in the reconstructed phylome**

| Taxonomic group | Number of ortholog groups |
| --- | --- |
| Gekkonidae | 12,889 |
| Madagascar ground gecko-green anole | 12,330 |
| Squamates | 10,585 |
| Sauropsids | 6,162 |
| Amniotes | 4,935 |
| Tetrapods | 3,749 |
| Euteleost | 1,919 |
| Vertebrates | 816 |

**Table S11. Mammalian and Avian species harboring relatively many orthologs of the elusive genes**

| Species | Number of orthologs of the elusive genes | Taxonomy | SMRT for genome/transcriptome sequencing |
| --- | --- | --- | --- |
| Birds (≥20 elusive genes) |  |  |  |
| *Struthio camelus* | 48 | Palaeognathae |  |
| *Apteryx australis* | 47 | Palaeognathae |  |
| *Aegypius monachus* | 36 | Palaeognathae |  |
| *Anas platyrhynchos* | 34 | Neognathae |  |
| *Aquila chrysaetos* | 34 | Palaeognathae |  |
| *Circus melanoleucos* | 33 | Palaeognathae |  |
| *Asio otus* | 31 | Neognathae |  |
| *Accipiter virgatus* | 30 | Palaeognathae |  |
| *Butastur indicus* | 30 | Palaeognathae |  |
| *Calidris pugnax* | 30 | Neognathae |  |
| *Elanus caeruleus* | 30 | Palaeognathae |  |
| *Athene noctua* | 28 | Neognathae |  |
| *Haliaeetus leucocephalus* | 28 | Palaeognathae |  |
| *Philomachus pugnax* | 27 | Neognathae |  |
| *Nipponia nippon* | 26 | Neognathae |  |
| *Patagioenas fasciata* | 25 | Neognathae |  |
| *Falco peregrinus* | 24 | Neognathae |  |
| *Otus scops* | 24 | Neognathae |  |
| *Aptenodytes forsteri* | 23 | Neognathae |  |
| *Bubo bubo* | 23 | Neognathae |  |
| *Charadrius vociferus* | 23 | Neognathae |  |
| *Columba livia* | 23 | Neognathae |  |
| *Anser cygnoides* | 22 | Neognathae |  |
| *Pygoscelis adeliae* | 22 | Neognathae |  |
| *Manacus vitellinus* | 21 | Neognathae | genome |
| *Buteo buteo* | 20 | Palaeognathae |  |
| Mammals (≥10 elusive genes) |  |  |  |
| *Ornithorhynchus anatinus* | 30 | Prototheria |  |
| *Phascolarctos cinereus* | 16 | Metatheria | genome |
| *Capra hircus* | 13 | Eutheria |  |
| *Monodelphis domestica* | 13 | Metatheria |  |
| *Sarcophilus harrisii* | 11 | Metatheria |  |

**Table S12. Statistical tests for characterization of the elusive genes that retained mammalian and avian orthologs**

| Madagascar ground gecko | | | | | | | |
| --- | --- | --- | --- | --- | --- | --- | --- |
| Genomic characteristics of elusive genes to be analyzed | Entire gene pairs of the elusive and non-elusive genes (156 pairs) | | | The pairs with  mammalian or avian orthologs (48 pairs) | | The pairs without mammalian and avian orthologs (108 pairs) | |
|  | Figs | *p*-value | Signed effect size^*^ | *p*-value | Signed effect size^*^ | *p*-value | Signed effect size^*^ |
| *K*_A_ | Fig. 4a | 4.47×10^-6^ | 0.476 | 0.0128 | 0.449 | 1.19×10^-3^ | 0.485 |
| *K*_S_ | Fig. 4b | 2.07×10^-4^ | 0.385 | 8.71×10^-3^ | 0.471 | 6.17×10^-3^ | 0.345 |
| *K*_A_ / *K*_S_ | Fig. 4c | 0.197 | 0.134 | 0.792 | 0.051 | 0.198 | 0.162 |
| unequal distribution in the genome | Fig. 5a | 8.27×10^-27^ | 1.345 | 2.41×10^-6^ | 0.312 | 2.99×10^-25^ | 2.459 |
| ortholog retention of the genes in the flanking regions | Fig. 5b | 4.18×10^-19^ | 0.254 | 4.03×10^-19^ | 0.185 | 1.27×10^-16^ | 0.286 |
| *K*_S_ of the genes in the flanking regions | Fig. 5c | 1.23×10^-17^ | 0.271 | 1.20×10^-4^ | 0.203 | 2.67×10^-15^ | 0.295 |
| gene density | Fig. 5d | 4.44×10^-16^ | 0.639 | 1.57×10^-5^ | 0.588 | 2.71×10^-11^ | 0.660 |
| repeat element density | Fig. 5e | 1.58×10^-4^ | 0.302 | 0.0397 | 0.296 | 1.21×10^-3^ | 0.311 |
| GC-content | Fig. 5f | 1.11×10^-10^ | 0.517 | 1.73×10^-3^ | 0.443 | 5.25×10^-8^ | 0.551 |
| Green Anole | | | | | | | |
| Genomic characteristics of elusive genes to be analyzed | Entire gene pairs of the elusive and common genes (180 pairs) | | | The pairs with  mammalian or avian orthologs (42 pairs) | | The pairs with no mammalian and avian orthologs (138 pairs) | |
|  | Figs | *p*-value | Signed effect size^*^ | *p*-value | Signed effect size^*^ | *p*-value | Signed effect size^*^ |
| *K*_A_ | Fig. 4d | 7.14×10^-8^ | 0.631 | 5.10×10^-4^ | 0.709 | 1.43×10^-5^ | 0.602 |
| *K*_S_ | Fig. 4e | 2.18×10^-8^ | 0.554 | 0.0113 | 0.542 | 5.07×10^-5^ | 0.562 |
| *K*_A_ / *K*_S_ | Fig. 4f | 0.0575 | 0.222 | 0.147 | 0.322 | 0.175 | 0.188 |
| unequal distribution in the genome | Fig. S7a | 1.53×10^-27^ | 1.379 | 0.0357 | 0.730 | 2.10×10^-34^ | 1.626 |
| ortholog retention of the genes in the flanking regions | Fig. S7b | 1.53×10^-27^ | 0.249 | 4.18×10^-19^ | 0.181 | 1.19×10^-15^ | 0.274 |
| *K*_S_ of the genes in the flanking regions | Fig. S7c | 1.55×10^-5^ | 0.274 | 5.81×10^-3^ | 0.437 | 4.97×10^-3^ | 0.204 |
| gene density | Fig. S7d | 1.37×10^-8^ | 0.411 | 7.57×10^-3^ | 0.245 | 1.37×10^-8^ | 0.466 |
| repeat element density | Fig. S7e | 7.94×10^-15^ | 0.550 | 5.19×10^-5^ | 0.532 | 3.82×10^-9^ | 0.556 |
| GC-content | Fig. S7f | 0.539 | 0.026 | 0.698 | -0.099 | 0.31 | 0.073 |

*Pearson *r* except for the analyses for 'unequal distribution in the genome' and 'ortholog retention of the genes in the flanking regions', which employed φ coefficient

**Table S13. Expression levels and evolutionary rates of green anole Rbfox genes**

| Green anole Protein ID | Locus name | Elusive | FPKM value | | | | | | | | | | | Madagascar ground gecko ortholog | *K*_A_ | *K*_S_ |
| --- | --- | --- | --- | --- | --- | --- | --- | --- | --- | --- | --- | --- | --- | --- | --- | --- |
|  |  |  | Skeletal muscle | Liver | Lung | Whole Embryo pooled | Adrenal gland | Heart pooled | Brain pooled | Ovary pooled | Dewlap pooled | Embryo 28S | Embryo 38S |  |  |  |
| ENSACAP00000020876 | Acc:G1KYG2  (zebrafish *rbfox1l*) | ✓ | 33.316 | 0.081 | 0.128 | 20.136 | 0.052 | 644.238 | 0.968 | 0.511 | 0.696 | 5.456 | 3.813 | g20460 | 0.0259 | 0.818 |
| XP_008108845.1 | *Rbfox2* |  | 1.467 | 7.836 | 17.084 | 31.113 | 4.860 | 4.706 | 31.573 | 6.694 | 6.875 | 52.295 | 39.027 | g4776 | 0.0066 | 0.247 |
| XP_008102381.1 | *Rbfox3* |  | <0.001 | 0 | <0.001 | 0 | <0.001 | 0 | 1.750 | 0 | 0 | 0 | <0.001 | g12410 | 0.0098 | 0.359 |
| ENSACAP00000004055 | *Rbfox1* |  | 0.0675 | 0.144 | 0.049 | 0.553 | 0.118 | 0.073 | 53.424 | 0.350 | 0.339 | 1.645 | 2.853 | ND |  |  |

**Table S14. Numbers of SINEs in the elusive and non-elusive genes and their flanking regions**

|  | Number of SINEs | | Number of SINEs/Kb | |
| --- | --- | --- | --- | --- |
|  | elusive gene + flanking regions | non-elusive gene + flanking regions | elusive gene + flanking regions | non-elusive gene + flanking regions |
| Madagascar ground gecko | 14007 | 11270 | 0.4946 | 0.383 |
| green anole | 7429 | 6400 | 0.2694 | 0.228 |
| Chinese softshell turtle | 3777 | 2142 | 0.161 | 0.0923 |
| American alligator | 2481 | 2124 | 0.121 | 0.0864 |
| western clawed frog | 31 | 32 | 0.0013 | 0.0015 |
| coelacanth | 12491 | 8847 | 0.3486 | 0.2616 |
| spotted gar | 10 | 1 | 0.0002 | <0.0001 |
| zebrafish | 746 | 616 | 0.0167 | 0.0169 |
| stickleback | 362 | 351 | 0.0114 | 0.0122 |

**Table S15. Numbers of the orthologs of the elusive genes in the non-mammalian/avian vertebrates**

|  | Total | Species^*^ | | | | | | | | | | | | | |
| --- | --- | --- | --- | --- | --- | --- | --- | --- | --- | --- | --- | --- | --- | --- | --- |
|  |  | ALIMI | ALISI | PELSI | CHEMY | PARPI | GEKJA | ANOCA | PYTBI | THASI | XENTR | LATCH | LEPOC | DANRE | GASAC |
| Elusive genes with non-elusive paralogs | 263 | 139 | 132 | 132 | 136 | 154 | 169 | 176 | 147 | 112 | 124 | 202 | 204 | 203 | 161 |
| Non-elusive paralogs | 263 | 256 | 258 | 255 | 254 | 245 | 252 | 250 | 249 | 223 | 241 | 246 | 252 | 244 | 229 |
| Other elusive genes | 206 | 85 | 90 | 115 | 82 | 107 | 84 | 122 | 77 | 68 | 83 | 132 | 127 | 101 | 75 |

^*^The abbreviations of the species names are shown in Table S7.

**Table S16. Conditions of library preparation for genome sequencing**

| Library ID | DNA start amount (μg) | Insert size distribution of mate-pair libraries (kb) | Shearing condition using Covaris | | | | | # PCR cycles | AMPure volume | Insert size (bp) |
| --- | --- | --- | --- | --- | --- | --- | --- | --- | --- | --- |
|  |  |  | Peak Power | Duty Factor (%) | Cycle/ Burst | Run Time (s) | Times repeated |  |  |  |
| Paired-end libraries | |  |  |  |  |  |  |  |  |  |
| P079_01_1 | 1 | NA | 140 | 10 | 200 | 55 | 2 | 0 | ×0.4/×1.0^*^ | 380 |
| P079_01_2 | 2 | NA | 105 | 5 | 200 | 50/80 | 2 | 0 | ×0.4/×0.9^*^ | 480 |
| P079_01_3 | 4 | NA | 105 | 5 | 200 | 50 | 3 | 0 | ×0.2/×0.6^*^ | 610 |
| Mate Pair libraries | |  |  |  |  |  |  |  |  |  |
| P091_01_1 | 4 | 1-6 | 240 | 20 | 200 | 40 | 2 | 10 | ×0.67 | 663 |
| P101_01_1 | 4 | 3-8 | 240 | 20 | 200 | 40 | 3 | 8 | ×0.67 | 487 |
| P101_01_2 | 4 | 6-10 | 240 | 20 | 200 | 40 | 3 | 10 | ×0.67 | 485 |
| P101_01_3 | 4 | 1-6 | 240 | 20 | 200 | 40 | 3 | 8 | ×0.67 | 494 |
| P104_01_5 | 12 | 11-18 | 240 | 20 | 200 | 40 | 4 | 10 | ×0.67 | 411 |

*Double-sided size selection was performed to prepare the libraries.

**Table S17. Fossil calibrations for the divergence time inference**

| Split of two taxonomic groups or root of a taxonomic group | Upper limit (MYA) | Lower limit (MYA) | Source | Note |
| --- | --- | --- | --- | --- |
| Galloanserae-Neoaves | 66 | 86.8 | Fossil Calibration Database [[117](#_ENREF_117)] |  |
| aves-crocodiles | 247.1 | 260.2 | Fossil Calibration Database [[117](#_ENREF_117)] |  |
| Chaenophidia-Booidea | 64 | 100.5 | Fossil Calibration Database [[117](#_ENREF_117)] | The upper and lower limits were according to the maximum age of crown Alethinophidia and the minimum age of Boinae, respectively |
| Root of Toxicofera | 145 | - | Fossil Calibration Database [[117](#_ENREF_117)] | The age was according to the occurrence of Dorsetisaurus, the earliest anguimorph. |
| Root of squamata | 168.9 | 209.5 | Fossil Calibration Database [[117](#_ENREF_117)] |  |
| Archelosauria-Squamata | 255.9 | 295.9 | Fossil Calibration Database [[117](#_ENREF_117)] |  |
| human-dog | 95.3 | 113 | Fossil Calibration Database [[117](#_ENREF_117)] |  |
| eutherians-marsupials | 157.3 | 169.6 | Benton and Donoghue 2007 [[118](#_ENREF_118)] |  |
| mammals-sauropsids | 318 | 332.9 | Fossil Calibration Database [[117](#_ENREF_117)] |  |
| amniotes-amphibians | 337 | 351 | Fossil Calibration Database [[117](#_ENREF_117)] |  |

| **Table S18. List of primers for RNA probes** | | |  |  |
| --- | --- | --- | --- | --- |
| Species | Gene | Forward primer (5’→3’) | Reverse primer (5’→3’) | amplicon (bp) |
| *Paroedura picta* | *FoxG1* | CGCGGAGTCCAGACTGTAAG | ACATAACTGATTCGCCTCGC | 296 |
|  | *FoxG2* | TGAGAGACCTCGGGATG | CGCCATCATTGCCTCTGGC | 711 |
| *Danio rerio* | *foxg1a* | CCGTTCCTCTCGCTTCACC | GGATTTGATGTTGACCCTTGA | 521 |
|  | *foxg1b* | GGAAGCTGGTGATGAAACGC | GCTCAGGAATGAATTGGGCG | 491 |
|  | *foxg1c* | CCAAACTGGCCATGAAGAGA | GGAATGTCTGTACAAAGTCC | 523 |
|  | *foxg1d* | GGGAAACTGGCGATCAAACG | GCTCTCCTCATCACCAACTT | 496 |

**Table S19. Synonymous and nonsynonymous rates of Tas1R and opsin families**

| gecko peptide | green anole ortholog peptide | gene name (green anole) | elusive | *K*_A_ | *K*_S_ |
| --- | --- | --- | --- | --- | --- |
| Parpi0009621.p1 | ENSACAP00000020188 | Acc:H9GTM0 | ✓ | 0.2613 | 2.19 |
| Parpi0009450.p1 | ENSACAP00000011243 | *Tas1R1* |  | 0.2799 | 3.5758 |
| Parpi0019694.p1 | ENSACAP00000008510 | *Tas1R2* |  | 0.248 | 4.5555 |
| Parpi0019195.p1 | XP_008118621.1 | *Gprc6a* |  | 0.0502 | 0.6844 |
|  |  |  |  |  |  |
| Parpi0002005.p1 | XP_003228056.1 | TMT opsin 3 | ✓ | 0.2445 | 2.5161 |
| XP_015266708.1^*^ | ENSACAP00000002434 | TMT opsin | ✓ | 0.0875 | 0.5598 |
| Parpi0003983.p1 | ENSACAP00000005696 | VA-opsin |  | 0.0705 | 0.733 |
| Parpi0004802.p1 | ENSACAP00000008376 | Pinopsin |  | 0.1254 | 1.6087 |
| Parpi0000830.p1 | ENSACAP00000009943 | *RRH* |  | 0.0599 | 0.7123 |
| Parpi0014502.p1 | ENSACAP00000013257 | *OPN5* |  | 0.0518 | 1.0195 |
| Parpi0012107.p1 | XP_003215983.1 | *OPN6b* |  | 0.0688 | 0.8448 |
| Parpi0010748.p1 | ENSACAP00000013346 | *OPN4x* |  | 0.0821 | 0.7311 |
| Parpi0015150.p1 | ENSACAP00000003346 | *OPN3* |  | 0.0548 | 0.6422 |
| Parpi0000556.p1 | XP_008105272.1 | TMT-like |  | 0.0821 | 1.0122 |
| Parpi0003314.p1 | XP_003223311.2 | *RGR* |  | 0.0797 | 0.8873 |

^*^The sequence retrieved from the *Gekko japonicus* genome

**References**

91. Yarmolinsky DA, Zuker CS, Ryba NJ. Common sense about taste: from mammals to insects. Cell. 2009;139(2):234-44.

92. Oike H, Nagai T, Furuyama A, Okada S, Aihara Y, Ishimaru Y, et al. Characterization of ligands for fish taste receptors. J Neurosci. 2007;27(21):5584-92.

93. Woolfe A, Goode DK, Cooke J, Callaway H, Smith S, Snell P, et al. CONDOR: a database resource of developmentally associated conserved non-coding elements. BMC Dev Biol. 2007;7:100.

94. Venkatesh B, Lee AP, Ravi V, Maurya AK, Lian MM, Swann JB, et al. Elephant shark genome provides unique insights into gnathostome evolution. Nature. 2014;505(7482):174-9.

95. Uchiyama I, Mihara M, Nishide H, Chiba H. MBGD update 2015: microbial genome database for flexible ortholog analysis utilizing a diverse set of genomic data. Nucleic Acids Res. 2015;43(Database issue):D270-6.

96. Altschul SF, Madden TL, Schaffer AA, Zhang J, Zhang Z, Miller W, et al. Gapped BLAST and PSI-BLAST: a new generation of protein database search programs. Nucleic Acids Res. 1997;25(17):3389-402.

97. Faircloth BC, McCormack JE, Crawford NG, Harvey MG, Brumfield RT, Glenn TC. Ultraconserved elements anchor thousands of genetic markers spanning multiple evolutionary timescales. Syst Biol. 2012;61(5):717-26.

98. Dimitrieva S, Bucher P. UCNEbase--a database of ultraconserved non-coding elements and genomic regulatory blocks. Nucleic Acids Res. 2013;41(Database issue):D101-9.

99. Simao FA, Waterhouse RM, Ioannidis P, Kriventseva EV, Zdobnov EM. BUSCO: assessing genome assembly and annotation completeness with single-copy orthologs. Bioinformatics. 2015;31(19):3210-2.

100. Slater GS, Birney E. Automated generation of heuristics for biological sequence comparison. BMC Bioinformatics. 2005;6:31.

101. Kadota M, Hara Y, Tanaka K, Takagi W, Tanegashima C, Nishimura O, et al. CTCF binding landscape in jawless fish with reference to Hox cluster evolution. Sci Rep. 2017;7(1):4957.

102. Haas BJ, Delcher AL, Mount SM, Wortman JR, Smith RK, Jr., Hannick LI, et al. Improving the *Arabidopsis* genome annotation using maximal transcript alignment assemblies. Nucleic Acids Res. 2003;31(19):5654-66.

103. Keller O, Kollmar M, Stanke M, Waack S. A novel hybrid gene prediction method employing protein multiple sequence alignments. Bioinformatics. 2011;27(6):757-63.

104. Katoh K, Standley DM. MAFFT multiple sequence alignment software version 7: improvements in performance and usability. Mol Biol Evol. 2013;30(4):772-80.

105. O'Leary NA, Wright MW, Brister JR, Ciufo S, Haddad D, McVeigh R, et al. Reference sequence (RefSeq) database at NCBI: current status, taxonomic expansion, and functional annotation. Nucleic Acids Res. 2016;44(D1):D733-45.

106. Li W, Godzik A. Cd-hit: a fast program for clustering and comparing large sets of protein or nucleotide sequences. Bioinformatics. 2006;22(13):1658-9.

107. Emms DM, Kelly S. OrthoFinder: solving fundamental biases in whole genome comparisons dramatically improves orthogroup inference accuracy. Genome Biol. 2015;16:157.

108. Huerta-Cepas J, Capella-Gutierrez S, Pryszcz LP, Denisov I, Kormes D, Marcet-Houben M, et al. PhylomeDB v3.0: an expanding repository of genome-wide collections of trees, alignments and phylogeny-based orthology and paralogy predictions. Nucleic Acids Res. 2011;39(Database issue):D556-60.

109. Sievers F, Wilm A, Dineen D, Gibson TJ, Karplus K, Li W, et al. Fast, scalable generation of high-quality protein multiple sequence alignments using Clustal Omega. Mol Syst Biol. 2011;7:539.

110. Do CB, Mahabhashyam MS, Brudno M, Batzoglou S. ProbCons: Probabilistic consistency-based multiple sequence alignment. Genome Res. 2005;15(2):330-40.

111. Wallace IM, O'Sullivan O, Higgins DG, Notredame C. M-Coffee: combining multiple sequence alignment methods with T-Coffee. Nucleic Acids Res. 2006;34(6):1692-9.

112. Capella-Gutierrez S, Silla-Martinez JM, Gabaldon T. trimAl: a tool for automated alignment trimming in large-scale phylogenetic analyses. Bioinformatics. 2009;25(15):1972-3.

113. Minh BQ, Nguyen MA, von Haeseler A. Ultrafast approximation for phylogenetic bootstrap. Mol Biol Evol. 2013;30(5):1188-95.

114. Workman RE, Myrka AM, Tseng E, Wong GW, Welch KC, Timp W. Single molecule, full-length transcript sequencing provides insight into the extreme metabolism of ruby-throated hummingbird Archilochus colubris. bioRxiv. 2017.

115. Michelle N. Vierra, Sarah B. Kingan, Elizabeth Tseng, Tyson Clark, Ting Hon, William J. Rowell, et al., editors. From RNA to Full-Length Transcripts: The PacBio Iso-Seq Method for Transcriptome Analysis and Genome Annotation. Genome10K and Genome Science Conference; 2017; Earlham Institute, Norwich, England.

116. Steinegger M, Soding J. MMseqs2 enables sensitive protein sequence searching for the analysis of massive data sets. Nat Biotechnol. 2017;35(11):1026-8.

117. Ksepka DT, Parham JF, Allman JF, Benton MJ, Carrano MT, Cranston KA, et al. The Fossil Calibration Database-A New Resource for Divergence Dating. Syst Biol. 2015;64(5):853-9.

118. Benton MJ, Donoghue PC. Paleontological evidence to date the tree of life. Mol Biol Evol. 2007;24(1):26-53.
