# Supplementary material for: Madagascar ground gecko genome analysis characterizes asymmetric fates of duplicated genes
Source: BMC Biol. 2018 Apr 16;16:40. doi: 10.1186/s12915-018-0509-4 (PMC5901865; doi:10.1186/s12915-018-0509-4)
Supplement: Supplementary file 2 — Figure S1. Repeat landscape. (PDF 182 kb) [file 12915_2018_509_MOESM2_ESM.pdf]

Additional file 2

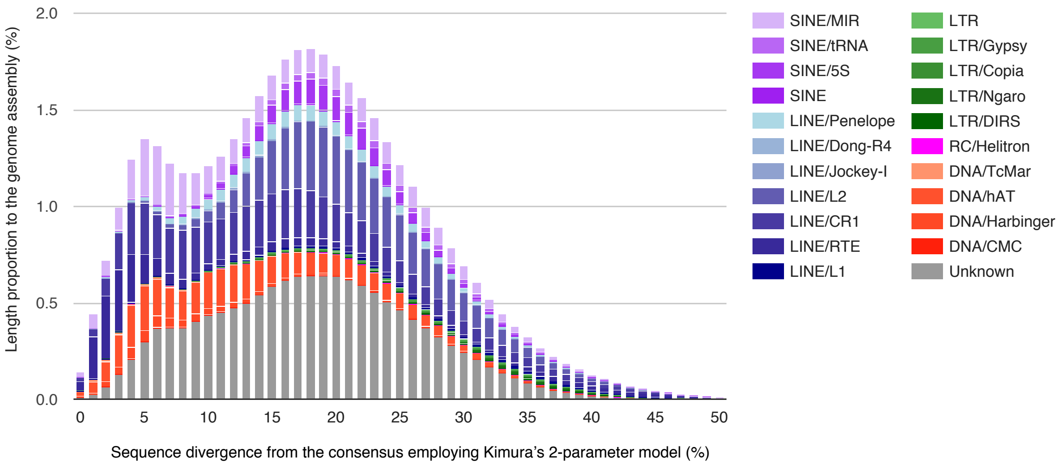

Figure S1. Repeat landscape

Relative abundances of repeat classes in the *P. picta* genome assembly are shown along sequence divergences against consensuses, which can be correlated to the age from the generation of the repeats, based on the Kimura's two-parameter method. The figure was produced with the script createRepeatLandscape.pl supplied with RepeatMasker. Detailed statistics of the repeat contents are included in Additional file 1: Table S4.
