# Supplementary material for: Madagascar ground gecko genome analysis characterizes asymmetric fates of duplicated genes
Source: BMC Biol. 2018 Apr 16;16:40. doi: 10.1186/s12915-018-0509-4 (PMC5901865; doi:10.1186/s12915-018-0509-4)
Supplement: Supplementary file 3 — Figure S2. Difference in GC content across the amniote genomes. (PDF 654 kb) [file 12915_2018_509_MOESM3_ESM.pdf]

Additional file 3

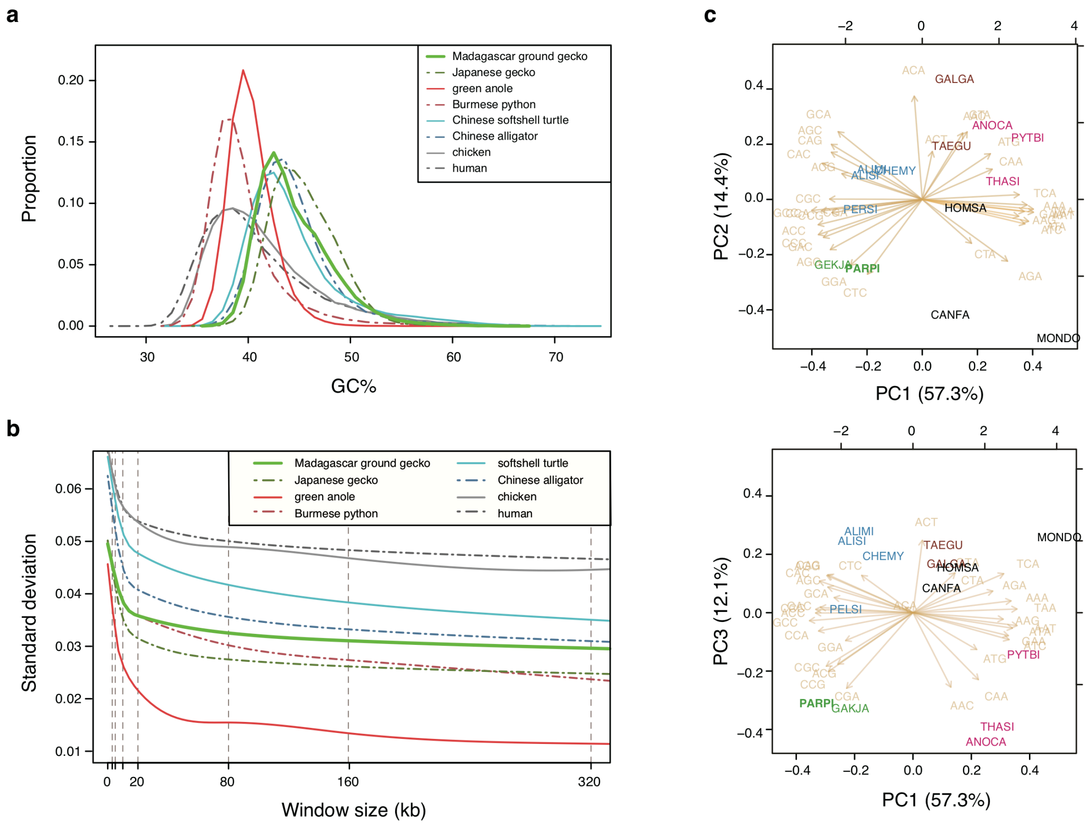

Figure S2. Difference in GC-content across the amniote genomes

(**a**) Frequency distribution of GC-content along the genome sequences with 20-kb non-overlapping windows. (**b**) Standard deviations of GC-content using 3, 5, 10, 20, 80, 160, and 320-kb non-overlapping windows. Curves connecting the plots were generated with the smoothe.spline library embedded by R. (**c**) Principal component analysis based on frequencies of 3-mer nucleotides. Plots consisting of PC1 and PC2 scores (upper panel) and PC1 and PC3 scores (lower panel) are shown. Percentages of explained variance of the principal components were shown for both X- and Y-axis. Abbreviations of species names are included in Additional file 1: Table S6. The second horizontal and vertical axes denote the scales for eigenvectors, which are shown in light brown. This analysis was performed using the prcomp and biplot packages implemented in R.
