# Supplementary material for: Madagascar ground gecko genome analysis characterizes asymmetric fates of duplicated genes
Source: BMC Biol. 2018 Apr 16;16:40. doi: 10.1186/s12915-018-0509-4 (PMC5901865; doi:10.1186/s12915-018-0509-4)
Supplement: Supplementary file 5 — Figure S3. Divergence times between major reptile lineages. (PDF 360 kb) [file 12915_2018_509_MOESM5_ESM.pdf]

Additional file 5

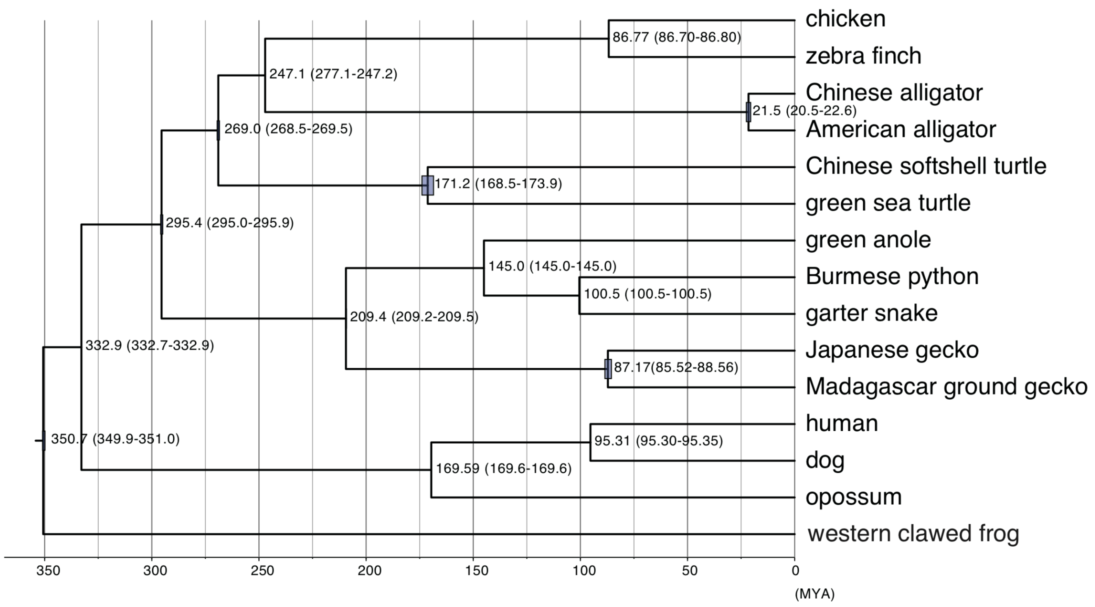

Figure S3. Divergence times between major reptile lineages

Inferred divergence times using 1,545 one-to-one orthologs. The median of the divergence time is scaled at a branching point, and a 95% credible interval (values are shown in parentheses) is represented as a width of a blue box. Fossil calibrations used for the divergence time inference are included in Additional file 1: Table S17.
