# Supplementary material for: Madagascar ground gecko genome analysis characterizes asymmetric fates of duplicated genes
Source: BMC Biol. 2018 Apr 16;16:40. doi: 10.1186/s12915-018-0509-4 (PMC5901865; doi:10.1186/s12915-018-0509-4)
Supplement: Supplementary file 6 — Figure S4. KS distribution of the P. picta genes. (PDF 210 kb) [file 12915_2018_509_MOESM6_ESM.pdf]

Additional file 6

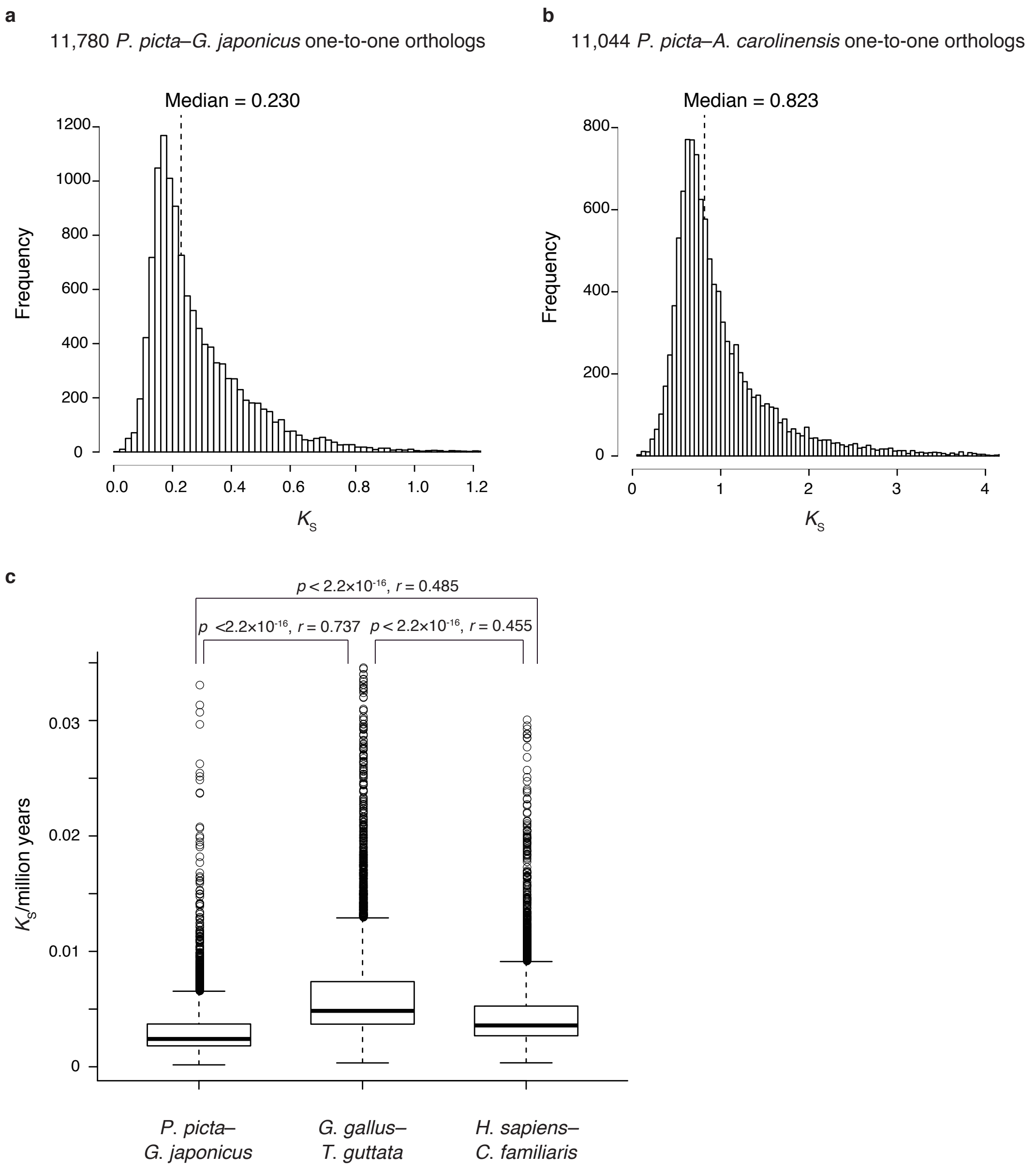

Figure S4. K_S distribution of the *P. picta* genes

(a) Frequency distribution of K_S for one-to-one ortholog pairs of Madagascar ground gecko and Japanese gecko. The histogram contained 11,780 ortholog pairs out of 12,889 (Additional file 1: Table S9) with ≥300 aligned nucleotide sites and $K_S < 3$. (b) Frequency distribution of K_S for one-to-one ortholog pairs of Madagascar ground gecko and green anole. The histogram contained 11,044 pairs out of 12,330 (Additional file 1: Table S9) with ≥300 aligned nucleotide sites and $K_S < 5$. (c) Distributions of K_S per million years between Madagascar ground gecko and Japanese gecko (median,

0.00243), between chicken and zebra finch (median, 0.00487), and between human and dog (median, 0.00361). For each pair, the *p*-value based on the Mann–Whitney *U* test and he effect size, denoted as Pearson *r,* were shown. The distribution is composed by 6,231 one-to-one ortholog sextets that were retained by these species with ≥300 aligned nucleotide sites with $K_S < 3$ for any of the gecko, avian, and mammalian pairs. The median of divergence times included in Table 2 were used for the normalization.
