# Supplementary material for: Madagascar ground gecko genome analysis characterizes asymmetric fates of duplicated genes
Source: BMC Biol. 2018 Apr 16;16:40. doi: 10.1186/s12915-018-0509-4 (PMC5901865; doi:10.1186/s12915-018-0509-4)
Supplement: Supplementary file 7 — Figure S5. Opsin gene tree. (PDF 3021 kb) [file 12915_2018_509_MOESM7_ESM.pdf]

Additional file 7

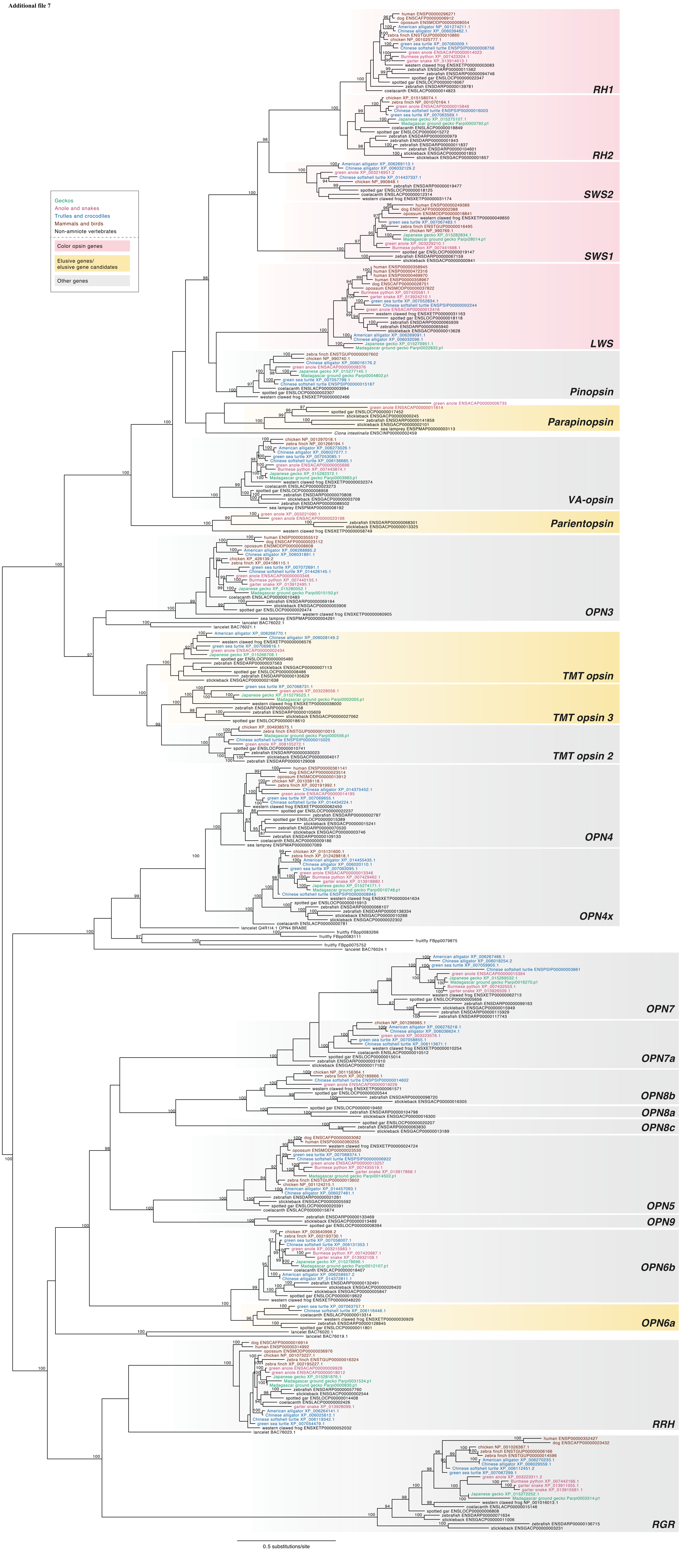

Figure S5. Opsin gene tree

The maximu likelihood tree of visual and non-visual opsin genes using 242 alignment sites of the amino acid sequences, assuming the JTT+F+R8 model. The tree contained the peptides belonging to the ortholog groups of AmnPhylome_0000366, AmnPhylome_0002506, AmnPhylome_0002912, AmnPhylome_0003128, AmnPhylome_0003234, and AmnPhylome_0005032. The *P. picta* predicted peptide Parpi0000830.p1, which was partly homologous to opsin but belonged to a non-opsin gene family AmnPhylome_0002007 was added in the alignment. Additionally, opsin peptides of tunicate, lancelet, and fruitfly were included as in a previous study by Kuraku and Kuratani (2011) [12]. Values at the nodes denote ultrafast bootstrap approximation values with high reliability (≥90 and SH-aLRT values≥0.8). Branches of the geckos, anole/snakes, turtles/crocodiles, and mammalian/avian lineages are in green, red, blue, and brown, respectively. Subtrees with pink backgrounds indicate visual opsins, and those with yellow backgrounds indicatethe genes that retain reptile orthologs but not mammals and avians ones. The yellow-background subtree with the orthologs of non-amniote vertebrates was defined as the elusive genes. Among these genes, TMT opsin and TMT opsin 3, which retained non-amniote vertebrates, were defined as the loss-prone genes.
