# Supplementary material for: Madagascar ground gecko genome analysis characterizes asymmetric fates of duplicated genes
Source: BMC Biol. 2018 Apr 16;16:40. doi: 10.1186/s12915-018-0509-4 (PMC5901865; doi:10.1186/s12915-018-0509-4)
Supplement: Supplementary file 8 — Figure S6. Beta-keratin gene tree. (PDF 666 kb) [file 12915_2018_509_MOESM8_ESM.pdf]

Additional file 8

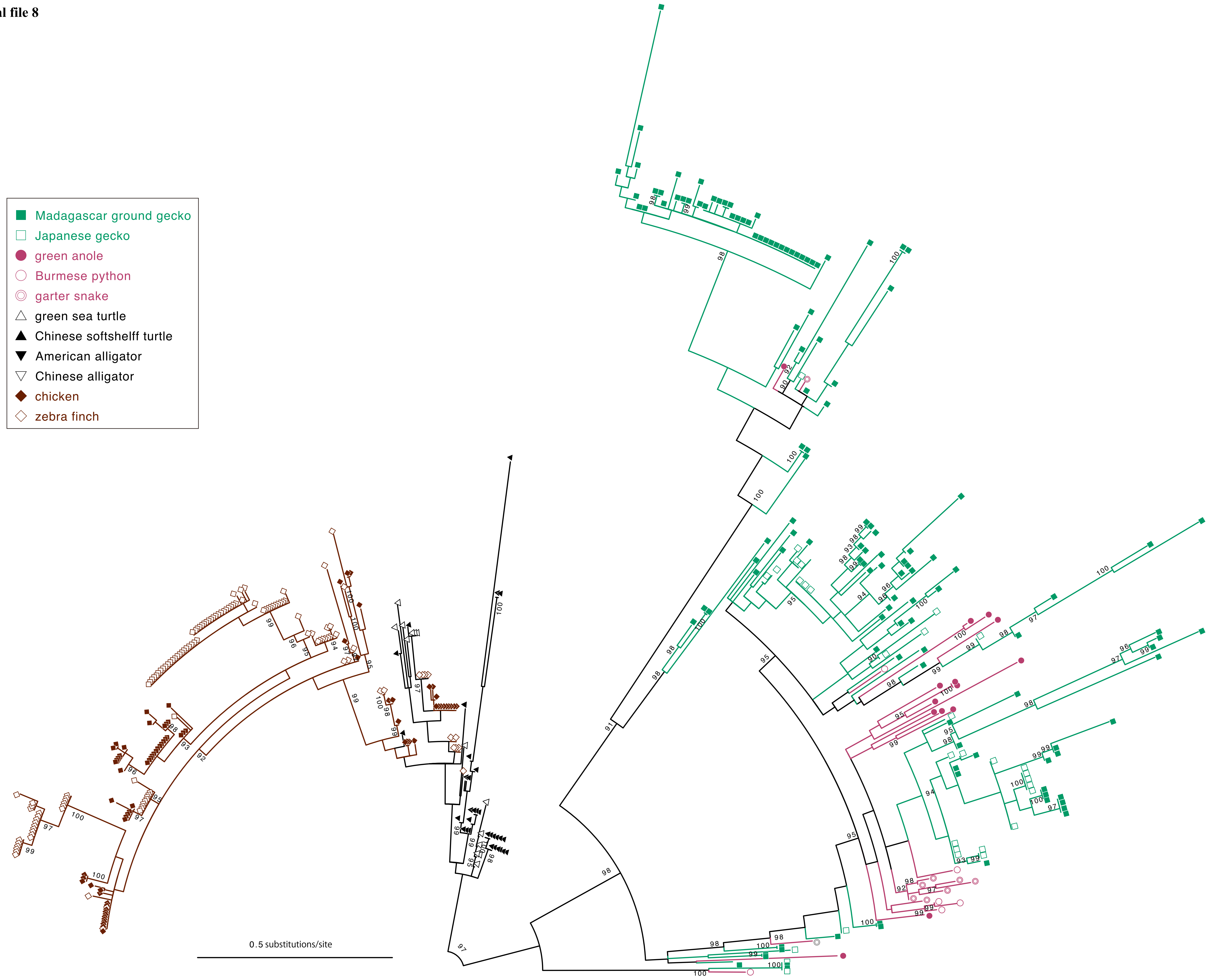

Figure S6. Beta-keratin gene tree

Phylogenetic tree of beta-keratin genes composed by 40 aligned sites of the amino acid sequences of the ortholog group AmnPhylome_0000003. The tree was inferred with IQ-TREE assuming the LG4X model. Gene names were replaced with the symbols corresponding to the species for visibility. Values at the nodes are ultrafast bootstrap approximation values with high reliability (≥90). Branches of the geckos, and anole/snakes, and avian lineages are in green, red, and brown, respectively. Some of the truncated sequences of the Madagascar ground gecko beta-keratin genes were removed from the multiple alignment for the tree inference. The Japanese gecko homologs were retrieved from the RefSeq database, though the 71 beta-keratin genes of this gecko identified by its genome sequencing project were unavailable.
