# Supplementary material for: Madagascar ground gecko genome analysis characterizes asymmetric fates of duplicated genes
Source: BMC Biol. 2018 Apr 16;16:40. doi: 10.1186/s12915-018-0509-4 (PMC5901865; doi:10.1186/s12915-018-0509-4)
Supplement: Supplementary file 10 — Figure S7. Genomic characters of the elusive and their non-elusive paralogs in the anole genome. (PDF 820 kb) [file 12915_2018_509_MOESM10_ESM.pdf]

Additional file 10

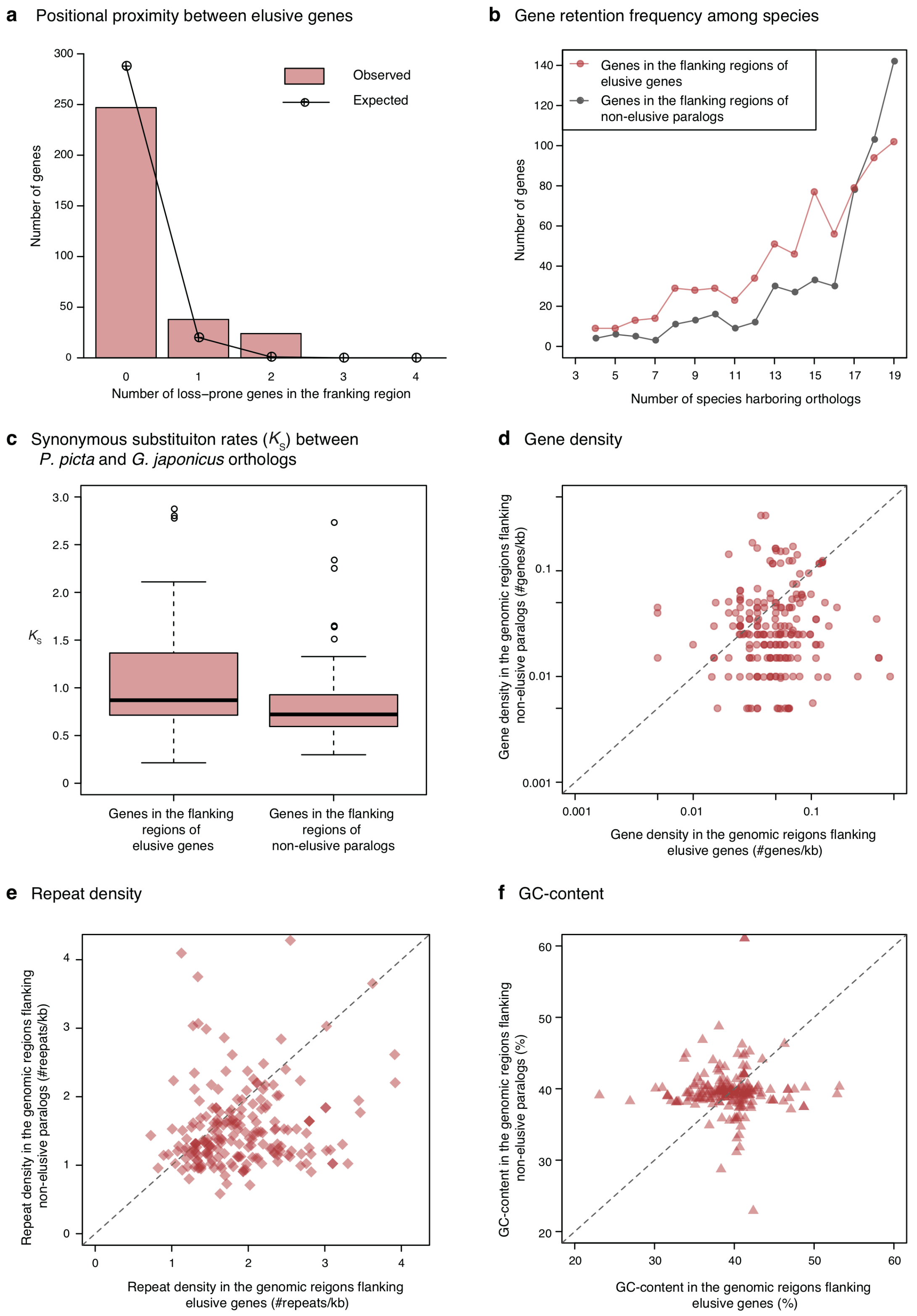

Figure S7. Genomic characters of the elusive and their non-elusive paralogs in the anole genome

(**a**) Fractions of the 298 elusive genes in the anole genome flanking other elusive genes. The observed and expected values are significantly different ($p = 1.53 \times 10^{-27}$, the exact test of goodness of fit). (**b**) Frequency distributions of the genes flanked by the 176 elusive genes and their non-elusive paralogs in accordance with the numbers of species retaining their orthologs. The two distributions exhibit a significant difference ($p = 3.37 \times 10^{-16}$, Mann–Whitney U test). (**c**) Comparison of synonymous substitution rates (K_S) comparison between the genes flanking the elusive and non-elusive genes. These K_S were significantly different ($p = 1.55 \times 10^{-5}$, Mann–Whitney U test). Each K_S was computed with an ortholog pair of Madagascar ground gecko and green anole. (**d**) Gene densities in the flanking regions of the elusive genes and their non-elusive paralogs, which are significantly different between these two regions ($p = 1.37 \times 10^{-8}$, Wilcoxon signed-rank test). (**e**) Repeat densities of the genomic regions containing the elusive genes or their non-elusive paralogs with their flanking regions, which are significantly different between the two regions ($p = 7.94 \times 10^{-15}$, Wilcoxon signed-rank test). (**f**) GC-contents consisting of the elusive genes or their non-elusive paralogs and their flanking regions, which are significantly different between the two regions ($p = 0.539$, Wilcoxon signed-rank test). See methodological details in Fig. 5.
