# Supplementary material for: Madagascar ground gecko genome analysis characterizes asymmetric fates of duplicated genes
Source: BMC Biol. 2018 Apr 16;16:40. doi: 10.1186/s12915-018-0509-4 (PMC5901865; doi:10.1186/s12915-018-0509-4)
Supplement: Supplementary file 11 — Figure S8. Gene densities in the 100 kb-flanking regions of the orthologs of the elusive reptile genes and their non-elusive genes. (PDF 1248 kb) [file 12915_2018_509_MOESM11_ESM.pdf]

Additional file 11

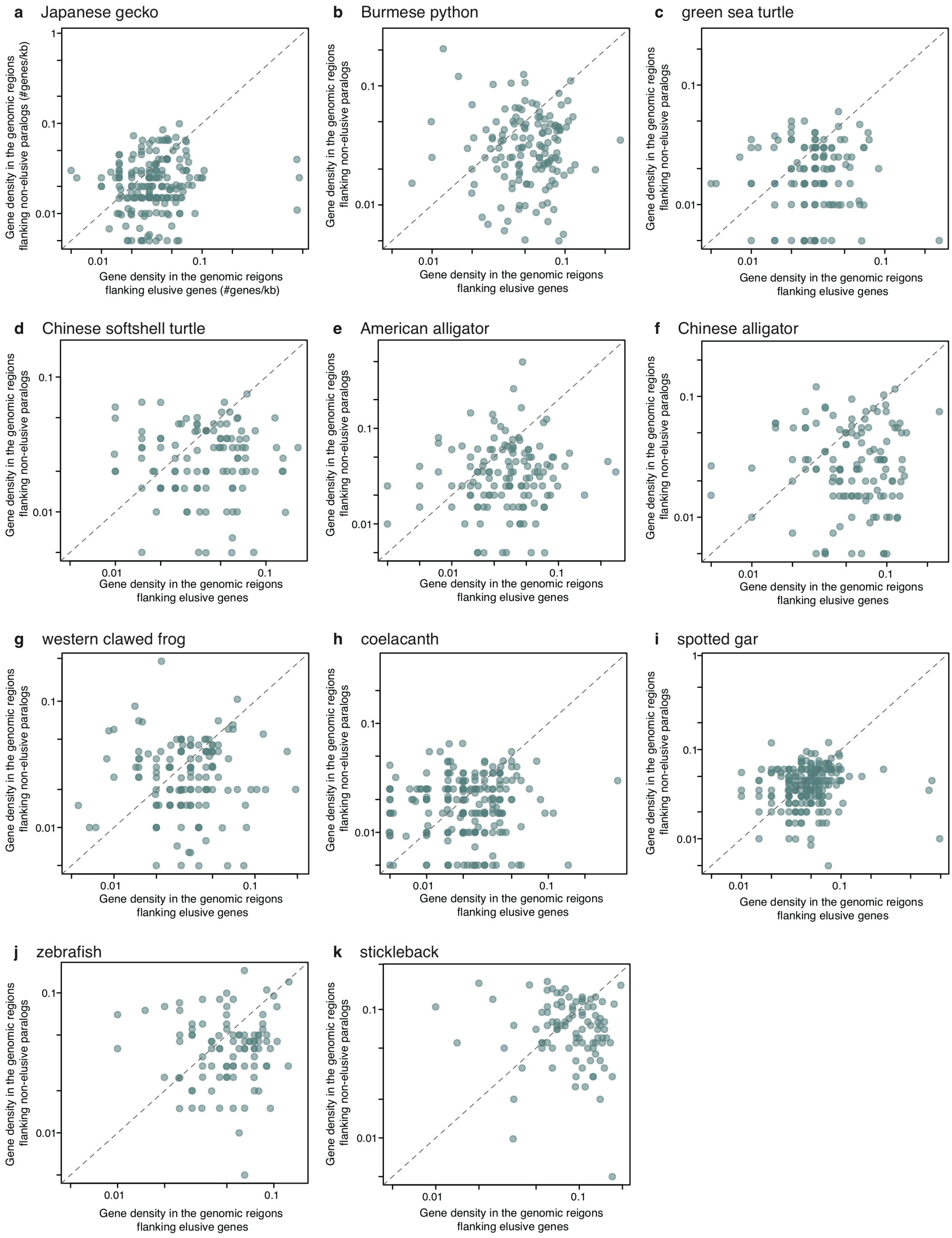

Figure S8. Gene densities in the 100 kb-flanking regions of the orthologs of the elusive reptile genes and their non-elusive genes

Gene densities of the flanking regions of the elusive genes and their non-elusive paralogs are shown for reptiles and outgroups. *P*-values and effect size below were computed based on the Wilcoxon signed-rank test and signed Pearson *r* between the two classes for each species, respectively: (**a**) $p = 3.16\times10^{-11}$, $r = 0.453$; (**b**) $p = 8.87\times10^{-11}$, $r = 0.537$; (**c**) $p = 2.27\times10^{-9}$, $r = 0.461$; (**d**) $p = 4.43\times10^{-9}$, $r = 0.465$; (**e**) $p = 9.98\times10^{-11}$, $r = 0.510$; (**f**) $p = 1.67\times10^{-12}$, $r = 0.563$; (**g**) $p = 1.94\times10^{-6}$, $r = 0.417$; (**h**) $p = 6.46\times10^{-6}$, $r = 0.284$; (**i**) $p = 0.000155$, $r = 0.226$; (**j**) $p = 0.000173$, $r = 0.196$; (**k**) $p = 0.0125$, $r = 0.213$.
