# Supplementary material for: Madagascar ground gecko genome analysis characterizes asymmetric fates of duplicated genes
Source: BMC Biol. 2018 Apr 16;16:40. doi: 10.1186/s12915-018-0509-4 (PMC5901865; doi:10.1186/s12915-018-0509-4)
Supplement: Supplementary file 12 — Figure S9. GC content in the genomic regions including the orthologs of the elusive reptile genes and their non-elusive genes. (PDF 1010 kb) [file 12915_2018_509_MOESM12_ESM.pdf]

Additional file 12

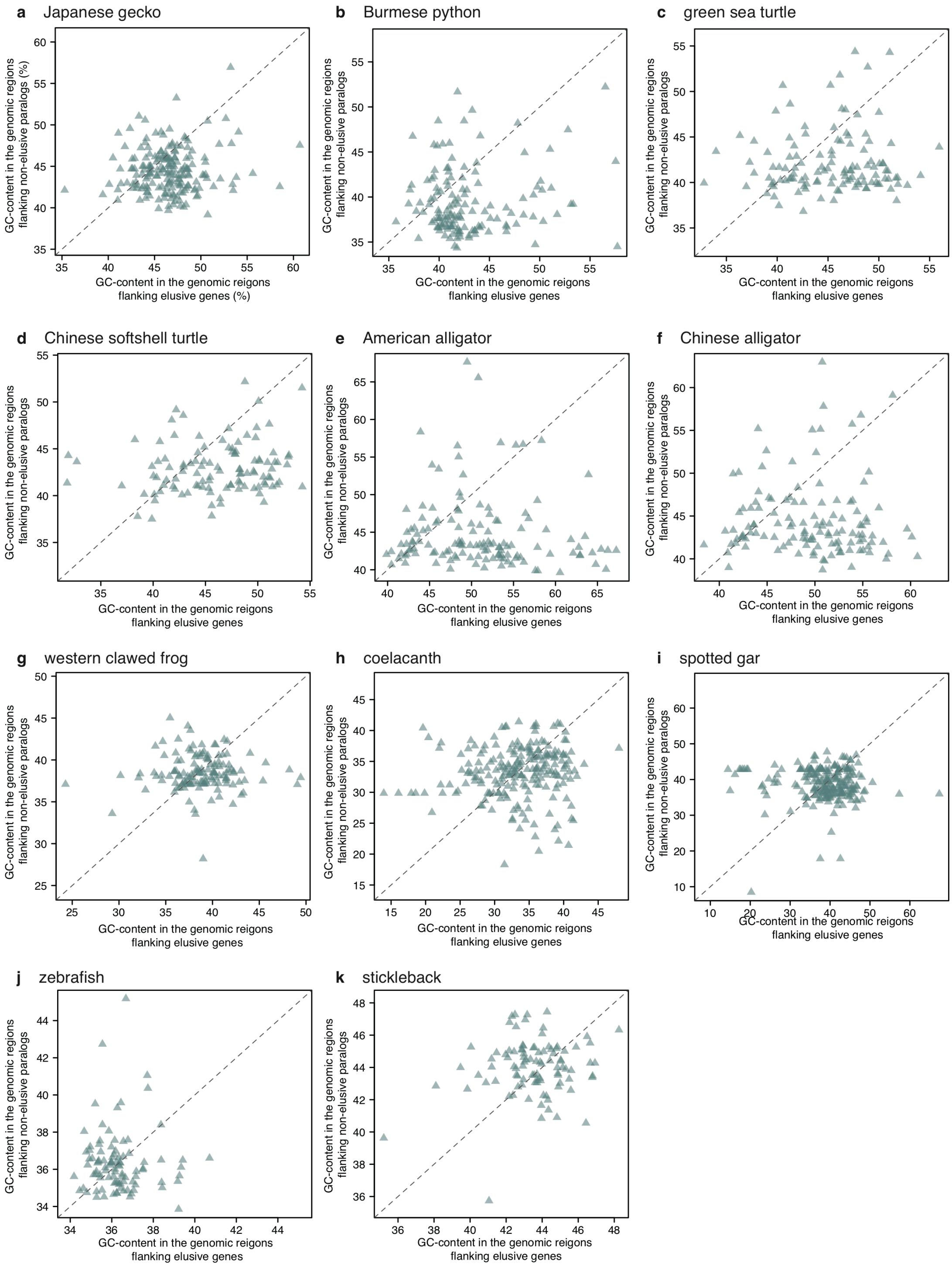

Figure S9. GC-content in the genomic regions including the orthologs of the elusive reptile genes and their non-elusive genes

GC-content of the genomic regions consisting of the elusive genes and their non-elusive paralogs with their flanking regions are shown for reptiles and outgroups. *P*-values and effect size below were computed based on the Wilcoxon signed-rank test and signed Pearson *r* between the two classes for each species, respectively: (**a**) $p = 4.85\times10^{-11}$, $r = 0.475$; (**b**) $p = 1.80\times10^{-13}$, $r = 0.591$; (**c**) $p = 2.51\times10^{-9}$, $r = 0.515$; (**d**) $p = 1.02\times10^{-10}$, $r = 0.552$; (**e**) $p = 1.65\times10^{-11}$, $r = 0.551$; (**f**) $p = 8.64\times10^{-13}$, $r = 0.509$; (**g**) $p = 0.776$, $r = -0.0250$; (**h**) $p = 0.188$, $r = 0.0922$; (**i**) $p = 0.0118$, $r = 0.170$; (**j**) $p = 0.0163$, $r = -0.143$; (**k**) $p = 0.0680$, $r = 0.159$.
