# Supplementary material for: Madagascar ground gecko genome analysis characterizes asymmetric fates of duplicated genes
Source: BMC Biol. 2018 Apr 16;16:40. doi: 10.1186/s12915-018-0509-4 (PMC5901865; doi:10.1186/s12915-018-0509-4)
Supplement: Supplementary file 13 — Figure S10. Repeat densities in the genomic regions including the orthologs of the elusive reptile genes and their non-elusive gene. (PDF 739 kb) [file 12915_2018_509_MOESM13_ESM.pdf]

Additional file 13

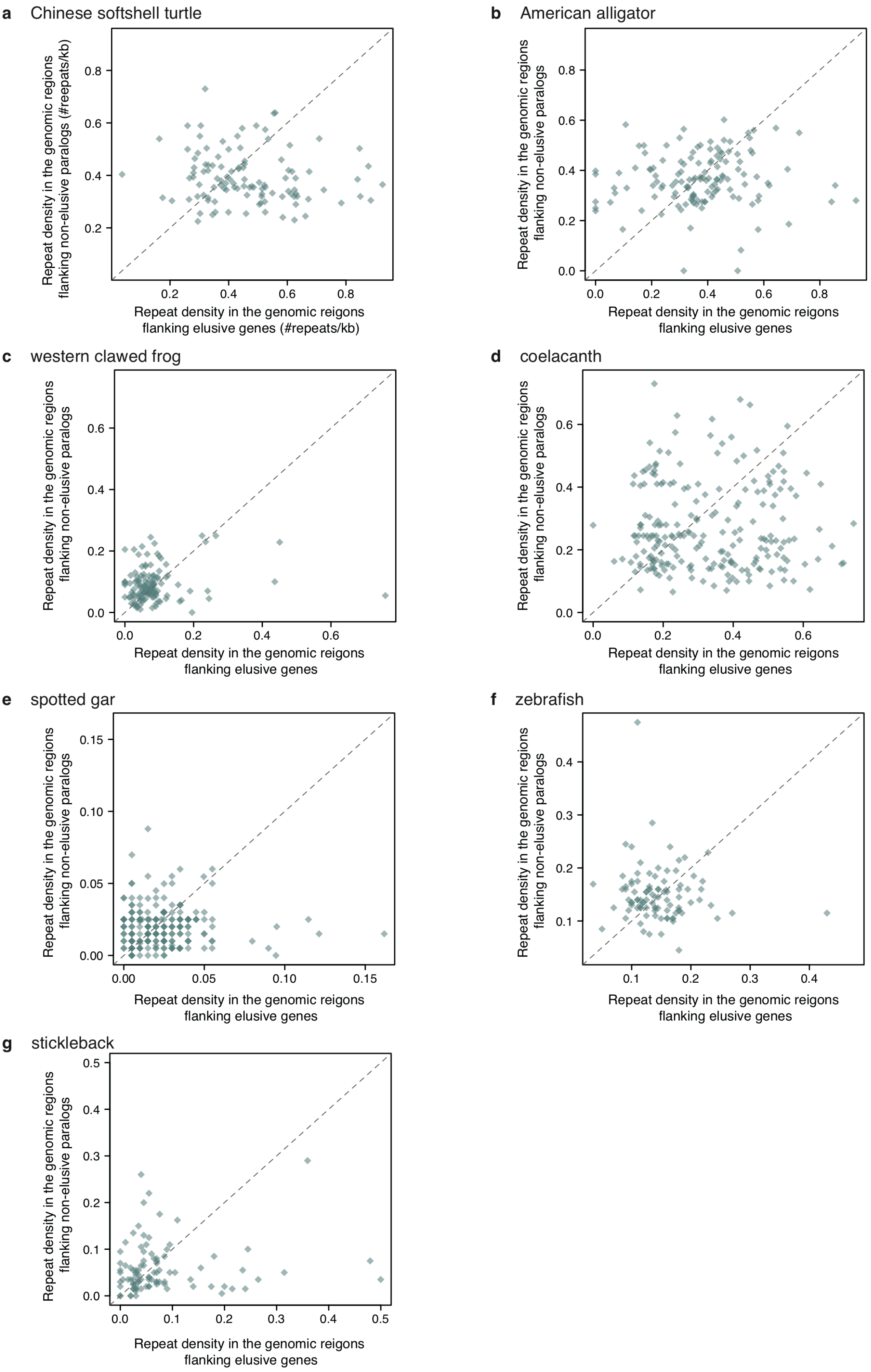

Figure S10. Repeat densities in the genomic regions including the orthologs of the elusive reptile genes and their non-elusive gene

Repeat densities of t the genomic regions consisting of the elusive genes and their non-elusive paralogs with their flanking regions are shown for reptiles and outgroups. We selected the species that repeat elements were already annotated and released as RepeatMasker Genomic Datasets (http://www.repeatmasker.org/genomicDatasets/RMGenomicDatasets.html). Additionally, repeat elements of the Chinese softshell turtle were annotated with RepeatMasker. *P*-values and effect size below were computed based on the Wilcoxon signed-rank test and signed Pearson *r* between the two classes for each species, respectively: (**a**) $p = 9.94\times10^{-5}$, $r = 0.332$; (**b**) $p = 0.757$, $r = 0.00106$; (**c**) $p = 0.101$, $r = -0.167$; (**d**) $p = 2.25\times10^{-7}$, $r = 0.351$; (**e**) $p = 0.00571$, $r = 0.0198$; (**f**) $p = 0.0413$, $r = -0.164$; (**g**) $p = 0.215$, $r = -0.155$.
