# Supplementary material for: Madagascar ground gecko genome analysis characterizes asymmetric fates of duplicated genes
Source: BMC Biol. 2018 Apr 16;16:40. doi: 10.1186/s12915-018-0509-4 (PMC5901865; doi:10.1186/s12915-018-0509-4)
Supplement: Supplementary file 14 — Figure S11. Frequency distribution of the elusive genes relating to the number of mammalian and avian orthologs. (PDF 299 kb) [file 12915_2018_509_MOESM14_ESM.pdf]

## Additional file 14

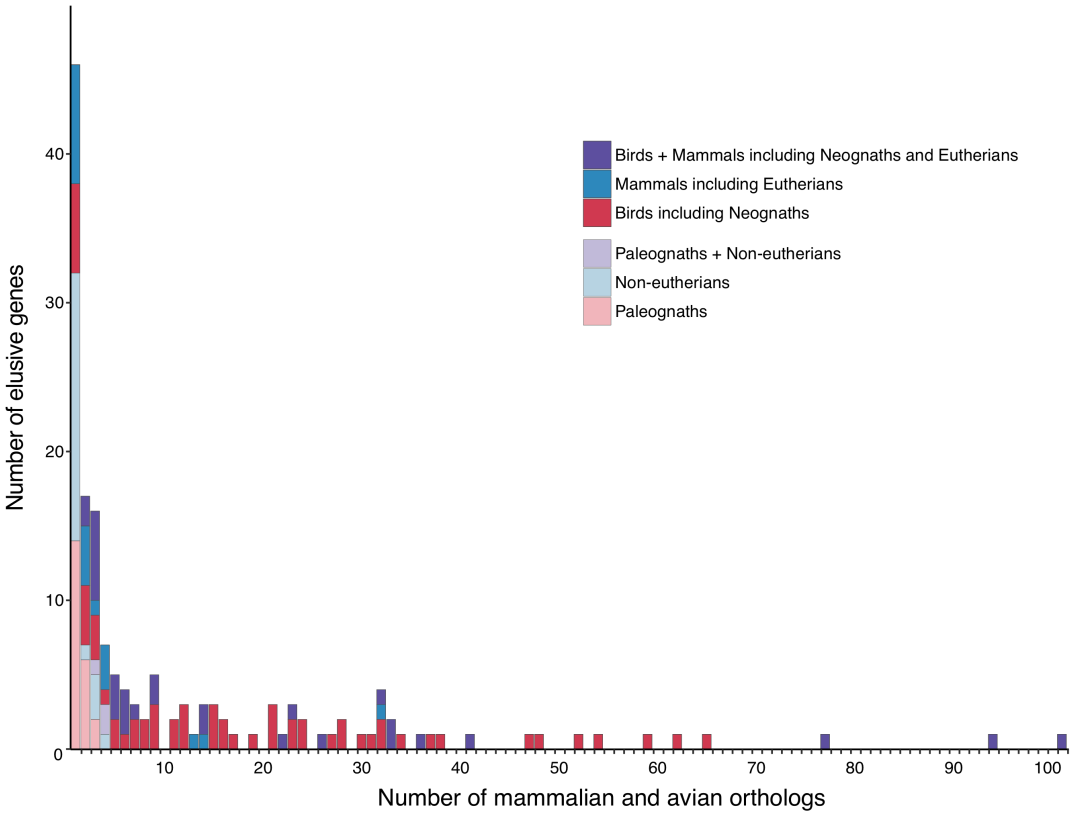


**Figure S11. Frequency distribution of the elusive genes relating to number of the mammalian and avian orthologs**

The graph consisted of 157 elusive genes whose putative orthologs of mammals and birds were identified. The elusive genes were classified into six groups in accordance with which taxonomy groups retained the orthologs.
