# Supplementary material for: Madagascar ground gecko genome analysis characterizes asymmetric fates of duplicated genes
Source: BMC Biol. 2018 Apr 16;16:40. doi: 10.1186/s12915-018-0509-4 (PMC5901865; doi:10.1186/s12915-018-0509-4)
Supplement: Supplementary file 15 — Figure S12. Comparison of expression levels between the elusive genes and non-elusive genes. (PDF 368 kb) [file 12915_2018_509_MOESM15_ESM.pdf]

Additional file 15

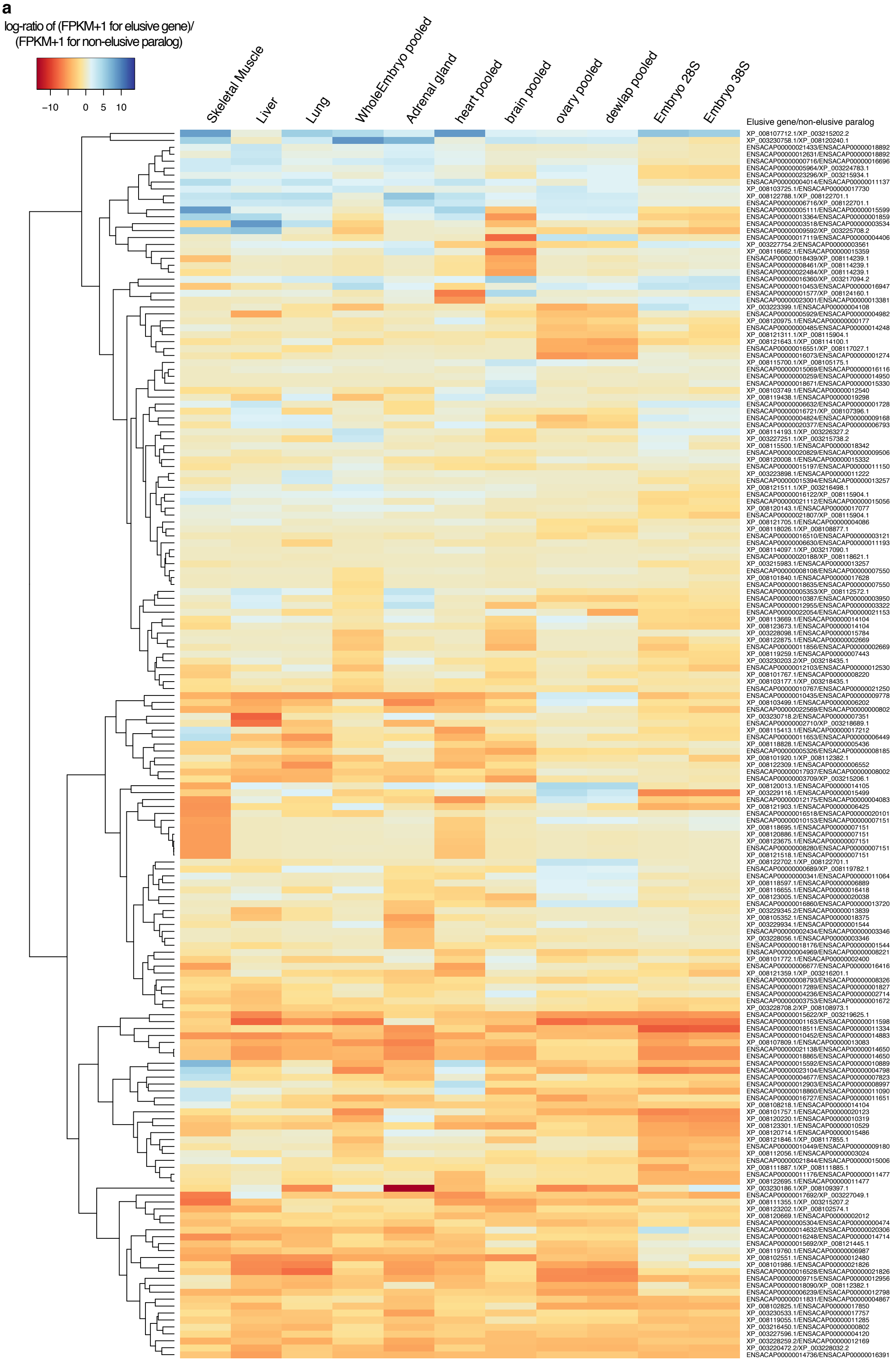

b

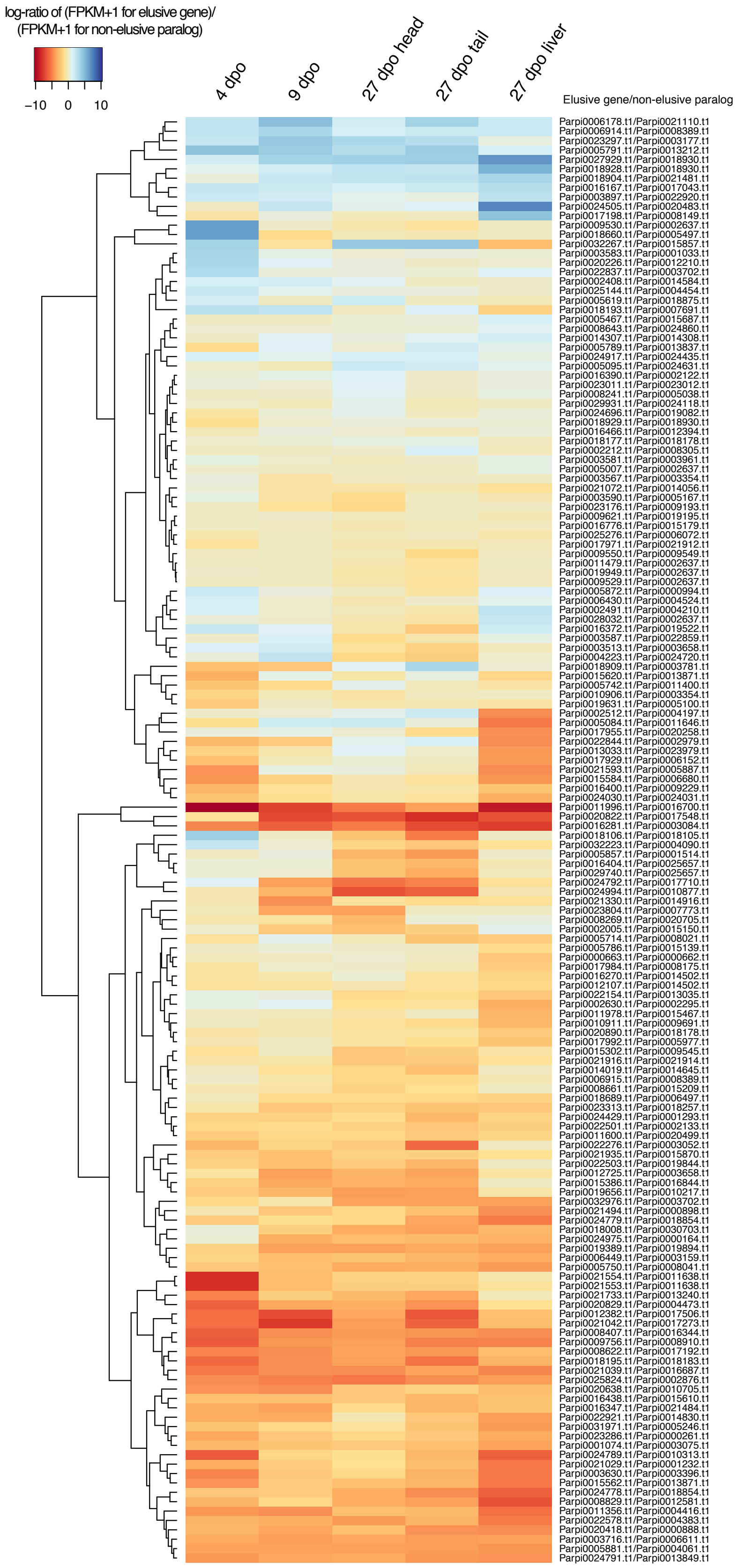
log-ratio of (FPKM+1 for elusive gene)/
(FPKM+1 for non-elusive paralog)
−10
0
10
4 dpo
9 dpo
27 dpo head
27 dpo tail
27 dpo liver
Elusive gene/non-elusive paralog
Parpi0006178.t1/Parpi0021110.t1
Parpi0006914.t1/Parpi0008389.t1
Parpi0023297.t1/Parpi0003177.t1
Parpi0005791.t1/Parpi0013212.t1
Parpi0027929.t1/Parpi0018930.t1
Parpi0018928.t1/Parpi0018930.t1
Parpi0018904.t1/Parpi0021481.t1
Parpi0016167.t1/Parpi0017043.t1
Parpi0003897.t1/Parpi0022920.t1
Parpi0024505.t1/Parpi0020483.t1
Parpi0017198.t1/Parpi0008149.t1
Parpi0009530.t1/Parpi0002637.t1
Parpi0018660.t1/Parpi0005497.t1
Parpi0032267.t1/Parpi0015857.t1
Parpi0003583.t1/Parpi0001033.t1
Parpi0020226.t1/Parpi0012210.t1
Parpi0022837.t1/Parpi0003702.t1
Parpi0002408.t1/Parpi0014584.t1
Parpi0025144.t1/Parpi0004454.t1
Parpi0005619.t1/Parpi0018875.t1
Parpi0018193.t1/Parpi0007691.t1
Parpi0005467.t1/Parpi0015687.t1
Parpi0008643.t1/Parpi0024860.t1
Parpi0014307.t1/Parpi0014308.t1
Parpi0005789.t1/Parpi0013837.t1
Parpi0024917.t1/Parpi0024435.t1
Parpi0005095.t1/Parpi0024631.t1
Parpi0016390.t1/Parpi0002122.t1
Parpi0023011.t1/Parpi0023012.t1
Parpi0008241.t1/Parpi0005038.t1
Parpi0029931.t1/Parpi0024118.t1
Parpi0024696.t1/Parpi0019082.t1
Parpi0018929.t1/Parpi0018930.t1
Parpi0016466.t1/Parpi0012394.t1
Parpi0018177.t1/Parpi0018178.t1
Parpi0002212.t1/Parpi0008305.t1
Parpi0003581.t1/Parpi0003961.t1
Parpi0005007.t1/Parpi0002637.t1
Parpi0003567.t1/Parpi0003354.t1
Parpi0021072.t1/Parpi0014056.t1
Parpi0003590.t1/Parpi0005167.t1
Parpi0023176.t1/Parpi0009193.t1
Parpi0009621.t1/Parpi0019195.t1
Parpi0016776.t1/Parpi0015179.t1
Parpi0025276.t1/Parpi0006072.t1
Parpi0017971.t1/Parpi0021912.t1
Parpi0009550.t1/Parpi0009549.t1
Parpi0011479.t1/Parpi0002637.t1
Parpi0019949.t1/Parpi0002637.t1
Parpi0009529.t1/Parpi0002637.t1
Parpi0005872.t1/Parpi0000994.t1
Parpi0006430.t1/Parpi0004524.t1
Parpi0002491.t1/Parpi0004210.t1
Parpi0028032.t1/Parpi0002637.t1
Parpi0016372.t1/Parpi0019522.t1
Parpi0003587.t1/Parpi0022859.t1
Parpi0003513.t1/Parpi0003658.t1
Parpi0004223.t1/Parpi0024720.t1
Parpi0018909.t1/Parpi0003781.t1
Parpi0015620.t1/Parpi0013871.t1
Parpi0005742.t1/Parpi0011400.t1
Parpi0010906.t1/Parpi0003354.t1
Parpi0019631.t1/Parpi0005100.t1
Parpi0002512.t1/Parpi0004197.t1
Parpi0005084.t1/Parpi0011646.t1
Parpi0017955.t1/Parpi0020258.t1
Parpi0022844.t1/Parpi0002979.t1
Parpi0013033.t1/Parpi0023979.t1
Parpi0017929.t1/Parpi0006152.t1
Parpi0021593.t1/Parpi0005887.t1
Parpi0015584.t1/Parpi0006680.t1
Parpi0016400.t1/Parpi0009229.t1
Parpi0024030.t1/Parpi0024031.t1
Parpi0011996.t1/Parpi0016700.t1
Parpi0020822.t1/Parpi0017548.t1
Parpi0016281.t1/Parpi0003084.t1
Parpi0018106.t1/Parpi0018105.t1
Parpi0032223.t1/Parpi0004090.t1
Parpi0005857.t1/Parpi0001514.t1
Parpi0016404.t1/Parpi0025657.t1
Parpi0029740.t1/Parpi0025657.t1
Parpi0024792.t1/Parpi0017710.t1
Parpi0024994.t1/Parpi0010877.t1
Parpi0021330.t1/Parpi0014916.t1
Parpi0023804.t1/Parpi0007773.t1
Parpi0008269.t1/Parpi0020705.t1
Parpi0002005.t1/Parpi0015150.t1
Parpi0005714.t1/Parpi0008021.t1
Parpi0005786.t1/Parpi0015139.t1
Parpi0000663.t1/Parpi0000662.t1
Parpi0017984.t1/Parpi0008175.t1
Parpi0016270.t1/Parpi0014502.t1
Parpi0012107.t1/Parpi0014502.t1
Parpi0022154.t1/Parpi0013035.t1
Parpi0002630.t1/Parpi0002295.t1
Parpi0011978.t1/Parpi0015467.t1
Parpi0010911.t1/Parpi0009691.t1
Parpi0020890.t1/Parpi0018178.t1
Parpi0017992.t1/Parpi0005977.t1
Parpi0015302.t1/Parpi0009545.t1
Parpi0021916.t1/Parpi0021914.t1
Parpi0014019.t1/Parpi0014645.t1
Parpi0006915.t1/Parpi0008389.t1
Parpi0008661.t1/Parpi0015209.t1
Parpi0018689.t1/Parpi0006497.t1
Parpi0023313.t1/Parpi0018257.t1
Parpi0024429.t1/Parpi0001293.t1
Parpi0022501.t1/Parpi0002133.t1
Parpi0011600.t1/Parpi0020499.t1
Parpi0022276.t1/Parpi0003052.t1
Parpi0021935.t1/Parpi0015870.t1
Parpi0022503.t1/Parpi0019844.t1
Parpi0012725.t1/Parpi0003658.t1
Parpi0015386.t1/Parpi0016844.t1
Parpi0019656.t1/Parpi0010217.t1
Parpi0032976.t1/Parpi0003702.t1
Parpi0021494.t1/Parpi0000898.t1
Parpi0024779.t1/Parpi0018854.t1
Parpi0018008.t1/Parpi0030703.t1
Parpi0024975.t1/Parpi0000164.t1
Parpi0019389.t1/Parpi0019894.t1
Parpi0006449.t1/Parpi0003159.t1
Parpi0005750.t1/Parpi0008041.t1
Parpi0021554.t1/Parpi0011638.t1
Parpi0021553.t1/Parpi0011638.t1
Parpi0021733.t1/Parpi0013240.t1
Parpi0020829.t1/Parpi0004473.t1
Parpi0012382.t1/Parpi0017506.t1
Parpi0021042.t1/Parpi0017273.t1
Parpi0008407.t1/Parpi0016344.t1
Parpi0009756.t1/Parpi0008910.t1
Parpi0008622.t1/Parpi0017192.t1
Parpi0018195.t1/Parpi0018183.t1
Parpi0021039.t1/Parpi0016687.t1
Parpi0025824.t1/Parpi0002876.t1
Parpi0020638.t1/Parpi0010705.t1
Parpi0016438.t1/Parpi0015610.t1
Parpi0016347.t1/Parpi0021484.t1
Parpi0022921.t1/Parpi0014830.t1
Parpi0031971.t1/Parpi0005246.t1
Parpi0023286.t1/Parpi0000261.t1
Parpi0001074.t1/Parpi0003075.t1
Parpi0024789.t1/Parpi0010313.t1
Parpi0021029.t1/Parpi0001232.t1
Parpi0003630.t1/Parpi0003396.t1
Parpi0015562.t1/Parpi0013871.t1
Parpi0024778.t1/Parpi0018854.t1
Parpi0008829.t1/Parpi0012581.t1
Parpi0011356.t1/Parpi0004416.t1
Parpi0022578.t1/Parpi0004383.t1
Parpi0020418.t1/Parpi0000888.t1
Parpi0003716.t1/Parpi0006611.t1
Parpi0005881.t1/Parpi0004061.t1
Parpi0024791.t1/Parpi0013849.t1

Figure S12. Comparison of expression levels between the elusive genes and non-elusive genes

Heatmaps representing log-fold ratios of expression levels (FPKM+1) between the elusive and non-elusive genes using eleven samples containing adult tissues and embryonic stages of green anole (**a**) and those using three embryonic stages of the Madagascar ground gecko (**b**). The heatmaps were generated with the heatmap.2 library implemented in R.
