# Supplementary material for: Madagascar ground gecko genome analysis characterizes asymmetric fates of duplicated genes
Source: BMC Biol. 2018 Apr 16;16:40. doi: 10.1186/s12915-018-0509-4 (PMC5901865; doi:10.1186/s12915-018-0509-4)
Supplement: Supplementary file 16 — Figure S13. Expression profiles of FoxG genes for zebrafish embryos. (PDF 1046 kb) [file 12915_2018_509_MOESM16_ESM.pdf]

Additional file 16

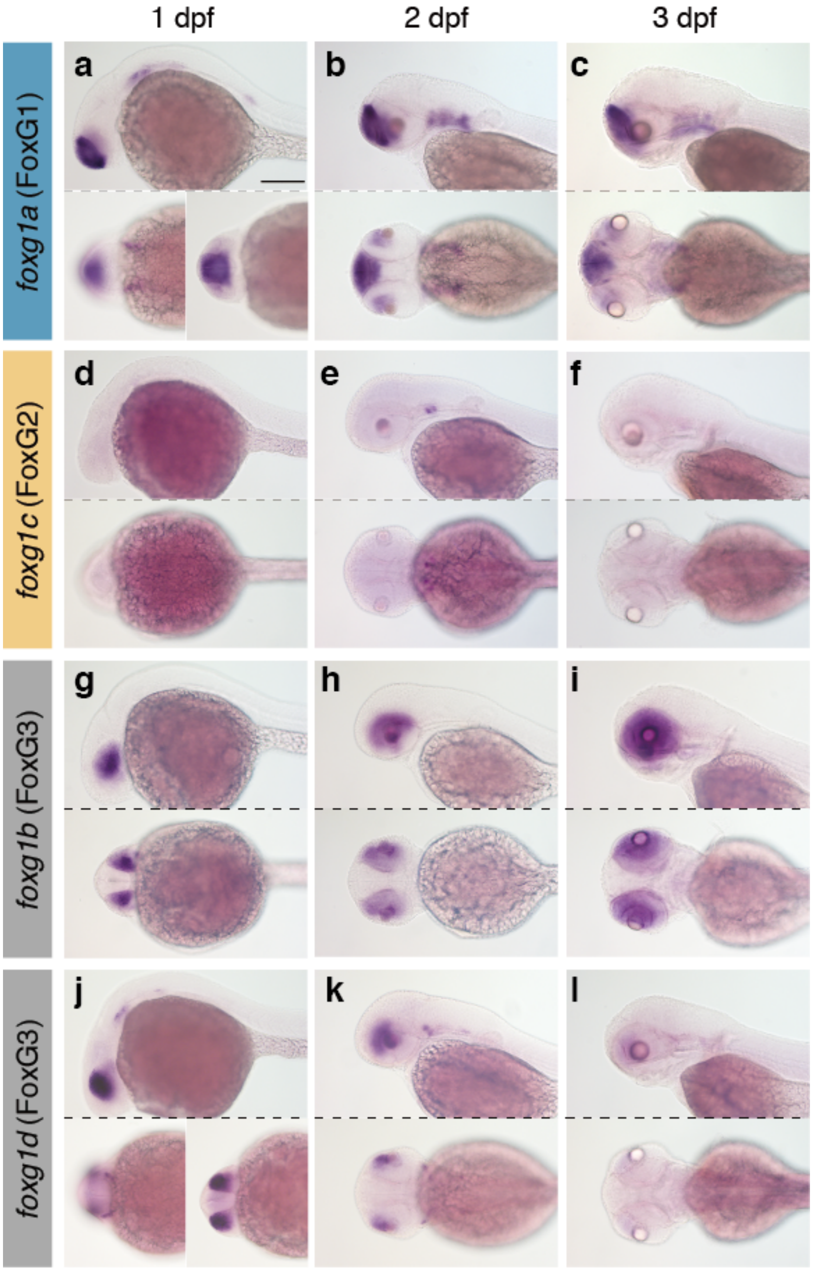

Figure S13. Expression profiles of FoxG genes for zebrafish embryos

Whole-mount in situ hybridization of *foxg1a* (**a-c**, the FoxG1 ortholog), *foxg1c* (**d-f**, the FoxG2 ortholog), and *foxg1b* (**g-i**) and *foxg1d* (**j-l**) (the FoxG3 orthologs) using 1-3 dpf zebrafish embryos. Individual embryos are displayed with lateral (upper) and dorsal (lower) views except for **g** and **h** with a ventral view and **a** and **j** with dorsal and ventral views in the lower panel.
