# Supplementary material for: Madagascar ground gecko genome analysis characterizes asymmetric fates of duplicated genes
Source: BMC Biol. 2018 Apr 16;16:40. doi: 10.1186/s12915-018-0509-4 (PMC5901865; doi:10.1186/s12915-018-0509-4)
Supplement: Supplementary file 17 — Figure S14. SNV density distribution. (PDF 145 kb) [file 12915_2018_509_MOESM17_ESM.pdf]

**Additional file 17**

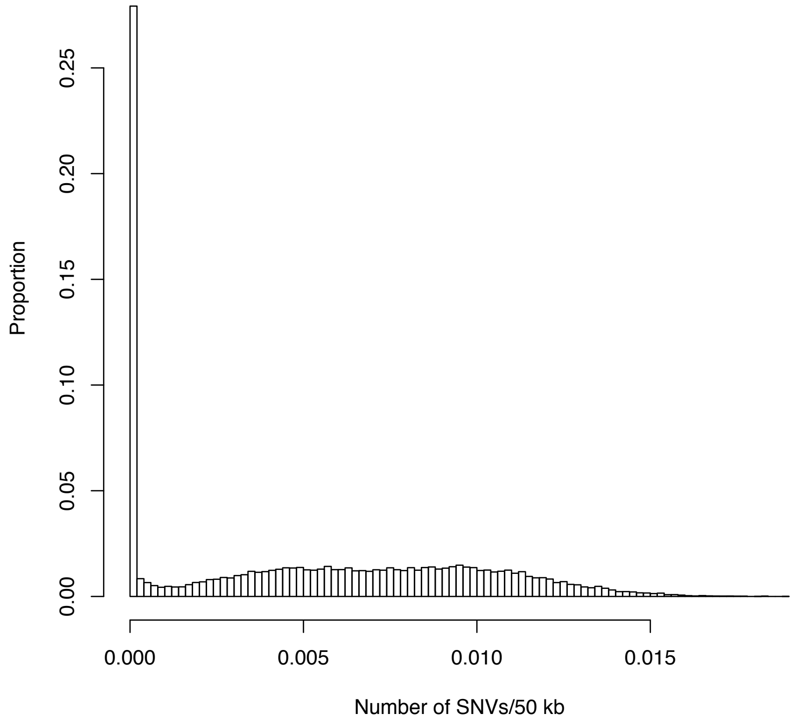


**Figure S14. SNV density distribution**

A histogram shows the number of SNVs in non-overlapped 50-kb windows across the *P. picta* genome. The distribution indicates that wild individuals of this species have a relatively high heterozygosity (~0.8 % on average), though the heterozygosity is being rapidly lost owing to inbreeding.
