# Supplementary material for: Madagascar ground gecko genome analysis characterizes asymmetric fates of duplicated genes
Source: BMC Biol. 2018 Apr 16;16:40. doi: 10.1186/s12915-018-0509-4 (PMC5901865; doi:10.1186/s12915-018-0509-4)
Supplement: Supplementary file 18 — Figure S15. Conserved noncoding elements among amniotes. (PDF 606 kb) [file 12915_2018_509_MOESM18_ESM.pdf]

Additional file 18

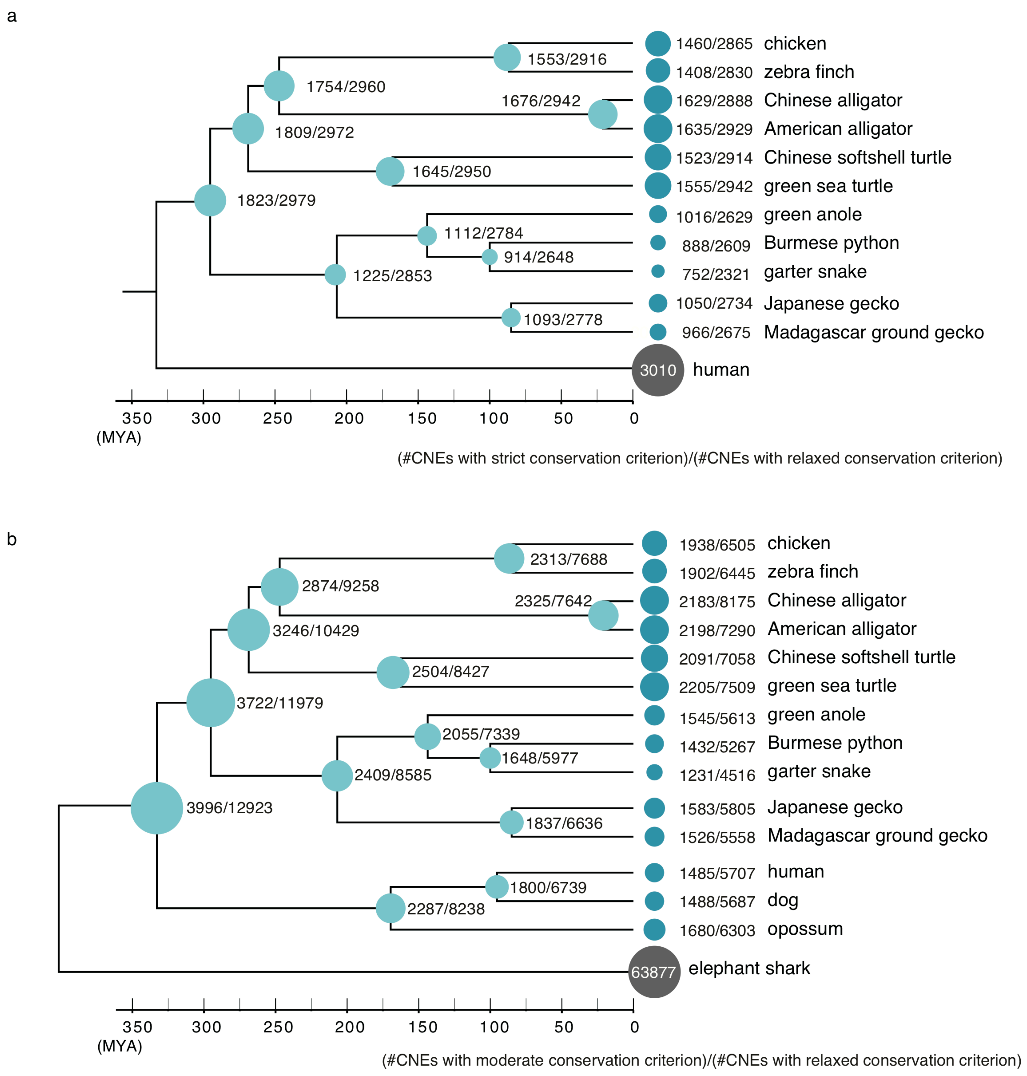

Figure S15. Conserved noncoding elements among amniotes

(**a**) Variation of retention of the human CNEs among sauropsid genomes. The human CNEs were obtained from the CONDOR database [93], in which conserved elements were identified based on the genome comparisons between the fugu and mammalian genomes. Values at the leaves of the species tree represent numbers of CNEs identified in the genome sequences based on the strict criterion (left, ≥100bp match with >95% similarity) and the relaxed criterion (right, ≥60bp match with >80% similarity). Values within light blue denote numbers of CNEs inferred in the ancestral genomes. The total number of the human CNEs is shown in a gray circle. (**b**) Variation of retention of the elephant shark CNEs among amniotes. The elephant shark CNEs were obtained from Venkatesh et al. (2014) [94]. Values at the leaves of the tree represent numbers of CNEs

identified in the genomes based on the moderate criterion (left, ≥80bp match with >85% similarity) and the relaxed criterion (right). Values within light blue circles denote numbers of CNEs inferred in the ancestral genome genomes. The total number of the elephant shark CNEs is shown in a gray circle.
