# Supplementary material for: Madagascar ground gecko genome analysis characterizes asymmetric fates of duplicated genes
Source: BMC Biol. 2018 Apr 16;16:40. doi: 10.1186/s12915-018-0509-4 (PMC5901865; doi:10.1186/s12915-018-0509-4)
Supplement: Supplementary file 19 — Figure S16. Taste receptor gene tree. (PDF 4710 kb) [file 12915_2018_509_MOESM19_ESM.pdf]

Additional file 19

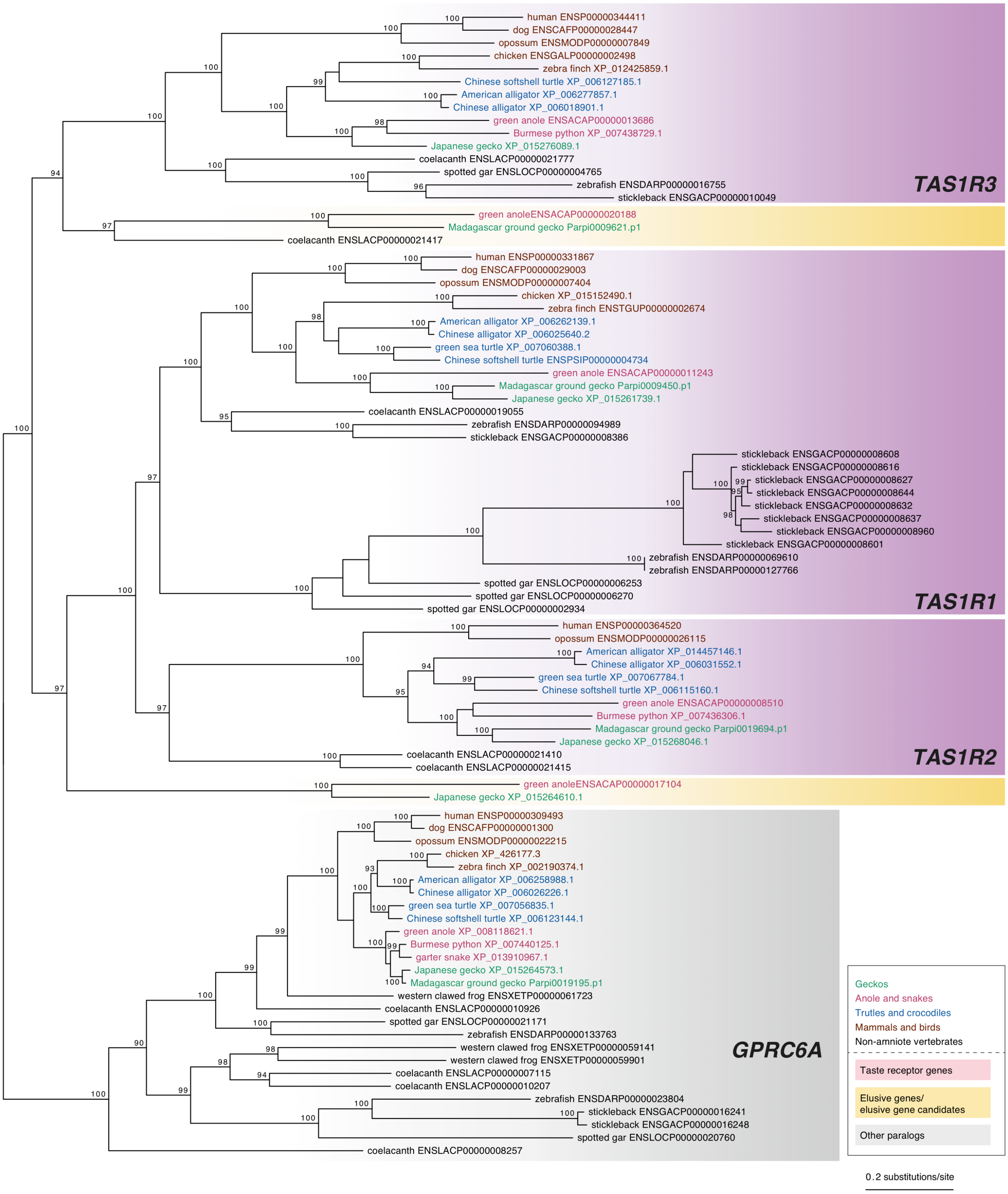

Figure S16. Taste receptor gene tree

Phylogenetic tree of taste receptor genes, belonging to the GPCR superfamily, using 560 alignment sites of the amino acid sequences of the ortholog group AmnPhylome_0000004. The tree was inferred with IQ-TREE assuming the JTT+F+R5 model. Values at the nodes represent ultrafast bootstrap approximation values with high reliability ($\geq$90). Branches of the geckos, anole/snakes, turtles/crocodiles, and avian lineages are in green, red, blue, and brown, respectively. Subtrees with pink backgrounds represent known taste receptor genes in mammals, and those with yellow backgrounds represent the genes that retain reptile orthologs but not mammals and avians ones. The subtree with a yellow background that included the orthologs of non-amniote vertebrates was defined as the elusive genes.
