# Supplementary material for: Madagascar ground gecko genome analysis characterizes asymmetric fates of duplicated genes
Source: BMC Biol. 2018 Apr 16;16:40. doi: 10.1186/s12915-018-0509-4 (PMC5901865; doi:10.1186/s12915-018-0509-4)
Supplement: Supplementary file 20 — Figure S17. Summary of contamination check for genome assembly. (PDF 146 kb) [file 12915_2018_509_MOESM20_ESM.pdf]

Additional file 20

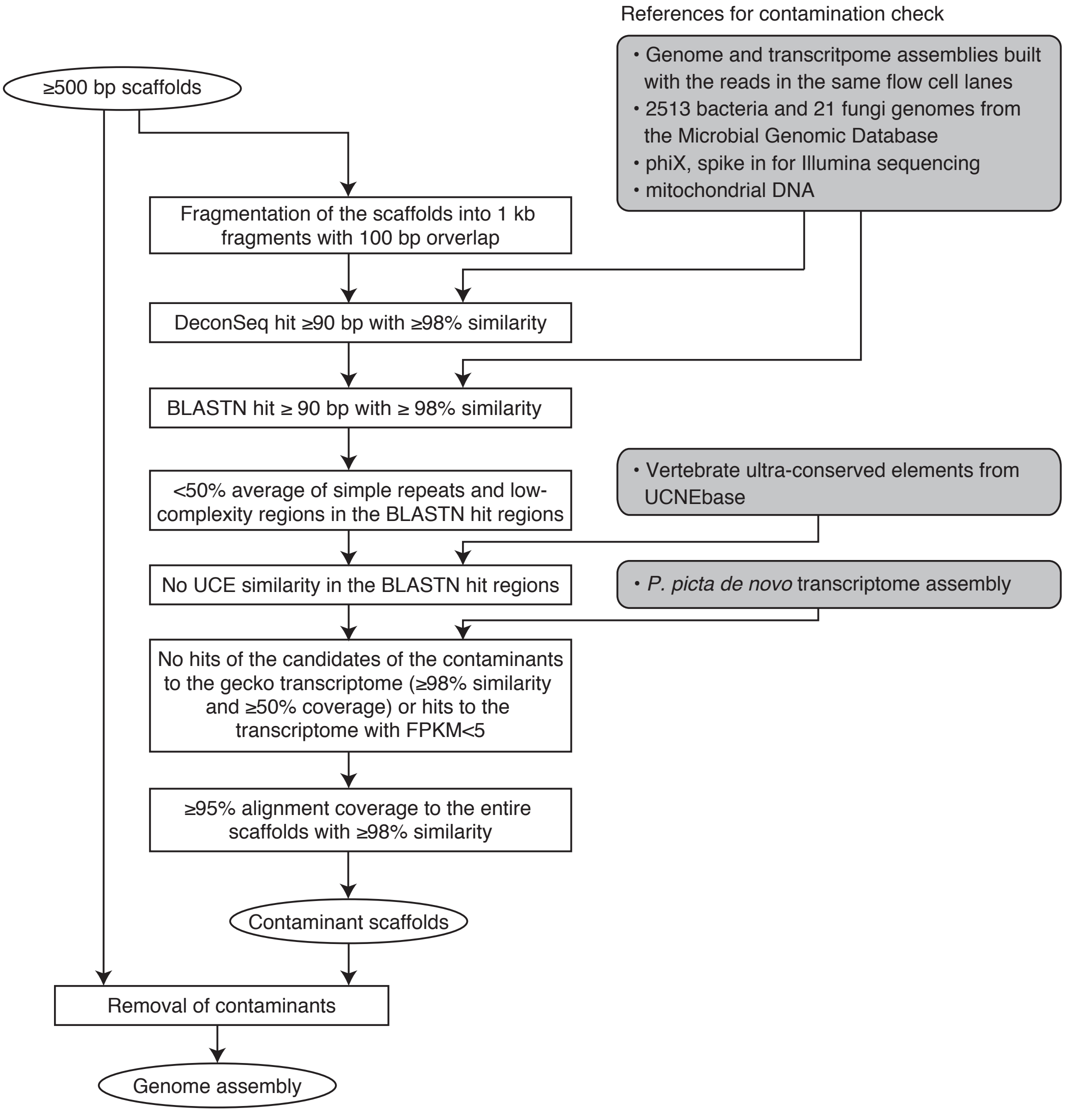

Figure S17. Summary of contamination check for genome assembly

A flowchart producing the final assembly, from which contaminant sequences are removed, is shown.
